# Comparison of two repetitive peripheral magnetic stimulation protocols for upper trapezius myofascial pain syndrome: A randomized trial

**DOI:** 10.1371/journal.pone.0360355

**Published:** 2026-10-08

**Authors:** Thitirut Jongutchariya, Surapa Hangsaphruek, Thawisak Wongkiratimethawi, Jittima Saengsuwan

**Affiliations:** 1 Department of Rehabilitation, Hatyai Hospital, Songkhla, Thailand; 2 Department of Rehabilitation Medicine, Faculty of Medicine, Khon Kaen University, Khon Kaen, Thailand; TIU: Tishk International University, IRAQ

## Abstract

**Background:**

Upper trapezius myofascial pain syndrome (MPS) is a common contributor to chronic neck pain and functional limitations. Repetitive peripheral magnetic stimulation (rPMS) has emerged as a non-invasive neuromodulatory intervention; however, the optimal stimulation duration remains uncertain. Clarifying duration-dependent effects is important for informing parameter standardization in rehabilitation practice.

**Methods:**

This single-centre, assessor-blinded randomized trial enrolled adults aged 18–55 years with chronic (≥ 3 months) upper trapezius MPS and baseline Visual Analog Scale (VAS) scores ≥ 4. Participants were randomly assigned in a 1:1 ratio using permuted blocks to receive either a 15-minute or 10-minute rPMS protocol once weekly for 4 weeks, in addition to standardized stretching exercises. The primary outcome was pain intensity measured using the VAS. Secondary outcomes included Pressure Pain Threshold (PPT) and the Neck Disability Index (NDI). Assessments were conducted at baseline, week 2, and week 4 by a blinded assessor. Analyses followed the intention-to-treat (ITT) principle using linear mixed-effects models (LMMs) with fixed effects for group, time (baseline, week 2, week 4), and the group-by-time interaction.

**Results:**

Of 83 individuals screened, 66 were randomized (33 per group) and included in the ITT analysis. Baseline characteristics were comparable between groups. LMMs demonstrated significant improvements over time in VAS, PPT, and NDI (all p < 0.001), with no significant group-by-time interactions. At the prespecified primary endpoint (week 4), VAS decreased from baseline by 4.87 points in Protocol A and 5.02 points in Protocol B. Across both groups, PPT increased by 2.51 kg/cm² and NDI decreased by 9.15 points. The adjusted between-group difference in VAS at week 4 was −0.27 (95% CI −0.95–0.40; p = 0.43), indicating no significant difference between protocols. Similarly, no significant between-group differences were observed for PPT (0.17, 95% CI −0.70–1.03; p = 0.71) or NDI (−0.64, 95% CI −3.29–2.02; p = 0.64).

**Conclusions:**

Both treatment durations were associated with significant short-term improvements in pain intensity, pressure pain threshold, and neck-related disability. No significant between-protocol differences were detected within the present sample and follow-up period. These findings suggest that a 10-minute rPMS protocol may be a practical option for clinical rehabilitation settings.

**Trial registration:**

This trial was prospectively registered with the Thai Clinical Trials Registry (Ref: TCTR20250929011; https://www.thaiclinicaltrials.org/show/TCTR20250929011).

## Introduction

Neck pain is one of the leading causes of years lived with disability (YLDs) worldwide, with Global Burden of Disease analyses projecting continued escalation through 2050 due to population ageing and modifiable risk exposure [1–3]. These epidemiological trends highlight the limitations of purely symptomatic treatment approaches and emphasize the need for mechanism-informed rehabilitation strategies. Among individuals with chronic nonspecific neck pain, myofascial pain syndrome (MPS) involving the upper trapezius is a common clinical condition [4–6]. Simons et al. described myofascial trigger points (TrPs) as hyperirritable nodules within taut bands of skeletal muscle that provoke local and referred pain and that are associated with reduced PPT [6]. Although traditionally considered a localized muscular disorder, accumulating evidence suggests that persistent nociceptive input from active TrPs contributes to central sensitization, altered motor control, impaired descending inhibition, and maladaptive neuroplastic changes associated with chronic pain [7–10].

The upper trapezius muscle is particularly vulnerable to overload because of its continuous low-level activation during postural stabilization and repetitive occupational activities. Sustained mechanical loading may contribute to metabolic stress, motor endplate dysfunction, and abnormal motor unit activity, thereby promoting the development of myofascial trigger points [11,12]. Occupational and ergonomic exposures, particularly prolonged screen-based work, have been consistently associated with neck and upper trapezius symptoms, highlighting the contribution of sustained biomechanical stress to the persistence of MPS [12,13]. Contemporary mechanistic literature further indicates that the etiopathogenesis of myofascial TrPs reflects an interaction between peripheral muscle dysfunction and central pain modulation rather than isolated muscular pathology [7,11]. Persistent peripheral nociceptive input from active TrPs may therefore maintain central sensitization within a bidirectional peripheral–central feedback loop, suggesting that effective interventions should extend beyond local tissue deactivation, particularly given evidence that TrPs can both drive and result from central sensitization processes, and that the degree of nociplastic involvement is inversely related to outcomes from peripherally directed treatment alone [8].

Current management of upper trapezius MPS primarily focuses on trigger-point deactivation and correction of contributing biomechanical factors [14,15]. Pharmacological and injection-based interventions, including local anaesthetic and trigger-point injections, may provide short-term pain relief; however, evidence supporting sustained long-term benefit remains limited [14–18]. Non-pharmacological approaches such as therapeutic exercise, manual therapy, acupuncture, and dry needling have demonstrated modest short-term improvements, although treatment responses remain variable [15]. A recent network meta-analysis reported substantial heterogeneity in the comparative effectiveness of non-invasive interventions and found no clearly superior treatment for chronic MPS, highlighting the need for mechanism-based therapeutic approaches [19].

Given the inconsistent and often transient benefits of conventional therapies, increasing attention has been directed toward technology-assisted neuromodulation, particularly repetitive peripheral magnetic stimulation (rPMS). As a non-invasive modality, rPMS delivers high-intensity time-varying electromagnetic fields capable of penetrating deep neuromuscular tissues while bypassing cutaneous nociceptors, thereby improving treatment tolerability [20,21]. Mechanistically, rPMS may modulate pain processing through preferential recruitment of large-diameter afferent fibres, inhibition of nociceptive transmission, facilitation of descending inhibitory pathways, and modulation of cortical and spinal excitability, consistent with activity-dependent neuroplasticity mechanisms [21–23]. Clinical studies and meta-analyses support its therapeutic potential across pain, neurological, and musculoskeletal (MSK) conditions, including postoperative pain [24], upper-limb motor recovery after stroke [25], and chronic low back pain [26]. In upper trapezius MPS specifically, randomized sham-controlled evidence showed that a single 10-minute rPMS session significantly reduced pain intensity and improved neck-related disability, with peak effects observed on Days 3–4 post-treatment [27]. Collectively, these findings position rPMS as a mechanistically grounded, non-invasive intervention with translational relevance for chronic upper trapezius MPS.

Despite accumulating evidence supporting the clinical utility of rPMS, implementation in routine rehabilitation remains limited by variability in stimulation parameters, particularly session duration. It has been hypothesized that neuromodulatory effects may be influenced by cumulative afferent input and total stimulation exposure [21], although the relationship between stimulation duration and clinical response remains incompletely understood. Previous dose-exploration studies have evaluated either a fixed 10-minute protocol [28] or a 15-minute combined protocol [29] in chronic low back pain populations. Although conducted in lumbar conditions, the underlying principles of activity-dependent plasticity may be applicable across musculoskeletal pain disorders. However, these studies neither directly compared treatment durations nor evaluated PPT and NDI as clinical outcomes, and they were not performed in patients with upper trapezius MPS. To date, no randomized trial has directly compared different rPMS treatment durations in individuals with upper trapezius MPS. Therefore, this randomized trial was designed to evaluate whether a longer stimulation duration (15 minutes) provides additional benefits over a shorter duration (10 minutes) with respect to pain intensity, pressure pain threshold, and neck-related disability.

## Methods

### Study design

This single-centre, single-assessor–blinded randomized trial was conducted at the Department of Rehabilitation Medicine, Hatyai Hospital, a tertiary care center in Songkhla, Thailand. The study protocol was prospectively registered with the Thai Clinical Trials Registry (Ref: TCTR20250929011; https://www.thaiclinicaltrials.org/show/TCTR20250929011) prior to participant enrolment. The trial was conducted between 1 October 2025, and 30 January 2026. Eligible participants provided written informed consent before undergoing any study procedures. Randomization was performed using a computer-generated sequence with allocation concealment. A single assessor, blinded to group assignment, conducted all outcome evaluations to minimize detection bias. The trial adopted a superiority framework to determine whether extending rPMS session duration from 10 minutes to 15 minutes would result in superior short-term clinical outcomes. Because previous sham-controlled studies have suggested short-term benefits of rPMS for upper trapezius MPS [27], the primary objective of the present study was treatment-parameter optimization rather than re-establishing efficacy against placebo. Accordingly, the trial compared two active treatment durations under pragmatic rehabilitation conditions. The study was reported in accordance with the Consolidated Standards of Reporting Trials (CONSORT) 2025 guidelines [30]. Participant flow is presented in Fig 1, and the CONSORT checklist is provided as Supporting Information (S1 File). Patients or members of the public were not involved in the design, conduct, reporting, or dissemination of this research. No protocol deviations occurred during the study.

**Fig 1:**
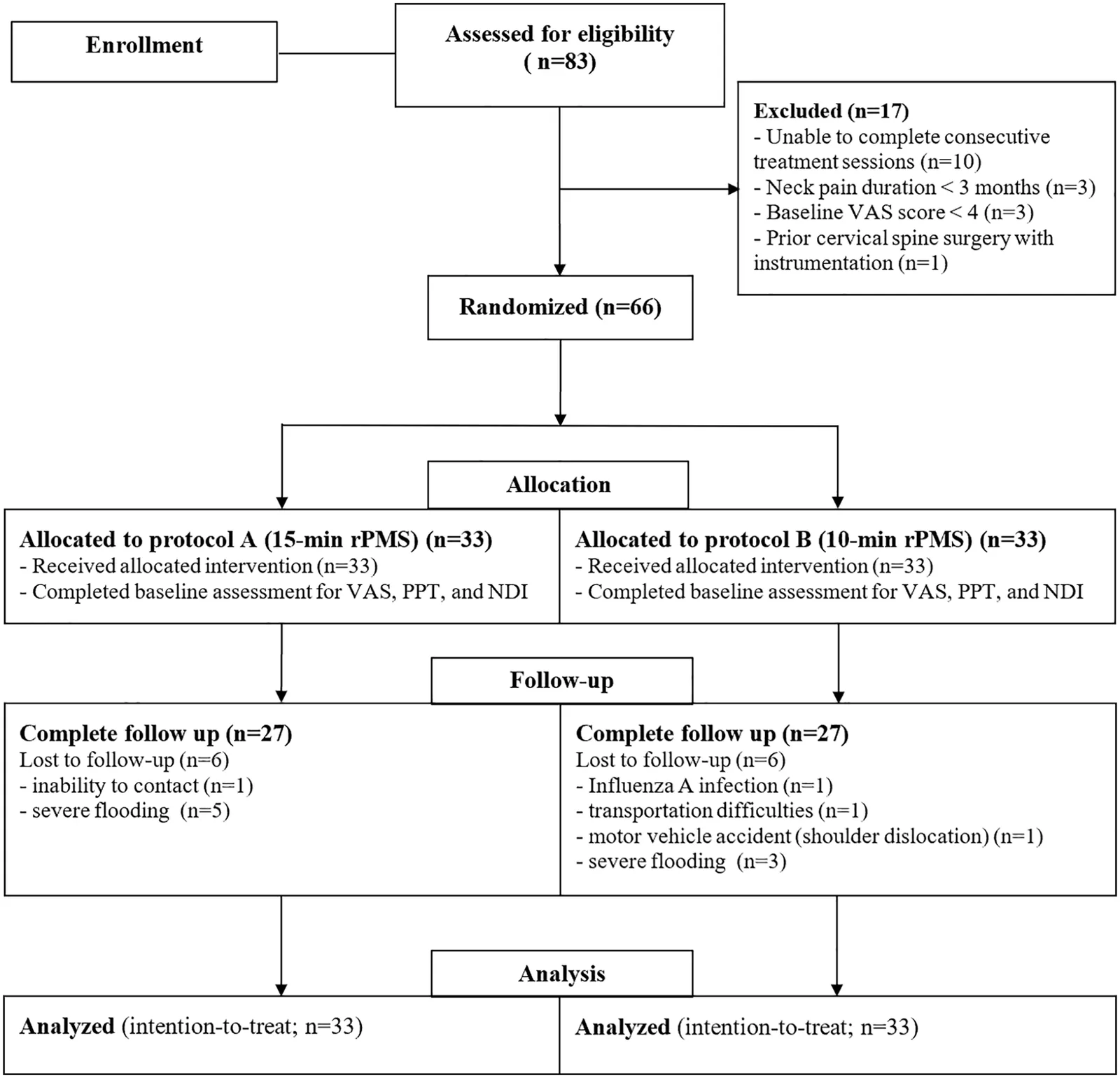
CONSORT 2025 flow diagram of progress through the study phases (All Subjects).

### Ethics statement

The study protocol was approved by the Hat Yai Hospital Research Ethics Committee (approval No. HYH EC 061-68-01) on 14 August 2025. Ethical approval was granted following review of the study protocol and supporting documents prior to study initiation. All participants provided written informed consent before enrolment. The study was conducted in accordance with the Declaration of Helsinki and Good Clinical Practice guidelines.

### Participants

Participants diagnosed with upper trapezius MPS according to the diagnostic criteria described by Simons et al. [6,11,31] were recruited from the Department of Rehabilitation Medicine, Hatyai Hospital. Diagnosis was confirmed by a board-certified rehabilitation physician prior to enrolment. Potential participants were identified from the registry of new admissions and underwent initial eligibility screening by attending physicians or trained clinical staff. Eligibility and diagnosis were subsequently confirmed by a board-certified rehabilitation physician prior to enrolment. Eligible individuals received a detailed explanation of the study procedures from a trained research assistant, and written informed consent was obtained before enrolment.

Inclusion criteria were: (1) men or women aged 18–55 years; (2) chronic pain duration ≥ 3 months; (3) baseline pain intensity ≥ 4 on the VAS; (4) ability to provide informed consent; and (5) proficiency in the Thai language. Exclusion criteria were: (1) specific cervical spine pathology or neurological deficits; (2) neuromuscular or systemic inflammatory disease; (3) prior cervical spine surgery; (4) pregnancy or breastfeeding; (5) fibromyalgia; (6) implanted electronic or metallic devices; and (7) contraindications to nonsteroidal anti-inflammatory drugs. Participants were withdrawn from the study if they experienced a serious adverse event or failed to adhere to the study protocol, or if they voluntarily withdrew consent.

### Sample size

Sample size was determined for a two-arm randomized trial using an analysis of covariance (ANCOVA) model with baseline adjustment, following the approach described by Borm et al. [32]. The calculation was based on the prespecified primary outcome, pain intensity measured using the VAS, assessed at baseline and at weeks 2 and 4. Pain intensity measured by the VAS was designated a priori as the primary endpoint and served as the basis for sample size estimation. A between-group difference of 0.5 points on the VAS was selected as a conservative design assumption for detecting a small incremental treatment effect between two active rPMS duration protocols rather than as a minimal clinically important difference (MCID). This assumption was informed by previous rPMS studies conducted in musculoskeletal pain populations, including chronic low back pain [29], which suggested that greater stimulation exposure might provide additional clinical benefit. However, no previous randomized trial had directly compared different rPMS treatment durations in patients with upper trapezius MPS. Therefore, the assumed between-group difference was considered a pragmatic and conservative estimate for study planning purposes rather than an established treatment effect. Because both groups received active rPMS combined with standardized stretching exercises, only a modest between-group difference was expected. The assumed standard deviation of 0.8 was based on variability estimates reported in previous rPMS studies [28]. A baseline-to-follow-up correlation coefficient of 0.6 was assumed, consistent with values reported for musculoskeletal patient-reported outcomes by Walters et al. [33]. Using a two-sided significance level of 0.05 and 80% power, the required sample size was estimated at 26 participants per group. To account for an anticipated attrition rate of 20%, the target sample size was increased to 33 participants per group, resulting in a total planned sample of 66 participants. Although the primary analyses were performed using linear mixed-effects models (LMMs) with restricted maximum likelihood (REML) estimation, the ANCOVA-based sample size approach was considered appropriate because both methods account for baseline measurements and within-subject correlation when analyzing longitudinal continuous outcomes.

### Randomization and blinding

Participants were randomly assigned in a 1:1 ratio to two intervention groups using permuted block randomization with a fixed block size of four. A total of 66 participants were allocated equally between the two groups (n = 33 per group). The allocation sequence was generated using a computerized randomization programme, with assignments randomly permuted within each block to maintain group balance and reduce predictability. Allocation concealment was ensured by entrusting the master randomization list to an independent research assistant who was not involved in participant recruitment, enrolment, or outcome assessment. Group assignments were disclosed only after participant enrolment. Participant blinding was not feasible given that it is impossible to blind participants to these types of interventions. In addition, the treatment duration between the two groups differed. To minimize expectation-related bias, participants were not informed of the study hypothesis or of any anticipated superiority between treatment protocols. Therefore, the study adopted an assessor-blinded design, and all outcome assessments were performed by an investigator who remained unaware of treatment allocation throughout the study.

### Interventions

All participants received standardized conservative management in addition to their assigned rPMS protocol to ensure consistency of background care across treatment groups.

#### Standard treatment.

Participants received ergonomic education focused on daily activity modification and were instructed in a standardized bilateral upper trapezius self-stretching programme. Each stretch was held for 10 seconds and repeated 10 times per set, with two sets performed daily throughout the 4-week study period.

#### Repetitive peripheral magnetic stimulation (rPMS).

rPMS was administered once weekly for four consecutive weeks using a high-intensity peripheral magnetic stimulator (Salus Talent Pro; REMED Rehabilitation Medical Company). The study compared two treatment durations based on previous studies: a 15-minute protocol [29] and a 10-minute protocol [28].

Participants assigned to Protocol A (15 minutes) received a two-phase stimulation sequence with progressive intensity titration. Phase 1 consisted of scanning stimulation applied to the upper trapezius region for 10 minutes using a dynamic frequency range of 3 Hz to 30 Hz to provide broad neuromodulatory stimulation. Stimulation intensity was initiated at 20% of the maximum stimulator output and gradually increased in increments of less than 5% until the participant’s maximum comfortable, non-painful threshold was reached. Phase 2 consisted of targeted TrP stimulation delivered for 5 minutes to the primary myofascial TrP using a fixed frequency of 30 Hz with an intermittent duty cycle of 1 second on and 3 seconds off. Intensity was gradually titrated according to participant tolerance.

Participants assigned to Protocol B (10 minutes) received an identical stimulation sequence with a shorter scanning phase. Phase 1 consisted of scanning stimulation applied for 5 minutes using the same stimulation parameters as Protocol A. Phase 2 consisted of 5 minutes of targeted TrP stimulation delivered to the primary myofascial TrP using identical frequency and duty-cycle settings to those used in Protocol A.

#### Rescue medication.

Participants were instructed to use nonpharmacological strategies, including stretching or cold application, to manage acute pain exacerbations. If symptoms persisted, oral diclofenac 25 mg was permitted as rescue medication. All use of rescue medication was recorded throughout the study to assess its potential influence on outcome measures.

#### Adverse event monitoring.

Participants were routinely asked about treatment-related symptoms during each treatment session, including discomfort during stimulation, muscle soreness, headache, abnormal skin sensations, dizziness, and treatment intolerance.

### Outcomes and measurements

### Baseline data collection

After enrolment, comprehensive baseline demographic and clinical data were recorded. A board-certified rehabilitation physician conducted a standardized physical examination to confirm the diagnosis of upper trapezius MPS and to identify active myofascial TrP. Sociodemographic, occupational, and pain-related variables were collected through a structured self-administered questionnaire incorporated into the case record form. Recorded variables included age, sex, height, body mass, body mass index (BMI), smoking status, alcohol consumption, marital status, physical activity level, underlying medical conditions, daily working hours, baseline analgesic use, pain duration, clinical characteristics of upper trapezius MPS, and the side of treatment.

### Pain outcome measures

#### Primary outcome.

**1. Pain Intensity (Visual Analog Scale, VAS);** Pain intensity was assessed using the VAS, a validated instrument consisting of a 10-cm horizontal line anchored by the descriptors “no pain” and “worst imaginable pain” [34]. Participants indicated the point on the line that best represented their current pain intensity, and scores were determined by measuring the distance in millimetres from the left anchor, yielding values from 0 mm to 100 mm. The VAS is widely used in clinical and MSK research because of its simplicity, ease of administration, and sensitivity to changes in pain severity following therapeutic interventions [34,35]. Although pain ratings are inherently subjective and may vary according to individual perception, the VAS demonstrates high reliability for pain assessment in adult populations and remains a widely used outcome measure in MSK pain and rehabilitation research [36,37].

#### Secondary outcomes.

**2. Pressure Pain Threshold (PPT);** PPT was used to quantify mechanical pain sensitivity and was defined as the minimum pressure at which the sensation of pressure transitions to pain [38]. Measurements were obtained using a mechanical algometer (PainTest™ FPN 100; Wagner Instruments, Greenwich, CT, USA) equipped with a 1-cm² rubber probe. The probe was applied perpendicularly over the most symptomatic upper trapezius TrP, and pressure was gradually increased at approximately 1 kg/cm² per second until participants reported the first sensation of pain. Three consecutive measurements were obtained with a 30-second interval, and the mean value was used for analysis. Higher PPT values indicate reduced mechanical sensitivity. Pressure algometry has demonstrated strong reliability for MSK assessment, including excellent intra-rater reliability in MPS populations [39].
**3. Neck Disability Index (NDI);** Neck-related disability was assessed using the Thai version of the Neck Disability Index (NDI-TH), which has been cross-culturally adapted and validated for use in Thai populations [40]. The NDI consists of 10 items assessing pain intensity and functional activities, each scored from 0 (no disability) to 5 (complete disability), yielding total scores ranging from 0 points to 50 points (or 0% to 100%). Higher scores indicate greater functional limitation. The instrument demonstrates strong internal consistency (Cronbach’s alpha 0.85 to 0.93) and high test–retest reliability (ICC 0.74 to 0.99), with moderate to strong concurrent validity relative to pain intensity measures [40,41].

### Data collection and follow-up

Pain-related outcomes, including the VAS, PPT, and the NDI, were assessed by a blinded outcome assessor who was not involved in treatment administration. Evaluations were conducted at baseline and at weeks 2 and 4. The week 2 assessment was prespecified to capture interim response patterns and to evaluate potential nonlinear changes in clinical outcomes over time. The primary efficacy endpoint was prespecified at week 4.

### Statistical analysis

All analyses were conducted using Stata version 18.0 (StataCorp LLC, College Station, TX, USA) according to the ITT principle. All randomized participants were included in the primary analysis regardless of treatment completion status. Missing outcome data were handled using likelihood-based linear mixed-effects models estimated by restricted maximum likelihood (REML), which incorporate all available observations under the missing-at-random (MAR) assumption without requiring complete-case exclusion [42]. A per-protocol analysis was not prespecified and was not performed. Baseline characteristics were compared using chi-squared or Fisher exact tests for categorical variables and Student’s t-tests or Mann–Whitney U tests for continuous variables, as appropriate. Pain intensity measured by the VAS was prespecified as the primary outcome and served as the basis for sample size estimation. PPT and NDI were prespecified as secondary outcomes. Because the primary hypothesis concerned the VAS outcome, no formal adjustment for multiplicity was applied. Longitudinal outcomes (VAS, PPT, and NDI) were analyzed using LMMs with REML estimation. Fixed effects included group, time (baseline, week 2, week 4), and the group-by-time interaction. A participant-level random intercept was specified to account for within-subject correlation. Random-slope models were explored but not retained because of unstable variance estimates and poorer model fit. Model selection was based on Akaike’s Information Criterion (AIC) and Bayesian Information Criterion (BIC). Adjusted marginal means were estimated from the LMMs, and between-group differences at each visit were derived using contrasts of model-based marginal means. To evaluate robustness, sensitivity analyses were conducted using multiple imputation by chained equations (MICE). Twenty imputed datasets (m = 20) were generated using linear regression models. The imputation model included treatment group, baseline demographic variables (age, sex, and body mass index), baseline outcome measures, and all available follow-up outcome measurements (VAS, PPT, and NDI). Parameter estimates were pooled according to Rubin’s rules. The MAR assumption was considered plausible because missing data were primarily attributable to external and logistical factors rather than to treatment response or clinical outcomes. In addition, missing assessments were balanced across treatment groups, and baseline demographic and clinical variables were available for all participants and incorporated into both the analytical and imputation models. Sensitivity analyses yielded results consistent with the primary analyses; specifically, the group-by-time interaction remained non-significant across all outcomes. All statistical tests were two-sided, with statistical significance defined as p < 0.05.

## Results

### Participant flow

A total of 83 individuals were screened for eligibility, of whom 17 were excluded (10 were unable to complete consecutive treatment sessions, 3 had neck pain duration < 3 months, 3 had baseline VAS scores < 4, and 1 had prior cervical spine surgery with instrumentation). The remaining 66 participants were randomized equally to Protocol A (15-minute rPMS; n = 33) or Protocol B (10-minute rPMS; n = 33). Overall, 12 participants (6 per group) discontinued the intervention after randomization, resulting in 27 participants per group completing the study per protocol. Outcome data were complete for all participants at baseline. Missing assessments occurred in 2 participants (3.0%) at week 2 and 12 participants (18.2%) at week 4 and were balanced across treatment groups. Missing assessments were primarily attributable to inability to continue treatment, severe flooding, influenza A infection, transportation difficulties, and a motor vehicle accident. No participant withdrew because of treatment-related adverse events. Across all outcomes and assessment time points, 14 observations were missing out of 198 expected observations; however, all 66 randomized participants were included in the primary analysis according to the ITT principle. Participant flow is illustrated in the CONSORT 2025 diagram (Fig 1). Participants were monitored for adverse events throughout the intervention period. No serious adverse events or treatment-related discontinuations occurred. No clinically significant treatment-related adverse effects requiring medical attention, treatment modification, or study withdrawal were reported.

### Baseline characteristics

Baseline sociodemographic and clinical characteristics were comparable between groups, with no significant differences observed across variables (Table 1). In total, 42 of 66 participants (63.6%) were female, with identical proportions in Protocol A and Protocol B (63.6% each; p = 1.000). The overall mean age was 39.0 (SD 8.2) years, and the mean body mass index was 24.8 (SD 4.8) kg/m², with no significant between-group differences. Pain duration categories were also similarly distributed between groups, with 66.7% of participants reporting symptoms lasting ≥ 6 months. Baseline analgesic use was reported by 24.2% of participants and did not differ significantly between groups. The side of rPMS treatment was evenly distributed (p = 0.80). No significant differences were observed in baseline outcome measures, including VAS (mean 6.7 [SD 1.1] vs. 6.5 [SD 1.3]; p = 0.69), PPT (mean 6.7 [SD 1.8] vs. 6.9 [SD 1.8]; p = 0.71), and NDI (median 13 [IQR 8–19] vs. 14 [IQR 11–17]; p = 0.41).

**Table 1:** Patient characteristics (n = 66).

Characteristics	rPMS Protocol A (n = 33)	rPMS Protocol B (n = 33)	Total (n = 66)	p
**Socio-demographic data**				
Female, n (%)	21 (63.6)	21 (63.6)	42 (63.6)	1.00
Age (years), mean (SD)	39.2 (8.1)	38.8 (8.5)	39.0 (8.2)	0.85
BMI (kg/m^2^), mean (SD)	24.8 (4.7)	24.8 (5.0)	24.8 (4.8)	0.99
Smoking behavior (smoker), n (%)	2 (6.1)	1 (3.0)	3 (4.5)	1.00^a^
Alcohol consumption (current drinker), n (%)	4 (12.1)	4 (12.1)	8 (12.1)	1.00^a^
Underlying diseases n (%)	12 (36.4)	10 (30.3)	22 (33.3)	0.60
Hypertension	3 (9.1)	1 (3.0)	4 (6.1)	0.61^a^
Type 2 diabetes mellitus	0 (0.0)	1 (3.0)	1 (1.5)	1.00^a^
Dyslipidemia	5 (15.2)	3 (9.1)	8 (12.1)	0.71^a^
Musculoskeletal pain	9 (27.3)	4 (12.1)	13 (19.7)	0.22^a^
Working hours per day n (%)				0.37
- ≤ 8 hours/day	8 (24.2)	13 (39.4)	21 (31.8)	
- > 8 to < 10 hours	17 (51.5)	15 (45.5)	32 (48.5)	
- ≥ 10 to < 16 hours	8 (24.2)	5 (15.2)	13 (19.7)	
Physical activity n (%)				0.96
Physically inactive	9 (27.3)	8 (24.2)	17 (25.8)	
Irregular physical activity (< 3 sessions/week)	16 (48.5)	17 (51.5)	33 (50.0)	
Regular physical activity (≥ 3 sessions/week)	8 (24.2)	8 (24.2)	16 (24.2)	
**Pain characteristics**				
Duration of pain n (%)				0.40
- ≥ 3 to < 6 months	10 (30.3)	12 (36.4)	22 (33.3)	
- ≥ 6 to < 24 months	11 (33.3)	14 (42.4)	25 (37.9)	
- ≥ 24 months	12 (36.4)	7 (21.2)	19 (28.8)	
Analgesic use at baseline, n (%)	10 (30.3)	6 (18.2)	16 (24.2)	0.25
Nonsteroidal anti-inflammatory drugs	3 (9.1)	1 (3.0)	4 (6.1)	0.61^a^
Acetaminophen	2 (6.1)	4 (12.1)	6 (9.1)	0.67^a^
Weak opioids	3 (9.1)	2 (6.1)	5 (7.6)	1.00^a^
Adjuvant analgesics (gabapentin, pregabalin)	4 (12.1)	0 (0.0)	4 (6.1)	0.11^a^
Side of rPMS treatment, n (%)				0.80
- Right	21 (63.6)	20 (60.6)	41 (62.1)	
- Left	12 (36.4)	13 (39.4)	25 (37.9)	
Rescue medication (diclofenac) use, n (%)	0 (0.0)	1 (3.0)	1 (1.5)	1.00^a^
Baseline VAS (scores), mean (SD)	6.7 (1.1)	6.5 (1.3)	6.6 (1.2)	0.69
Baseline PPT (kg/cm²), mean (SD)	6.7 (1.8)	6.9 (1.8)	6.8 (1.8)	0.71
Baseline NDI (scores), median (IQR)	13 (8-19)	14 (11-17)	14 (10-18)	0.41^b^

BMI, body mass index; IQR, interquartile range; MPS, myofascial pain syndrome; NDI, Neck Disability Index; PPT, pressure pain threshold; rPMS, repetitive peripheral magnetic stimulation; SD, standard deviation; VAS, Visual Analog Scale.

^a^Fisher’s exact test. ^b^Mann–Whitney U test.

### Postintervention outcomes

#### Pain intensity (VAS).

There was no significant between-group difference at baseline (β = −0.12, 95% CI −0.74–0.50; p = 0.70) (Table 2). The group-by-time interaction was not statistically significant at week 2 (β = 0.18, 95% CI −0.53–0.88; p = 0.62) or week 4 (β = −0.15, 95% CI −0.90–0.59; p = 0.69), indicating no differential treatment effect (Table 2). A significant main effect of time was observed. Compared with baseline, VAS decreased by 3.02 points at week 2 (95% CI −3.52 – −2.52; p < 0.001) and by 4.87 points at week 4 (95% CI −5.39 to −4.34; p < 0.001) (Table 2). Adjusted marginal means declined from 6.67 (95% CI 6.23–7.10) to 1.80 (95% CI 1.32–2.28) in Protocol A and from 6.55 (95% CI 6.10–6.99) to 1.53 (95% CI 1.05–2.00) in Protocol B (Table 3). At the prespecified primary endpoint (week 4), visit-specific contrasts were examined to estimate adjusted between-group differences (Table 4). The adjusted between-group difference at Week 4 was −0.27 (95% CI −0.95–0.40; p = 0.43), confirming no significant difference between groups (Table 4). As illustrated in Fig 2A, trajectories were parallel with overlapping 95% confidence intervals.

**Table 2:** Fixed-effect estimates from LMMs with REML estimation for group, time, and group-by-time interaction (ITT principle).

Outcome	Fixed Effect	Adjusted mean difference	95% CI	p
		(β Coefficient)		
**VAS (scores)**	Group (Protocol B vs Protocol A) at Baseline	−0.12	−0.74–0.50	0.70
	Group × Time (Week 2)	0.18	−0.53–0.88	0.62
	Group × Time (Week 4)	−0.15	−0.90–0.59	0.69
	Time: Week 2 vs Baseline	−3.02	−3.52 to −2.52	< 0.001
	Time: Week 4 vs Baseline	−4.87	−5.39 to −4.34	< 0.001
**PPT (kg/cm²)**	Group (Protocol B vs Protocol A) at Baseline	0.17	−0.66–1.00	0.69
	Group × Time (Week 2)	−0.24	−0.88–0.39	0.45
	Group × Time (Week 4)	−0.00	−0.68–0.67	0.99
	Time: Week 2 vs Baseline	1.37	0.92–1.82	< 0.001
	Time: Week 4 vs Baseline	2.51	2.03–2.99	< 0.001
**NDI (scores)**	Group (Protocol B vs Protocol A) at baseline	0.39	−2.12–2.91	0.76
	Group × Time (Week 2)	−0.70	−2.87–1.47	0.53
	Group × Time (Week 4)	−1.03	−3.34–1.28	0.38
	Time: Week 2 vs Baseline	−5.85	−7.39 to −4.32	< 0.001
	Time: Week 4 vs Baseline	−9.15	−10.78 to −7.51	< 0.001

β coefficients were estimated from linear mixed-effects models (LMMs) with restricted maximum likelihood (REML) estimation. Fixed effects included group, time, and the group-by-time interaction, with a participant-level random intercept to account for within-subject correlation. Analyses followed the intention-to-treat (ITT) principle.

**Table 3:** Adjusted marginal means of VAS, PPT, and NDI across study time estimated from LMMs with REML estimation (ITT principle).

Outcome	Time	rPMS Protocol A	rPMS Protocol B
		Adjusted marginal mean (95% CI)	Adjusted marginal mean (95% CI)
**VAS (scores)**	Baseline	6.67 (6.23–7.10)	6.55 (6.10–6.99)
	Week 2	3.65 (3.20–4.10)	3.70 (3.26–4.15)
	Week 4	1.80 (1.32–2.28)	1.53 (1.05–2.00)
**PPT (kg/cm²)**	Baseline	6.73 (6.15–7.32)	6.90 (6.31–7.48)
	Week 2	8.10 (7.51–8.68)	8.02 (7.43–8.61)
	Week 4	9.24 (8.63–9.85)	9.41 (8.79–10.02)
**NDI (scores)**	Baseline	14.27 (12.49–16.05)	14.67 (12.89–16.45)
	Week 2	8.42 (6.62–10.21)	8.11 (6.32–9.91)
	Week 4	5.13 (3.25–7.01)	4.49 (2.61–6.37)

Values are adjusted marginal means (95% CI) estimated from LMMs with REML estimation. Models included fixed effects for group, time, and the group-by-time interaction, with a participant-level random intercept to account for within-subject correlation.

**Table 4:** Adjusted between-group differences across study visits derived from model-based marginal mean contrasts (ITT principle).

Outcome	Time	Adjusted between-group difference	95% CI	p
		(Protocol B − Protocol A)		
**VAS (scores)**	Baseline	−0.12	−0.74–0.50	0.70
	Week 2	0.06	−0.58–0.69	0.86
	Week 4	−0.27	−0.95–0.40	0.43
**PPT (kg/cm²)**	Baseline	0.17	−0.66–1.00	0.69
	Week 2	−0.07	−0.91–0.76	0.86
	Week 4	0.17	−0.70–1.03	0.71
**NDI (scores)**	Baseline	0.39	−2.12–2.91	0.76
	Week 2	−0.31	−2.85–2.23	0.81
	Week 4	−0.64	−3.29–2.02	0.64

Values represent adjusted between-group contrasts derived from model-based marginal means estimated from LMMs with REML estimation. Models included fixed effects for group, time, and the group-by-time interaction, with a participant-level random intercept to account for within-subject correlation.

**Fig 2:**
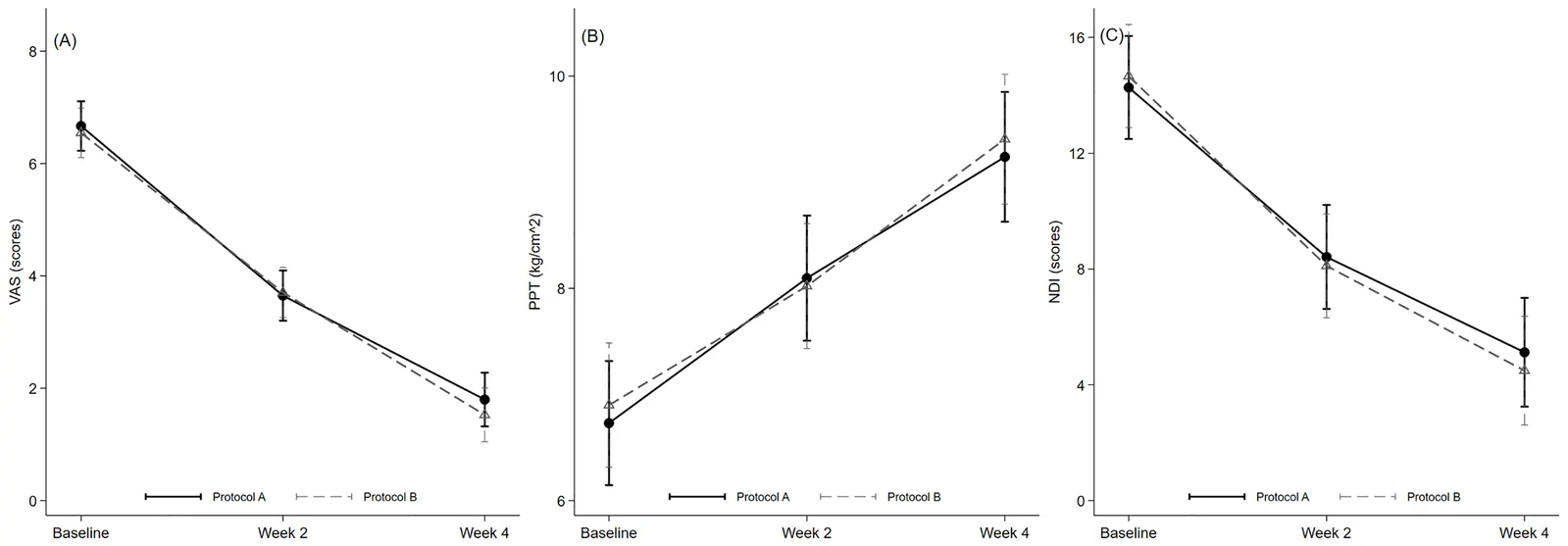
Longitudinal effects of rPMS protocols on clinical outcomes. Adjusted marginal means were estimated from LMMs with REML estimation under the intention-to-treat (ITT) principle. Error bars represent 95% confidence intervals. (A) Visual Analog Scale (VAS) scores decreased significantly over time in both groups. (B) Pressure Pain Threshold (PPT) increased progressively over time in both groups. (C) Neck Disability Index (NDI) scores decreased over the follow-up period in both groups. Parallel trajectories with overlapping 95% confidence intervals are consistent with the absence of a significant group-by-time interaction.

#### Pressure pain threshold (PPT).

There was no significant between-group difference at baseline (β = 0.17, 95% CI −0.66–1.00; p = 0.69) (Table 2). The group-by-time interaction was not significant at week 2 (β = −0.24, 95% CI −0.88–0.39; p = 0.45) or week 4 (β = 0.00, 95% CI −0.68–0.67; p = 0.99), indicating no differential treatment effect (Table 2). A significant main effect of time was observed. Compared with baseline, PPT increased by 1.37 kg/cm² at week 2 (95% CI 0.92–1.82; p < 0.001) and by 2.51 kg/cm^2^ at week 4 (95% CI 2.03–2.99; p < 0.001) (Table 2). Adjusted marginal means increased from 6.73 (95% CI 6.15–7.32) at baseline to 9.24 (95% CI 8.63–9.85) at week 4 in Protocol A, and from 6.90 (95% CI 6.31–7.48) to 9.41 (95% CI 8.79–10.02) in Protocol B (Table 3). The adjusted between-group difference at week 4 was 0.17 (95% CI −0.70–1.03; p = 0.71) (Table 4). As illustrated in Fig 2B, trajectories were parallel with overlapping 95% confidence intervals.

#### Neck disability index (NDI).

There was no significant between-group difference at baseline (β = 0.39, 95% CI −2.12–2.91; p = 0.76) (Table 2). The group-by-time interaction was not significant at week 2 (β = −0.70, 95% CI −2.87–1.47; p = 0.53) or week 4 (β = −1.03, 95% CI −3.34–1.28; p = 0.38), indicating comparable treatment effects (Table 2). A significant main effect of time was observed. Compared with baseline, NDI decreased by 5.85 points at week 2 (95% CI −7.39 to −4.32; p < 0.001) and by 9.15 points at week 4 (95% CI −10.78 to −7.51; p < 0.001) (Table 2). Adjusted marginal means declined from 14.27 (95% CI 12.49–16.05) at baseline to 5.13 (95% CI 3.25–7.01) at week 4 in Protocol A, and from 14.67 (95% CI 12.89–16.45) to 4.49 (95% CI 2.61–6.37) in Protocol B (Table 3). The adjusted between-group difference at week 4 was −0.64 (95% CI −3.29–2.02; p = 0.64), indicating no significant difference between groups (Table 4). As shown in Fig 2C, both protocols demonstrated nearly parallel reductions over time.

### Overall treatment effects

Across VAS, PPT, and NDI, LMMs demonstrated significant time effects, indicating substantial clinical improvements over the 4-week intervention period (Table 2). Adjusted marginal means showed consistent improvement in both groups (Table 3), with parallel longitudinal trajectories and overlapping 95% CI (Fig 2). No significant group effect or group-by-time interaction was observed for any outcome, indicating that the trajectory of improvement did not differ between groups. Between-group contrasts derived from model-based marginal means confirmed no significant differences between groups at any visit (Table 4).

## Discussion

### Overall findings

This randomized trial evaluated whether extending rPMS session duration from 10 to 15 minutes improves clinical outcomes in individuals with upper trapezius MPS. LMMs demonstrated significant time effects for VAS, PPT, and NDI (all p < 0.001), indicating significant improvements across the 4-week intervention period. However, no significant group-by-time interaction was observed for any outcome, suggesting that both stimulation durations produced comparable recovery trajectories. No serious adverse events related to rPMS were reported, supporting the safety and tolerability of the intervention under the applied stimulation parameters. Importantly, because both groups received active rPMS combined with standardized stretching exercises, the present findings should be interpreted as a comparison between two active treatment protocols rather than as evidence confirming the efficacy of rPMS itself.

To our knowledge, this is the first randomized trial to directly compare two rPMS session durations for upper trapezius MPS. The findings are consistent with previous studies demonstrating analgesic and functional benefits of rPMS in musculoskeletal pain conditions [21,22,24–27]. Earlier investigations used heterogeneous stimulation parameters and did not isolate session duration as an independent treatment variable [21,43]. For example, Smania et al. [23] reported significant improvements in pain, PPT, and trigger-point characteristics following multi-session trapezius rPMS, with benefits persisting for up to three months. Similarly, Mahisanun and Saengsuwan [27] demonstrated significant reductions in VAS and NDI following a single 10-minute rPMS session compared with sham stimulation in patients with neck MPS. Studies in chronic low back pain have likewise reported favourable outcomes following both 10-minute [28] and 15-minute [29] stimulation protocols, although direct duration comparisons were not performed. Taken together, the parallel improvement trajectories observed in the present study suggest that effective engagement of sensitized trigger-point tissue, rather than total stimulation duration, may be a key determinant of clinical response.

The comparable outcomes observed between protocols may be explained by the shared structure of the focused TrP stimulation phase applied in both groups. Each protocol incorporated an identical 5-minute stimulation phase delivered directly over the active TrP at 30 Hz (1-second on/ 3-seconds off) and titrated to a non-painful sensory threshold. According to the integrated TrP hypothesis, persistent motor endplate dysfunction may lead to localized ischaemia, excessive acetylcholine release, and accumulation of nociceptive mediators, thereby sustaining peripheral sensitization and ongoing nociceptive input to the dorsal horn [44]. Previous studies suggest that rPMS may modulate these processes through activation of motor axons, rhythmic muscle contractions that improve local perfusion, and preferential recruitment of large-diameter afferent fibres that facilitate segmental inhibitory pathways and reduce nociceptive transmission [21]. These mechanisms are consistent with the parallel increases in PPT observed in both groups. If peripheral desensitization is achieved primarily during this focused phase, extending the surrounding scanning component may provide limited additional suppression of nociceptive signalling at the sensitized locus. Consequently, the biological distinction between the two treatment protocols may have been relatively modest despite the difference in total treatment duration. The shared trigger-point stimulation phase may therefore have represented the principal therapeutic component of the intervention, potentially limiting the ability of the present study to detect duration-dependent dose-response effects. This interpretation is supported by sham-controlled evidence demonstrating short-term clinical benefits of rPMS in upper trapezius MPS [27], indicating that measurable analgesic and functional improvement can occur following a single-session protocol. Together with our findings, this suggests that therapeutic response may depend more on effective engagement of sensitized TrP tissue than on prolonging the total duration of stimulation exposure. Nonetheless, as central pain modulation was not directly evaluated, the potential contribution of supraspinal mechanisms to duration-dependent effects remains unresolved. Future studies incorporating quantitative sensory testing and neurophysiological assessment are needed to clarify the relative peripheral and central contributions of rPMS dose parameters.

Irrespective of between-protocol differences, both treatment durations were associated with substantial and clinically meaningful improvements across multiple outcome domains, including pain intensity, mechanical sensitivity, and neck-related disability over the four-week treatment period. The magnitude of pain reduction exceeded commonly accepted thresholds for clinically meaningful improvement in chronic neck pain populations [45–47]. Likewise, improvements in PPT exceeded previously reported measurement error thresholds for upper trapezius trigger points [48,49], while reductions in neck-related disability surpassed accepted thresholds for meaningful functional improvement [46,50]. The convergence of subjective pain reduction, objective improvements in mechanical sensitivity, and functional recovery supports the clinical relevance of the observed improvements in individuals with upper trapezius MPS.

#### Implications for research.

The present findings suggest that extending total stimulation duration beyond a focused trigger-point stimulation phase may not necessarily enhance short-term clinical outcomes in upper trapezius MPS. This observation raises the possibility that effective engagement of sensitized trigger-point tissue, rather than total stimulation duration alone, may be a key determinant of therapeutic response. However, because central pain modulation was not directly evaluated, the relative contributions of peripheral and supraspinal mechanisms remain uncertain. Future studies incorporating quantitative sensory testing, neurophysiological assessment, and longer-term follow-up are needed to clarify the mechanisms underlying rPMS-induced analgesia and to determine whether duration-dependent effects emerge under alternative stimulation parameters, including frequency, intensity, pulse number, and coil positioning.

#### Implications for clinical practice.

In the broader context of non-invasive, nonpharmacological therapies for chronic pain [51], the findings of this study suggest that extending rPMS session duration may not proportionally enhance clinical outcomes. The absence of duration-dependent differences indicates that rPMS efficacy may depend more on adequate proprioceptive afferent recruitment and cumulative pulse delivery than on total stimulation duration, while optimal stimulation parameters remain incompletely standardized across clinical applications [21]. From a clinical perspective, shorter rPMS protocols targeting the TrP site may therefore achieve outcomes comparable to longer sessions while minimizing unnecessary stimulation exposure. In addition, rPMS offers practical advantages over conventional physical modalities because magnetic stimulation penetrates deeper tissues without requiring skin preparation or patient disrobing, potentially supporting a more time-efficient clinical workflow [21]. Collectively, shorter, focused stimulation protocols may reduce patient fatigue, support treatment adherence, allow therapists to accommodate more patients within routine schedules, and enhance the pragmatic feasibility of rPMS in MSK rehabilitation practice.

#### Strengths and limitations.

This study employed a randomized trial design with assessor blinding, enhancing internal validity and minimizing detection bias. Standardized outcome measures captured both subjective domains (VAS and NDI) and objective mechanical sensitivity (PPT), enabling a multidimensional evaluation of treatment effects. The direct comparison of two active rPMS protocols allowed stimulation duration to be evaluated while other stimulation parameters were held constant. In addition, the use of intention-to-treat analysis with linear mixed-effects modelling and multiple imputation strengthened the robustness of longitudinal inference despite incomplete follow-up.

Several limitations should be acknowledged. First, a sham-controlled group was not included. Although a recent randomized sham-controlled crossover trial demonstrated short-term superiority of rPMS over sham stimulation in neck MPS [27], the present study was designed to compare two active treatment durations under pragmatic rehabilitation conditions. Because both groups received active rPMS in combination with standardized stretching exercises, the independent contribution of rPMS to the observed clinical improvements could not be determined. Consequently, the specific effects of rPMS cannot be distinguished from the effects of stretching exercises or potential contextual and placebo-related influences. Although outcome assessors remained blinded, participant blinding was not feasible because treatment duration differed between protocols, introducing the possibility of expectation-related bias. However, the inclusion of both subjective (VAS and NDI) and objective (PPT) outcomes may partially mitigate this concern. Second, follow-up was limited to 4 weeks; therefore, the durability of treatment effects beyond one month remains uncertain despite favourable short-term outcomes [28,29]. Third, the study included a specific patient population and stimulation protocol at a single rehabilitation centre, which may limit the generalizability of the findings to other patient populations, musculoskeletal conditions, healthcare settings or alternative stimulation protocols. Finally, the study was not designed or powered as an equivalence or noninferiority trial and did not prespecify an equivalence or noninferiority margin. Therefore, the absence of significant between-group differences should not be interpreted as formal evidence of equivalence between treatment durations [52].

## Conclusions

Both treatment durations were associated with significant short-term improvements in pain intensity, pressure pain threshold, and neck-related disability in individuals with upper trapezius MPS. No significant between-protocol differences were detected within the present sample and follow-up period. These findings suggest that a shorter 10-minute rPMS protocol may be a practical treatment option in clinical rehabilitation settings. Future sham-controlled studies with longer follow-up are warranted to determine the specific therapeutic contribution of rPMS, establish optimal stimulation parameters, and evaluate the durability of treatment effects.

## Supporting information

S1 File CONSORT 2025 Checklist. This file contains the completed CONSORT checklist used to ensure transparent reporting of the randomized clinical trial.
(DOCX)

S2 File Thai Research Proposal. This document outlines the original protocol approved by the institutional ethics committee, including study design, intervention procedures, and outcome measures.
(DOCX)

S3 File The study protocol. This file contains the study protocol registered with the Thai Clinical Trials Registry (Ref: TCTR20250929011; https://www.thaiclinicaltrials.org/show/TCTR20250929011).
(PDF)

## Abbreviations

ANCOVA: analysis of covariance
BMI: body mass index
CI: confidence interval
ICC: intraclass correlation coefficient
IQR: interquartile range
ITT: intention-to-treat
LMMs: linear mixed-effects models
MAR: missing at random
MCID: minimal clinically important difference
MICE: multiple imputation by chained equations
MSK: musculoskeletal
MPS: myofascial pain syndrome
NDI: Neck Disability Index
NDI-TH: Thai version of the Neck Disability Index
PPT: pressure pain threshold
REML: restricted maximum likelihood
rPMS: repetitive peripheral magnetic stimulation
SD: standard deviation
TrP: myofascial trigger point
VAS: Visual Analog Scale
