# Supplementary material for: Comparison of two repetitive peripheral magnetic stimulation protocols for upper trapezius myofascial pain syndrome: A randomized trial
Source: PLoS One. 2026 Oct 8;21(10):e0360355. doi: 10.1371/journal.pone.0360355 (PMC13648980; doi:10.1371/journal.pone.0360355)
Supplement: Supplementary file 1 [file pone.0360355.s003.pdf]

TCTR ID : TCTR20250929011

OTHER ID :

Overall Recruitment Status : Pending (Not yet recruiting)

Prospective registration
This protocol was registered before enrollment of the first participant.

Tracking Information

First Submitted Date : 22 September 2025
First Posted Date : 29 September 2025
Last Update Posted Date : 24 September 2025

Title

Public Title : Effectiveness of Two Repetitive Peripheral Magnetic Stimulation Protocols in Managing Upper Trapezius Myofascial Pain: A Single-Blinded Randomized Controlled Trial

Acronym : No Data

Scientific Title : Effectiveness of Two Repetitive Peripheral Magnetic Stimulation Protocols in Managing Upper Trapezius Myofascial Pain: A Single-Blinded Randomized Controlled Trial

Sponsor ID/ IRB ID/ EC ID : HYH EC 061-68-01

Registration Site : Thai Clinical Trials Registry

URL : https://www.thaiclinicaltrials.org/show/TCTR20250929011

Secondary ID : No Secondary ID

Ethics Review

1. Board Approval : Submitted, approved

Approval Number : HYH EC 061-68-01

Date of Approval : 14 August 2025

Board Name : The Ethic Committee for Research in Human Subject Hatyai Hospital

Board Affiliation : Hatyai Hospital

Board Contact : Business Phone : 074273270 Ext. No Data

Business Email : Ethichatyai@gmail.com

Business Address : 182 Rattakarn Road Hatyai District Songkla Province Thailand 90110

Sponsor

Source(s) of Monetary or Material Supports : N/A

Study Primary Sponsor : N/A

Responsible Party : Name/Official Title : Thitirut Jongutchariya

Organization : Department of Rehabilitation Medicine Hatyai Hospital

Phone : 074273100 Ext. 8242

Email : t_jong@hotmail.com

Study Secondary Sponsor : No Study Secondary Sponsor

Protocol Synopsis

Protocol Synopsis : Objectives
Primary Objective
To compare the effectiveness of two rPMS protocols on pain reduction in upper trapezius myofascial pain syndrome across three time points and between groups.
Secondary Objective
To compare the effectiveness of two rPMS protocols on pressure pain threshold and neck disability across three time points and between groups.
Methods
Prospective, randomized controlled trial with assessor blinding to compare two repetitive peripheral magnetic stimulation (rPMS) protocols in patients with upper trapezius myofascial pain syndrome. Participants receive 1 session per week for 4 weeks. Experimental Group (Protocol A): Phase1 perimuscular 3-30Hz, 20% max output increased 5% until non-painful, 10min; Phase2 trigger point 30Hz, 2% increasing, 1s pulse/3s pause, 5min. Total 15min. Control Group (Protocol B): same, Phase1 5min, Phase2 5min. Total 10min. Outcomes assessed across 3 time points and between groups.

URL not available

This document was created on 19 March 2026 at 00:58 (GMT+7).

Health Conditions

Health Condition(s) or Problem(s) Studied : Patients with upper trapezius myofascial pain syndrome

Keywords : rPMS, upper trapezius myofascial pain syndrome, Visual Analog Scale , Pressure Pain Threshold, Neck Disability Index

Eligibility

Inclusion Criteria :
1. Age between 18 and 55 years
2. Patients diagnosed by a physician with myofascial pain syndrome at the neck shoulder region presenting with an active myofascial trigger point according to Simons criteria
3. Patients with upper trapezius muscle pain lasting three months or longer
4. Patients with a Visual Analog Scale pain score of 4 or higher
5. Patients who understand Thai and are able to read and write and complete questionnaires and comply with follow up
6. Patients who provide informed consent to participate in the study

Gender : Both

Age Limit : Minimum : 18 Years Maximum : 55 Years

Exclusion Criteria :
1. Patients with chronic cervical spondylosis cervical spondylolisthesis cervical deformities or scoliosis or C1 C2 dislocation and patients with neurological deficits such as radiculopathy or myelopathy or with abnormal upper motor neuron signs
2. Patients with neurological or neuromuscular diseases such as central nervous system disorders
3. Patients with cervical abnormalities including rheumatoid arthritis or other inflammatory arthritis of the cervical spine such as ankylosing spondylitis or systemic lupus erythematosus
4. Patients with cervical trauma or history of cervical surgery
5. Patients with uncontrolled comorbidities including unstable cardiovascular disease uncontrolled hypertension with blood pressure greater than or equal to 160 over 110 mmHg or uncontrolled diabetes with blood glucose less than 60 mg per dl or greater than or equal to 250 mg per dl
6. Pregnant or breastfeeding women
7. Patients with fibromyalgia
8. Patients with a history of surgery at the neck or shoulder that may affect treatment outcomes
9. Patients with implanted electronic devices or metallic implants such as a cardiac pacemaker or cerebral aneurysm clips
10. Patients with cancer or tumors at the cervical region
11. Patients who received PMS treatment within the past three months
12. Patients with adverse reactions to nonsteroidal anti inflammatory drugs

Accept Healthy Volunteers : No

Status

Overall Recruitment Status : Pending (Not yet recruiting)

Key Trial Dates	Study Start Date (First enrollment) : 13 October 2025	Indicate Type : Anticipated
	Completion Date (Last subject, Last visit) : 13 August 2026	Indicate Type : Anticipated
	Study Completion Date : 13 August 2026	Indicate Type : Anticipated

Design

Study Type : Interventional

Primary Purpose : Treatment

Study Phase : Phase 4

Intervention Model : Parallel

Number of Arms : 2

Masking : Masked Masked Role : Allocation concealment, Subject, Outcome Assessor,

Allocation : Randomized

Control : Dose Comparison

Study Endpoint Classification : Efficacy Study

Sample size

Planned sample size : 66

Intervantion Arm 1

Intervention name : Experimental Group Protocol A Progressive-intensity and Trigger Point

This document was created on 19 March 2026 at 00:58 (GMT+7).

Intervention Type : Active Comparator

Intervention Classification : Device

Intervention Description : The intervention uses a Peripheral Magnetic Stimulation device REMED Rehabilitation Medical Company Salus Talent Pro three Tesla two channel one to one thousand Hertz hand held transducer with circular coil stimulation. Phase 1 involves stimulation around the upper trapezius muscle and fascia at a frequency of three to thirty Hertz starting at twenty percent of maximum intensity and increasing by five percent increments until perceived without pain, lasting ten minutes. Phase 2 involves direct stimulation at the trigger point at thirty Hertz starting at two percent and increasing until perceived without pain, with a pulse pattern of one second on and three seconds off, lasting five minutes. The total duration of both phases is fifteen minutes. Treatments are administered once per week for four weeks.

Intervantion Arm 2

Intervention name : Control Group Protocol B Progressive-intensity and Trigger Point

Intervention Type : Active Comparator

Intervention Classification : Device

Intervention Description : The control group uses a Peripheral Magnetic Stimulation device REMED Rehabilitation Medical Company Salus Talent Pro three Tesla two channel one to one thousand Hertz hand held transducer with circular coil stimulation. Phase 1 consists of stimulation around the upper trapezius muscle and fascia at a frequency of three to thirty Hertz starting at twenty percent of maximum intensity and increasing by five percent increments until perceived without pain, lasting five minutes. Phase 2 consists of direct stimulation at the trigger point at thirty Hertz starting at two percent and increasing until perceived without pain with a pulse pattern of one second on and three seconds off, lasting five minutes. The total session duration is ten minutes. Treatments are administered once per week for four weeks.

Outcome

Primary Outcome

1. Outcome Name : Visual Analog Scale

Metric / Method of measurement : The Visual Analog Scale VAS is a tool for assessing patient pain intensity using a straight line ranging from 0 no pain to 10 worst possible pain.

Time point : 0, 2, and 4 weeks

Secondary Outcome

1. Outcome Name : Pressure Pain Threshold

Metric / Method of measurement : PPT assesses the maximum pressure a patient can tolerate at a specific body point. The value is recorded in kilograms or Newtons, with higher values indicating greater pain tolerance.

Time point : 0, 2, and 4 weeks

Location

Section A : Central Contact

Central Contact	First Name : Thitirut	Middle Name :	Last Name : Jongutchariya
	Degree : Board Certified in Physical Medicine and Rehabilitation	Phone : 074273100 Ext. : 8242	Email : t_jong@hotmail.com
Central Contact Backup	First Name : Surupa	Middle Name :	Lastname : Hangsaphruek
	Degree : Board Certified in Physical Medicine and Rehabilitation	Phone : 074273100 Ext. : 8242	Email : surapa.hsp@gmail.com

Section B Facility Information and Contact

1.	Site Name : Hatyai Hospital		
	City : Hatyai	State/Province : Songkhla	Postal Code : 90110
	Country : Thailand	Recruitment Status : Pending (Not yet recruiting)	
**Facility Contact**	First Name : Thitirut	Middle Name :	Last Name : Jongutchariya
	Degree : Board Certified in Physical Medicine and Rehabilitation	Phone : 074273100 Ext. : 8242	Email : t_jong@hotmail.com
**Facility Contact Backup**	First Name : Surupa	Middle Name :	Last Name : Hangsaphruek
	Degree : Board Certified in Physical Medicine and Rehabilitation	Phone : 074273100 Ext. : 8242	Email : surapa.hsp@gmail.com
**Investigator Name**	First Name : Thitirut	Middle Name :	Last Name : Jongutchariya
	Degree : Board Certified in Physical Medicine and Rehabilitation	Role : Principal Investigator	

Section C : Contact for Public Queries (Responsible Person)

First Name : Thitirut | Middle Name : | Last Name : Jongutchariya

Degree : Board Certified in Physical Medicine and Rehabilitation | Phone : 074273100 Ext. : 8242 | Email : t_jong@hotmail.com

Postal Address : 48/2 Hoymook Road

State/Province : Hatyai/Songkhla | Postal Code : 90110

Country : Thailand | Official Role : Study Principal Investigator

Organization Affiliation : Hatyai Hospital

Section D : Contact for Scientific Queries (Responsible Person)

First Name : Thitirut | Middle Name : | Last Name : Jongutchariya

Degree : Board Certified in Physical Medicine and Rehabilitation | Phone : 074273100 Ext. : 8242 | Email : t_jong@hotmail.com

Postal Address : 48/2 Hoymook Road

State/Province : Hatyai/Songkhla | Postal Code : 90110

Country : Thailand | Official Role : Study Principal Investigator

Organization Affiliation : Hatyai Hospital

Deidentified Individual Participant-level Data Sharing

Plan to share IPD : No

Reason : Fear inappropriate use of data

Publication from this study

MEDLINE Identifier : No Data

URL link to full text publication : No Data

This document was created on 19 March 2026 at 00:58 (GMT+7).
