# Supplementary material for: Comparison of two repetitive peripheral magnetic stimulation protocols for upper trapezius myofascial pain syndrome: A randomized trial
Source: PLoS One. 2026 Oct 8;21(10):e0360355. doi: 10.1371/journal.pone.0360355 (PMC13648980; doi:10.1371/journal.pone.0360355)
Supplement: Supplementary file 2 [file pone.0360355.s001.docx]

**CONSORT 2025 Checklist for Randomized Trial Reporting**

**Manuscript Title**: Effectiveness of Two Repetitive Peripheral Magnetic Stimulation Protocols for Upper Trapezius Myofascial Pain Syndrome: A Randomized Controlled Trial

| **Section/Topic** | **No.** | **CONSORT 2025 checklist item description** | **Reported in Manuscript (on section/page no.** |
| --- | --- | --- | --- |
| **Title and Abstract** |  |  |  |
| Title and structured abstract | 1a | Identification as a randomized trial in the title | Title page (page 1) |
|  | 1b | Structured summary of the trial design, methods, results, conclusions | Abstract (page 1-2, lines 28-68) |
| **Open Science** |  |  |  |
| Trial registration | 2 | Name of trial registry, identifying number (with URL) and date of registration | Abstract “Trial Registration” (page 2, ~ lines 67-69)  Methods – Study Design (page 5, ~ lines 145-148) |
| Protocol and statistical analysis plan | 3 | Where the trial protocol and statistical analysis plan can be accessed | Methods – Study Design – Full trial protocol available via Thai Clinical Trials Registry (TCTR20250929011). Methods – Study Design (page 5, ~ lines 148)  Methods – Statistical Analysis (page 9, ~ lines 274-291) |
| Data sharing | 4 | Where and how the individual de-identified participant data (including data dictionary), statistical code and any other materials can be accessed | Data Availability section (page 19, lines 540-541) |
| Funding and conflicts of interest | 5a | Sources of funding and other support (e.g., supply of drugs), and role of funders in the design, conduct, analysis and reporting of the trial | Funding section (page 19, lines 538-539) |
|  | 5b | Financial and other conflicts of interest of the manuscript authors | Conflicts of Interest section (page 19, lines 555-556) |
| **Introduction** |  |  |  |
| Background and rationale | 6 | Scientific background and rationale | Introduction (page 3-4, lines 70-138) |
| Objectives | 7 | Specific objectives related to benefits and harms | Final paragraph of Introduction (page 4, lines 136-138) |
| **Methods** |  |  |  |
| Patient and public involvement | 8 | Details of patient or public involvement in the design, conduct and reporting of the trial | Methods – Study Design (page 5, lines 152-153) |
| Trial design | 9 | Description of trial design including type of trial (e.g., parallel group, crossover), allocation ratio, and framework (e.g., superiority, equivalence, non-inferiority, exploratory) | Methods – Study Design (page 5-6)  Lines 142: Parallel-group, single-assessor–blinded randomized controlled trial with a 1:1 allocation ratio (superiority framework). |
| Changes to trial protocol | 10 | Important changes to the trial after it commenced including any outcomes or analyses that were not prespecified, with reason | Not applicable (no changes) |
| Trial setting | 11 | Settings (e.g., community, hospital) and locations (e.g., countries, sites) where the trial was conducted | Methods – Study Design (page 5, lines 142-143) |
| Eligibility criteria | 12a | Eligibility criteria for participants | Methods – Participants (page 6, lines 165-179) |
|  | 12b | If applicable, eligibility criteria for sites and for individuals delivering the interventions (e.g., surgeons, physiotherapists) | Methods – Participants / Interventions (page 6, lines 166-167) |
| Intervention and comparator | 13 | Intervention and comparator with sufficient details to allow replication. If relevant, where additional materials describing the intervention and comparator (e.g., intervention manual) can be accessed | Methods – Therapeutic Interventions & rPMS (page 8, lines 236-261) |
| Outcomes | 14 | Pre-specified primary and secondary outcomes, including the specific measurement variable (e.g., systolic blood pressure), analysis metric (e.g., change from baseline, final value, time to event), method of aggregation (e.g., median, proportion), and time point for each outcome | Methods – VAS , PPT, and NDI measurements (page 7-8, lines 208-236) |
| Harms | 15 | How harms were defined and assessed (e.g., systematically, non-systematically) | Methods –  Withdrawal criteria (page 6, lines 180-181)  Rescue Medication (page 9, lines 263-267)  Results – Adverse events statement (page 10, lines 305-306) |
| Sample size | 16a | How sample size was determined, including all assumptions supporting the sample size calculation | Methods – Sample size estimation (page 5-6, lines 154-164) |
|  | 16b | Explanation of any interim analyses and stopping guidelines | Not applicable (no interim analysis) |
| Randomization: |  |  |  |
| Sequence generation | 17a | Who generated the random allocation sequence and the method used | Methods – Randomization and Allocation Concealment (page 6, lines 182-191) |
|  | 17b | Type of randomisation and details of any restriction (e.g., stratification, blocking and block size) | Methods – Randomization and Allocation Concealment (page 6, lines 182-191) |
| Allocation concealment mechanism | 18 | Mechanism used to implement the random allocation sequence (e.g., central computer/telephone; sequentially numbered, opaque, sealed containers), describing any steps to conceal the sequence until interventions were assigned | Methods – Randomization and Allocation Concealment (page 6, lines 182-191) |
| Implementation | 19 | Whether the personnel who enrolled and those who assigned participants to the interventions had access to the random allocation sequence | Methods – Randomization section (page 6, lines 182-191) |
| Blinding | 20a | Who was blinded after assignment to interventions (e.g., participants, care providers, outcome assessors, data analysts) | Methods – Study Design (page 5, lines 142) |
|  | 20b | If blinded, how blinding was achieved and description of the similarity of interventions | Methods – Study Design (page 6, lines 182-191) |
| Statistics methods | 21a | Statistical methods used to compare groups for primary and secondary outcomes, including harms | Methods – Statistical Analysis (page 9, ~ lines 274-291) |
|  | 21b | Definition of who is included in each analysis (e.g., all randomised participants), and in which group | Methods – Statistical Analysis (page 9, ~ lines 274-291) |
|  | 21c | How missing data were handled in the analysis | Methods – Statistical Analysis (page 9, ~ lines 274-291) |
|  | 21d | Methods for any additional analyses (e.g., subgroup and sensitivity analyses), distinguishing prespecified from post-hoc | Methods – Statistical Analysis (page 9, ~ lines 274-291) |
| **Results** |  |  |  |
| Participant flow, including flow diagram | 22a | For each group, the numbers of participants who were randomly assigned, received intended intervention, and were analysed for the primary outcome | Results – Participant Flow (page 10, ~ lines 295-306) / Figure 1 (page 12) |
|  | 22b | For each group, losses and exclusions after randomisation, together with reasons | Results – Participant Flow (page 10, ~ lines 295-306) / Figure 1 (page 12) |
| Recruitment | 23a | Dates defining the periods of recruitment and follow-up for outcomes of benefits and harms | Methods  - Study Design (page 5, ~ lines 144-155)  - Data Collection (page 9, ~ lines 268-273) |
|  | 23b | If relevant, why the trial ended or was stopped | Not applicable (completed as planned) |
| Intervention and comparator delivery | 24a | Intervention and comparator as they were actually administered (e.g., where appropriate, who delivered the intervention/comparator, how participants adhered, whether they were delivered as intended [fidelity]) | Methods – Therapeutic Interventions (page 8, lines 236-261) |
|  | 24b | Concomitant care received during the trial for each group | Methods – Standard Treatment (page 8, lines 240-244)/ Rescue Medication (page 9, lines 263-267) |
| Baseline data | 25 | A table showing baseline demographic and clinical characteristics for each group | Table 1 (page 13) |
| Numbers analysed,  outcomes and estimation | 26 | For each primary and secondary outcome, by group:   - the number of participants included in the analysis - the number of participants with available data at the outcome time point - result for each group, and the estimated effect size and its precision (such as 95% confidence interval)   for binary outcomes, presentation of both absolute and relative effect size | Results – Postintervention outcomes (page 10-11, lines 318-354) + Tables 2–3 (page 14) |
| Harms | 27 | All harms or unintended events in each group | Results – Participant flow (page 10, lines 297-305)/Figure 1 (page 12) and adverse events (page 10, lines 305-306) |
| Ancillary analyses | 28 | Any other analyses performed, including subgroup and sensitivity analyses, distinguishing pre-specified from post-hoc | Method – sensitivity analysis using multiple imputation (page 9, lines ~ 288-290)  Results – page 10 |
| **Discussion** |  |  |  |
| Interpretation | 29 | Interpretation consistent with results, balancing benefits and harms, and considering other relevant evidence | Discussion section (page 15-17, lines 422-479) |
| Limitations | 30 | Trial limitations, addressing sources of potential bias, imprecision, generalisability, and, if relevant, multiplicity of analyses | Limitations subsection (page 18, lines 509-521) |
