# Supplementary material for: Comparison of two repetitive peripheral magnetic stimulation protocols for upper trapezius myofascial pain syndrome: A randomized trial
Source: PLoS One. 2026 Oct 8;21(10):e0360355. doi: 10.1371/journal.pone.0360355 (PMC13648980; doi:10.1371/journal.pone.0360355)
Supplement: Supplementary file 3 [file pone.0360355.s002.docx]

**โครงร่างวิจัย**

**1. ชื่อโครงการ**

**ชื่อภาษาไทย**	การศึกษาประสิทธิผลของเครื่องกระตุ้นแม่เหล็กไฟฟ้าส่วนปลายแบบซ้ำในสองรูปแบบการรักษา ในผู้ป่วยกลุ่มอาการปวดกล้ามเนื้อและพังผืดบริเวณทราพีเซียสส่วนบน: การทดลองแบบสุ่มมีกลุ่มควบคุมและปกปิดฝ่ายเดียว

**ชื่อภาษาอังกฤษ**	Effectiveness of Two Repetitive Peripheral Magnetic Stimulation Protocols in Managing Upper Trapezius Myofascial Pain: A Single-Blinded Randomized Controlled Trial

**2. ชื่อผู้วิจัยหลัก**

**ชื่อ-สกุล	~~นาย~~/~~นาง~~/นางสาว** ฐิติรัตน์ จงอัจฉริย

**~~Mr~~./~~Mrs./~~Miss**	Thitirut Jongutchariya

**ตำแหน่ง**	นายแพทย์ชำนาญการพิเศษ

**หน่วยงาน**	โร**ง**พยาบาลหาดใหญ่

**โทรศัพท์ที่ทำงาน** 074-273100 ต่อ 8241 ถึง 8242

**โทรศัพท์มือถือ**	093-3551538

**E-mail**	t_jong@hotmail.com

**3. ชื่อผู้วิจัยร่วม/ผู้วิจัยหลักร่วม**

**3.1 ชื่อ-สกุล	~~นาย~~/นาง/~~นางสาว~~** สุรภา หังสพฤกษ์

**~~Mr~~./Mrs./~~Miss~~**	Surapa Hangsaphruek

**ตำแหน่ง**	นายแพทย์ชำนาญการพิเศษ

**หน่วยงาน**	โร**ง**พยาบาลหาดใหญ่

**โทรศัพท์ที่ทำงาน** 074-273100 ต่อ 8241 ถึง 8242	**โทรศัพท์มือถือ**	063-2659291

**E-mail**	surapa.hsp@gmail.com

**3.2 ชื่อ-สกุล	นาย/~~นาง~~/~~นางสาว~~** ทวีศักดิ์ วงศ์กีรติเมธาวี

**Mr./~~Mrs.~~/~~Miss~~**	Thawisak Wongkiratimethawi

**ตำแหน่ง**	นักกายภาพบำบัดปฏิบัติการ

**หน่วยงาน**	โร**ง**พยาบาลหาดใหญ่

**โทรศัพท์ที่ทำงาน** 074-273100 ต่อ 8201

**โทรศัพท์มือถือ**	097-3456939

**E-mail**	mm_1800@hotmail.com

**3.3 ชื่อ-สกุล	~~นาย~~/~~นาง~~/นางสาว** นางสาวจิตติมา แสงสุวรรณ

**~~Mr./Mrs.~~/Miss**	Jittima Saengsuwan

**ตำแหน่ง**	รองศาสตราจารย์

**หน่วยงาน**	ภาควิชาเวชศาสตร์ฟื้นฟู คณะแพทยศาสตร์ มหาวิทยาลัยขอนแก่น

**โทรศัพท์ที่ทำงาน** 043-3664123

**โทรศัพท์มือถือ**	081-6619622

E-mail	sjittima@kku.ac.th

**4. งบประมาณและแหล่งทุนสนับสนุน**

⭘ เอกชน

⭘ ทุนอุดหนุนวิจัยจากโรงพยาบาลหาดใหญ่

⭘ ทุนส่วนตัว

**5. ความสำคัญและที่มาของปัญหาที่ทำการวิจัย**

กลุ่มอาการปวดกล้ามเนื้อและพังผืด (Myofascial Pain Syndrome: MPS) เป็นภาวะความเจ็บปวดที่พบได้บ่อยในประชากรทั่วไป โดยเฉพาะในกลุ่มวัยทำงาน และผู้ที่มีพฤติกรรมการใช้งานกล้ามเนื้ออย่างต่อเนื่องหรือซ้ำซาก ลักษณะเฉพาะของภาวะนี้ คือ การเกิด "จุดกดเจ็บ" ภายในกล้ามเนื้อลายหรือเนื้อเยื่อพังผืด (Myofascial Trigger Points: MTrPs) ซึ่งเมื่อถูกกระตุ้นทำให้เกิดอาการปวดเฉพาะที่หรือปวดร้าวไปยังบริเวณอื่นของร่างกาย^1^ อาการดังกล่าวส่งผลกระทบต่อคุณภาพชีวิตของผู้ป่วยในหลายด้าน ทั้งในมิติของการทำงาน การเคลื่อนไหว และสุขภาพจิต โดยกล้ามเนื้อทราพีเซียสส่วนบน (Upper trapezius muscle) เป็นกล้ามเนื้อที่มีหน้าที่สำคัญในการเคลื่อนไหวของคอ ไหล่ และสะบัก และเป็นบริเวณที่พบจุดกดเจ็บได้บ่อยที่สุดจากกล้ามเนื้อทั้งหมดในร่างกาย จากข้อมูลทางคลินิก พบว่า ผู้ป่วยจำนวนมากที่มาด้วยอาการปวดคอและไหล่มักมีจุดกดเจ็บในกล้ามเนื้อนี้ ซึ่งหากไม่ได้รับการรักษาอย่างเหมาะสม อาการปวดอาจกลายเป็นเรื้อรังและนำไปสู่การลดระดับความสามารถในการใช้งานกล้ามเนื้อในคอชีวิตประจำวัน^2,3^ แนวทางการรักษากลุ่มอาการปวด MPS มีหลากหลายวิธี ตั้งแต่ การบริหารกล้ามเนื้อโดยการยืดเหยียดกล้ามเนื้อ การเพิ่มความแข็งแรงของกล้ามเนื้อบริเวณรอบบ่าและคอ การใช้ยาแก้ปวด การฉีดยาเข้ากล้ามเนื้อ การนวดบำบัด การลงเข็มแห้ง (Dry needling: DN) การฝังเข็ม (Acupuncture) และการรักษาด้วยเครื่องมือทางกายภาพต่าง ๆ (Physical modalities) ที่นิยมและมีการศึกษาอย่างแพร่หลาย ได้แก่ การรักษาด้วยกระแสไฟฟ้า (Electrotherapy) เช่น การกระตุ้นเส้นประสาทรับความรู้สึกผ่านผิวหนังด้วยกระแสไฟฟ้า (Transcutaneous Electrical Nerve Stimulation: TENS) การรักษาด้วยความร้อนจากคลื่นเสียงอัลตราซาวด์ (Ultrasound therapy) แสงเลเซอร์ (Laser therapy) และด้วยคลื่นกระแทก (Shockwave therapy) เป็นต้น ตลอดจนรวมถึงการให้ความรู้การปรับท่าทางคอที่เหมาะสมกับการยศาสตร์ในขณะทำงาน เป็นต้น^4^

ปัจจุบันมีการศึกษามากขึ้นเกี่ยวกับการใช้เทคโนโลยีใหม่ในการรักษาภาวะ MPS โดยเฉพาะการกระตุ้นแม่เหล็กไฟฟ้าส่วนปลายแบบซ้ำ (Repetitive Peripheral Magnetic Stimulation: rPMS) ซึ่งเป็นเครื่องมือที่ช่วยลดอาการปวดและฟื้นฟูการทำงานของระบบประสาท หลักการทำงานของเครื่องมือ คือ ใช้สนามแม่เหล็กพลังงานสูงแบบพัลส์ (pulse magnetic field) เพื่อเหนี่ยวนำกระแสไฟฟ้าเข้าสู่ในเนื้อเยื่ออย่างลึก โดยเมื่อผ่านพัลส์กระแสไฟฟ้าความเข้มสูง (high current pulse) เข้าไปในขดลวด (coil) ทำให้เกิดการเหนี่ยวนำที่เปลี่ยนแปลงอย่างรวดเร็วภายในเวลาอันสั้น (millisecond : msec) เกิดคลื่นแม่เหล็กไฟฟ้าและการเหนี่ยวนำนี้ทำให้เกิดกระแสไฟฟ้าไหลเวียน (eddy current) ลงไปสู่โครงสร้างลึกผ่านเส้นใยประสาทนำเข้าการรับรู้ความรู้สึกของข้อต่อ (proprioceptive afferent) ผ่าน 2 กลไกหลัก คือ กลไกโดยตรงกระตุ้นที่เส้นใยประสาทของการรับความรู้สึกและสั่งการ (sensorimotor nerve fibers) และกลไกโดยอ้อมกระตุ้นที่ตัวรับแรงกล (mechanoreceptors) ในใยกล้ามเนื้อ ซึ่งผลจากการกระตุ้นเส้นประสาทรับความรู้สึกและสั่งการ ทำให้เกิดการกระตุ้นกล้ามเนื้อ เกิดการเปลี่ยนแปลงเฉพาะที่ให้เกิดการหดและคลายตัวซ้ำ ๆ ของกล้ามเนื้อ สลับกันตามความถี่ที่กำหนด ตลอดจนส่งผลให้ระบบประสาทสัมผัสและประสาทสั่งการมีการปรับตัวที่ดีขึ้น^5,6^

ตัวอย่างการอธิบายทางพยาธิสรีระวิทยา คือ เมื่อนำ rPMS มากระตุ้นบริเวณที่มีการผ่านของกระแสประสาทส่วนปลายจะกระตุ้นให้เกิดการทำงานของกล้ามเนื้อและกระตุ้นประสาทรับความรู้สึก (afferent fibers) โดยตรง^7^ และพบว่า rPMS ยังช่วยชะลอการส่งสัญญาณประสาทจากเส้นใยประสาทส่งออกขนาดเล็กและขนาดใหญ่ (small and large efferent nerve fiber) และยับยั้งกระแสประสาทบริเวณ dorsal horn ของไขสันหลัง โดยเฉพาะ lamina I, II และ V มีบทบาทสำคัญในกระบวนการรับความรู้สึกเจ็บปวด (nociception) และการรับรู้ความรู้สึกอื่น ๆ เช่น การสัมผัส หรืออุณหภูมิ มีการเพิ่มการไหลเวียนของเลือด และการที่ผู้ป่วยไม่ต้องสัมผัสหรือใช้ไฟฟ้าโดยตรงที่ผิวหนัง ทำให้รู้สึกผ่อนคลายในขณะที่รักษา^8^ และยังช่วยฟื้นฟูความผิดปกติในระบบประสาท (Neurological dysfunction) ด้วยการเปลี่ยนแปลงการรับรู้ทางระบบประสาท (neuroplasticity) ที่ผิดปกติให้กลับมาดีขึ้น^9^

ในการศึกษาวิจัยทางคลินิกก่อนหน้านี้ การกระตุ้น rPMS โดยความหลากหลายของการศึกษาคลอบคลุมทั้ง การประยุกต์ใช้ ประสิทธิผล และความปลอดภัยของเทคนิค rPMS ได้แก่ การช่วยลดอาการปวดจากหลายสาเหตุ เช่น ลดอาการปวดทั้งจากระบบกระดูกกล้ามเนื้อรวมถึงอาการปวดที่มีสาเหตุจากความผิดปกติของระบบประสาท โดย rPMS สามารถลดอาการปวดหลังทั้งกลุ่มเฉียบพลันและเรื้อรัง^10,11^ ช่วยลดอาการปวดปลายประสาทจากบาดเจ็บของร่างแหเส้นประสาทของแขน (traumatic brachial plexopathy)^12^ เป็นต้น และยังมีการศึกษาถึงการนำ rPMS มาช่วยในการรักษาเพื่อการฟื้นตัวของระบบประสาท เช่น เพิ่มความสามารถในการกลืน และลดการสำลักในผู้ป่วยที่มีอาการกลืนลำบากหลังโรคหลอดเลือดสมอง (poststroke dysphagia)^13^ เป็นต้น เทคนิค rPMS แสดงให้เห็นผลลัพธ์เชิงบวกต่อการบำบัดอาการปวด โดยมีข้อดีหลายประการ ได้แก่ เป็นอุปกรณ์ทางการแพทย์ที่สามารถกระตุ้นผ่านวัสดุหรือเสื้อผ้าโดยไม่ต้องสัมผัสผิวหนังโดยตรง สามารถส่งผ่านพลังงานเข้าไปถึงเนื้อเยื่อลึก ไม่มีรายงานผลข้างเคียงรุนแรง และถือว่าเป็นวิธีการรักษาที่ไม่ก่อให้เกิดความเจ็บปวด หากใช้ความเข้มของการกระตุ้นอย่างเหมาะสม^11^ โดยการที่ไม่ทำให้เกิดอาการปวดที่ผิวหนัง ส่งผลให้ผู้ป่วยรู้สึกสบายและมีความปลอดภัย และข้อควรคำนึงด้านความปลอดภัยที่เกี่ยวข้องกับการเกิดความร้อนและการเกิดแม่เหล็กถาวรนั้นอยู่ในระดับเดียวกับที่พบใน การกระตุ้นด้วยคลื่นแม่เหล็กทางกะโหลกศีรษะแบบทำซ้ำ (repetitive transcranial magnetic stimulation: rTMS)^6^

อย่างไรก็ตามแม้ว่า rPMS จะแสดงผลในการลดอาการปวดและเพิ่มการทำงานของกล้ามเนื้อในหลายงานวิจัยที่ผ่านมา แต่แนวทางมาตรฐานที่ชัดเจนในการตั้งค่าพารามิเตอร์การรักษาและการกระตุ้น rPMS ยังคงเป็นเทคนิคที่มีความหลากหลาย โดยรายละเอียดของการตั้งพารามิเตอร์^6^ ประกอบด้วยความถี่ (Frequency) หน่วย Hz (เฮิรตซ์) ความเข้ม (Intensity) มักตั้งเป็นร้อยละของ output สูงสุดของการกระตุ้น รูปแบบการกระตุ้น (Pattern/Pulse protocol) ระยะเวลาในการกระตุ้น (Duration) ความถี่ของการรักษา (Treatment frequency) ตำแหน่งที่กระตุ้น (Target area) และรูปแบบการเพิ่มความเข้ม (Intensity progression) ซึ่งอาจส่งผลต่อประสิทธิผลของการรักษาได้ ดังนั้น ผู้วิจัยจึงมีความสนใจศึกษาประสิทธิผลของการรักษาด้วย rPMS เปรียบเทียบระหว่าง สองรูปแบบของพารามิเตอร์การกระตุ้น เพื่อหาค่าการรักษาที่เหมาะสมที่สุดในการลดอาการปวด ความทนทานต่อความเจ็บปวดจากแรงกด และ**ดัชนีวัดความบกพร่องความสามารถของคอ**ในผู้ป่วยที่มีภาวะปวดกล้ามเนื้อและพังผืดบริเวณกล้ามเนื้อทราพีเซียสส่วนบน ซึ่งจะเป็นประโยชน์ต่อการประยุกต์ใช้ในทางคลินิกอย่างมีประสิทธิภาพ

**6. วัตถุประสงค์**

**วัตถุประสงค์หลัก**

เพื่อเปรียบเทียบประสิทธิผลของเครื่องกระตุ้นแม่เหล็กไฟฟ้าส่วนปลายแบบซ้ำในสองรูปแบบการรักษา ต่อการลดระดับความปวด ในผู้ป่วยกลุ่มอาการปวดกล้ามเนื้อและพังผืดบริเวณทราพีเซียสส่วนบนในการเปลี่ยนแปลง 3 ช่วงเวลาและระหว่างกลุ่ม

**วัตถุประสงค์รอง**

เพื่อเปรียบเทียบประสิทธิผลของเครื่องกระตุ้นแม่เหล็กไฟฟ้าส่วนปลายแบบซ้ำในสองรูปแบบการรักษา ต่อ ความทนทานต่อความเจ็บปวดจากแรงกด และ**ดัชนีวัดความบกพร่องความสามารถของคอ** ในผู้ป่วยกลุ่มอาการปวดกล้ามเนื้อและพังผืดบริเวณทราพีเซียสส่วนบนในการเปลี่ยนแปลง 3 ช่วงเวลาและระหว่างกลุ่ม

**7. การทบทวนวรรณกรรม**

**ระบาดวิทยา ความชุก และอุบัติการณ์อาการปวดคอและกลุ่มอาการปวดกล้ามเนื้อและพังผืด**

อาการปวดคอ เป็นหนึ่งในอาการทางระบบกล้ามเนื้อและกระดูกที่พบบ่อยในเวชปฏิบัติ โดยมีรายงานว่า ประมาณร้อยละ 70 ของประชากรทั่วไปเคยมีอาการปวดคออย่างน้อยหนึ่งครั้งตลอดช่วงชีวิต อุบัติการณ์ของอาการปวดคอมีแนวโน้มเพิ่มขึ้นตามอายุ และมีความสัมพันธ์กับลักษณะการทำงาน โดยเฉพาะในช่วงวัยทำงาน พบว่า อาการปวดคอมีความชุกสูงที่สุดในกลุ่มอายุระหว่าง 35–49 ปี ซึ่งมักประกอบอาชีพที่ต้องอยู่ในท่าทางเดิมเป็นเวลานานหรือใช้กล้ามเนื้อซ้ำ ๆ^14^ ทั้งนี้การศึกษา Côté และคณะ ในปี 2008^15^ รายงานว่าอายุเฉลี่ยของกลุ่มที่มีอาการปวดคออยู่ที่ 45.8 ± 13.4 ปี โดยประมาณร้อยละ 47.2 เป็นเพศหญิง และครึ่งหนึ่งของกลุ่มตัวอย่างมีงานประจำ แสดงให้เห็นว่าอาการปวดคอมักเกิดขึ้นในประชากรกลุ่มวัยทำงาน ซึ่งอาจเกี่ยวข้องกับภาระงานและท่าทางในการทำงาน นอกจากนี้ยังมีรายงานผู้ป่วยประมาณร้อยละ 30 ที่เคยมีอาการปวดคอจะยังคงมีอาการต่อเนื่องและอาจพัฒนาไปสู่ภาวะปวดคอเรื้อรัง^16^ ซึ่งส่งผลกระทบในหลายมิติ ทั้งคุณภาพชีวิต ประสิทธิภาพในการทำงาน ค่าใช้จ่ายในการรักษาพยาบาล และการสูญเสียโอกาสทางเศรษฐกิจ โดยมีรายงานในประเทศสหรัฐอเมริกาปี 2016 ว่าค่าใช้จ่ายในการรักษาผู้ป่วยกลุ่มนี้มีมูลค่าสูง^17^ สำหรับประทศไทยมีการศึกษาวิจัยเกี่ยวกับ อาการปวดคอ ถือเป็นปัญหาสุขภาพที่พบได้บ่อยในคนไทย โดยเฉพาะในกลุ่มวัยทำงาน ซึ่งมักจะสัมพันธ์กับพฤติกรรมการทำงานและการใช้ชีวิตประจำวัน การศึกษาของ Nainate^18^ เก็บข้อมูลระหว่างเดือนพฤศจิกายน 2562 - มกราคม 2563 ในกลุ่มบุคลากรในบุคลากรที่ใช้คอมพิวเตอร์ในโรงพยาบาลเจริญกรุงประชารักษ์ รายงานความชุกของอาการปวดคอบ่าไหล่ในช่วง 1 ปีที่ผ่านมา สูงถึง ร้อยละ 94.7 แม้ว่าตัวเลขนี้จะสูงกว่าการศึกษาในประเทศไทยที่ผ่านมาของ Punwaree และคณะ^19^ ในปี 2552 ศึกษาในบุคลากรที่ใช้คอมพิวเตอร์ พบว่า ความชุกของอาการปวดคอประมาณ ร้อยละ 65 ทำให้สะท้อนให้เห็นถึงความสำคัญของอาการนี้ในกลุ่มคนวัยทำงานมากที่ใช้คอมพิวเตอร์มากขึ้นในช่วงเกือบ 10 ปีที่ผ่านนมา ซึ่งปัจจัยอื่น ๆ ที่มีผลต่อการปวดต้นคอ ได้แก่ อายุ โดยช่วงอายุที่พบบ่อย คือ 25-50 ปี ซึ่งเป็นกลุ่มวัยทำงาน ตลอดจนอายุที่เพิ่มขึ้น โดยเฉพาะประมาณ 45 ปีขึ้นไป อาการปวดคอมักเกิดจากกระบวนการเสื่อมของหมอนรองกระดูกและข้อต่อระหว่างกระดูกสันหลังพบในเพศหญิงมากกว่าเพศชาย ลักษณะทางการยศาสตร์และพฤติกรรมการทำงาน เช่น การใช้คอมพิวเตอร์เป็นเวลานาน การก้มคอใช้โทรศัพท์มือถือและอุปกรณ์อิเล็กทรอนิกส์ หรือ อิริยาบถในการทำงานที่ไม่เหมาะสม เป็นต้น ความหนักของการทำงาน เช่น ชั่วโมงการทำงานที่มาก เป็นต้น^18-20^ จากการศึกษาวิจัยที่เกี่ยวข้องกับอาการปวดต้นคอ กลุ่มอาการปวด MPS บริเวณกล้ามเนื้อทราพีเซียสส่วนบน คือ หนึ่งในสาเหตุสำคัญที่พบบ่อย โดยลักษณะเด่นของภาวะนี้ คือ การเกิดจุดกดเจ็บ ซึ่งทำให้เกิดอาการปวดเฉพาะที่หรือปวดร้าว^1-3,21^ การศึกษาของ Téllez และคณะ^22^ ในกลุ่มผู้ป่วยที่มีอาการปวดต้นคอเรื้อรังแบบไม่จำเพาะเจาะจง พบว่า ความชุกของจุดกดเจ็บกล้ามเนื้อและพังผืด บริเวณกล้ามเนื้อทราพีเซียสส่วนบน สูงถึงร้อยละ ร้อยละ 93.75 โดยพบจุดกดเจ็บที่มีอาการ (Active trigger point: Active TrP) ทางด้านขวา ร้อยละ 82.1 และด้านซ้าย ร้อยละ 79 นอกจากนี้ ยังพบจุดกดเจ็บที่มีอาการในกล้ามเนื้ออื่น ๆ ด้วย ได้แก่ กล้ามเนื้อเลเวเตอร์ สแคปปูเล (Levator Scapulae) ร้อยละ 82.14 กล้ามเนื้อมัลติฟิได (Multifidi) ร้อยละ 77.68 และกล้ามเนื้อสพลีเนียส เซอร์วิซิส (Splenius Cervicis) ร้อยละ 62.5 นอกจากนี้ สอดคล้องกับการศึกษาวิจัยของ Lam และคณะ^23^ รายงานว่า กลุ่มอาการปวด MPS เป็นสาเหตุของอาการปวดต้นคอและศีรษะเรื้อรังถึง ร้อยละ 54.6 จากข้อมูลข้างต้น แสดงให้เห็นว่ากล้ามเนื้อทราพีเซียสส่วนบนมีบทบาทสำคัญในการเกิดอาการปวดต้นคอ และความชุกของจุดกดเจ็บในกล้ามเนื้อนี้สูงมากในผู้ป่วยที่มีอาการปวดต้นคอเรื้อรัง

**หลักการและกฏเกณฑ์การวินิจฉัย**

กลุ่มอาการปวด MPS มี แนวทางการวินิจฉัย ตามเกณฑ์การวินิจฉัยดั้งเดิมของ Simons และคณะ^1^ ประกอบด้วยประวัติและการตรวจหา TrP คือ 1. ประวัติ มีอาการปวดกล้ามเนื้อ มักจะเป็นบริเวณใดบริเวณหนึ่งของร่างกาย เช่น อาการปวดศีรษะ คอ สะบัก บ่า แขน หลัง หรือ ขา อาจมีบางกครั้งที่ปวดหลายบริเวณพร้อมกัน ขึ้นกับการจำนวนและการกระจายของ TrP 2. การตรวจร่างกายพบ TrP ที่มีลักษณะเฉพาะ คือ เป็นจุดปวดที่ไวต่อการกระตุ้น (hyperirritable spot) อยู่ในกล้ามเนื้อที่มีแถบตึง (taut band) สามารถกระตุ้นให้เกิดอาการปวดร้าว (referred pain) ที่มีแบบแผนเฉพาะจุดได้ โดยใช้แรงกด หรือใช้ปลายเข็มลงไปที่ TrP และคลำได้ปฏิกิริยาการกระตุกของเส้นใยกล้ามเนื้อเฉพาะที่อย่างรวดเร็วและเป็นอัตโนมัติ (local twitch response) โดยเฉพาะอย่างยิ่งในรายที่ TrP อยู่ไม่ลึกมาก หรือ เกณฑ์การวินิจฉัย MPS ฉบับปรับปรุงของ Simon^1,24^ ประกอบด้วยเกณฑ์หลัก (major criteria) 5 ข้อ ได้แก่ 1.) มีอาการปวดเฉพาะที่ (a regional pain) 2.) มีอาการปวดร้าว (a stereotypic zone of referred pain) หรือการเปลี่ยนแปลงของความรู้สึกตรงกับอาการของจุดกดเจ็บ 3.) สามารถคลำ taut band ได้ในกล้ามเนื้อที่เข้าถึงได้ 4.) ใน taut band มีจุดกดเจ็บ ที่สามารถกดเจ็บได้มากที่สุด และ 5.) พิสัยการเคลื่อนไหว (range of motion) ของข้อลดลงสัมพันธ์กับกล้ามเนื้อที่มีจุดกดเจ็บ และเกณฑ์รอง (minor criteria) 3 ข้อ ได้แก่ 1.) สามารถกระตุ้นให้เกิดอาการปวด หรือ ความรู้สึกที่เปลี่ยนแปลงไปได้ด้วยการกดที่จุดกดเจ็บนี้ 2.) สามารถกระตุ้นให้เกิด local twitch response ได้ด้วยการแทงเข็ม หรือ คลึงในแนวตั้งฉากกับ taut band ตรงตำแหน่งที่มีจุดกดเจ็บ และ 3.) การบริหารยืดกล้ามเนื้อ หรือ ฉีดยาที่จุดกดเจ็บทำให้อาการปวดลดลงได้ โดยการวินิจฉัยจะต้องประกอบไปด้วยเกณฑ์หลักทั้ง 5 ข้อ และเกณฑ์รอง อย่างน้อย 1 ข้อ

**แนวทางการรักษากลุ่มอาการปวดกล้ามเนื้อและพังผืด**

หลักสำคัญการรักษา MPS คือ ต้องเข้าใจ MPS เป็นภาวะหรือกลุ่มอาการ จำเป็นต้องมี 1. การรักษาเฉพาะที่ (local treatment) คือ มุ่งเน้นการคลายจุดกดเจ็บ ลดความตึงตัวของกล้ามเนื้อ (symptomatic treatment) และ 2. การรักษาโรคที่ก่อให้เกิด MPS คือ การค้นหาสาเหตุ และแก้ไขปัจจัยที่ทำให้อาการคงอยู่ (correct perpetuating factors) ที่มักจะเป็นสาเหตุ หรือ ปัญหาที่แท้จริง ถือเป็นการักษาเฉพาะโรค (specific treatment) หรือ อาจแบ่งการรักษาออกเป็น 2 กลุ่มหลัก คือ การรักษาแบบใช้ยา (Pharmacological treatment) และ การรักษาแบบไม่ใช้ยา (Non-pharmacological treatment) โดยมีรายละเอียด ดังนี้

**1. การรักษาแบบใช้ยา**

**1.1 ยารับประทาน** ที่ใช้บ่อยในเวชปฏิบัติและนิยมใช้มากที่สุดคือ ยาต้านการอักเสบที่ไม่ใช่สเตียรอยด์ (NSAIDs) เนื่องจากมีผลข้างเคียงในระดับต่ำและสามารถเข้าถึงได้ง่าย อย่างไรก็ตาม หลักฐานสนับสนุนประสิทธิภาพของ NSAIDs และยาคลายกล้ามเนื้อยังมีข้อจำกัด และผลการศึกษาหลายฉบับยังแสดงความไม่แน่ชัดในผลลัพธ์ทางคลินิก ขณะที่ยา NSAIDs แบบทาเฉพาะที่ เช่น diclofenac gel หรือ lidocaine patch มีข้อได้เปรียบในด้านผลข้างเคียงน้อยกว่ายารับประทาน แม้จะมีราคาสูงกว่า^25^ สำหรับยาทาเฉพาะที่อื่น ๆ เช่น lidocaine และ capsaicin cream มีหลักฐานว่าให้ผลดีกว่าการให้ placebo และอาจเปรียบเทียบได้กับการฉีดจุดกดเจ็บในบางกรณี^26^ ดังนั้นการเลือกใช้ยารักษา MPS ควรคำนึงถึงลักษณะอาการร่วมของผู้ป่วย และเลือกใช้ยาโดยพิจารณาความเสี่ยงและผลข้างเคียงที่อาจเกิดขึ้นอย่างรอบคอบ

**1.2 การลงเข็มฉีดยาที่จุดกดเจ็บ (Trigger Point Injection: TPI)**^25 ,27-29^ เป็นหัตถการที่ใช้เข็มฉีดยาเข้าสู่จุดกดเจ็บเพื่อคลายเส้นใยกล้ามเนื้อ ลดการสะสมของของเสีย และเพิ่มการไหลเวียนโลหิต โดยเฉพาะในผู้ป่วยที่ไม่ตอบสนองต่อกายภาพบำบัด มีการใช้เทคนิคหลากหลาย เช่น การเจาะตรง การฉีดแบบกระจาย (fanning) และการใช้ ultrasound หรือ fluoroscopy นำทาง ซึ่งให้ความแม่นยำสูงและลดภาวะแทรกซ้อน ข้อห้ามของการรักษานี้ ได้แก่ การติดเชื้อ ภาวะเลือดออกผิดปกติ และความไม่ร่วมมือของผู้ป่วย

**2. การรักษาแบบไม่ใช้ยา**

**2.1 การออกกำลังกาย** ถือเป็นองค์ประกอบสำคัญในกระบวนการฟื้นฟูและการจัดการความปวดของระบบกล้ามเนื้อและกระดูก โดยมีบทบาทสำคัญในการเพิ่มความยืดหยุ่นของกล้ามเนื้อ ฟื้นฟูสมรรถภาพการทำงาน ปรับปรุงอารมณ์ และลดความรู้สึกเจ็บปวด ประสิทธิภาพของการออกกำลังกายในการรักษา Myofascial Pain Syndrome เป็นที่ยอมรับอย่างกว้างขวาง โปรแกรมการออกกำลังกายที่มีลักษณะค่อยเป็นค่อยไป มีความครอบคลุม และต่อเนื่อง ซึ่งรวมถึงการยืดกล้ามเนื้อ การเสริมความแข็งแรง และการออกกำลังกายแบบแอโรบิก จะก่อให้เกิดประโยชน์ทั้งทางกายภาพและด้านจิตสังคม^30^

**2.2 การลงเข็มแห้ง** เป็นเทคนิคที่ได้รับความนิยมในการแพทย์ตะวันตก โดยมุ่งเป้าหมายการรักษาไปที่การคลายจุดกดเจ็บผ่านการลงเข็มเข้าสู่กล้ามเนื้อโดยตรง เพื่อคลายจุดกดเจ็บ เป็นการรักษากลุ่มอาการปวดกล้ามเนื้อและพังผืดที่มีประสิทธิภาพและได้รับการยอมรับว่าสามารถลดอาการปวดของกลุ่มอาการปวด กล้ามเนื้อและพังผืดได้จริง^31,32^ ผลทางการกลศาสตร์ (mechanical effects) สำหรับการลงเข็มแห้ง ซึ่งเชื่อว่าเป็นผลของการรักษาจุดกดเจ็บ คือ ปลายเข็มไปทำลายปลายเส้นประสาท (mechanical disruption) ถือเป็นการตัดการหลั่ง acetylcholine จากปลายประสาท ปลายเข็มไปทำให้เกิดการบาดเจ็บของใยกล้ามเนื้อ เกิดการหดเกร็งเฉพาะจุดบริเวณจุดกดเจ็บ ทำให้มีการหลั่งโพแทสเซียมออกมาจากเซลล์จนความเข็มข้นสูงพอที่จะสกัดกั้นการส่งกระแสสัญญาณความปวด เป็นต้น ซึ่งกลไกดังกล่าวมีส่วนช่วยลดสารสื่อปวดในบริเวณนั้น และกระตุ้นกลไกการซ่อมแซมของเนื้อเยื่อ^33^

**2.3 การรักษาด้วยเครื่องมือทางกายภาพบำบัด** เป็นที่นิยมและมีการศึกษาอย่างแพร่หลาย แม้ว่ายังไม่มีเครื่องมือกายภาพบำบัด หรือ เทคนิคการบำบัดด้วยมือวิธีใดที่แสดงประสิทธิภาพเหนือกว่าวิธีอื่นอย่างชัดเจน แต่จากการทบทวนวรรณกรรมอย่างเป็นระบบและวิเคราะห์อภิมานล่าสุด^34^ พบว่า เทคนิคคลายพังผืดกล้ามเนื้อ (Myofascial Release: MFR) หรือ เทคนิคการยืดกล้ามเนื้อร่วมกับการหดเกร็งโดยสมัครใจ (Muscle Energy Technique: MET) เป็นวิธีที่มีการนำมาใช้บ่อย การรักษาด้วยกระแสไฟฟ้า เช่น การกระตุ้นเส้นประสาทรับความรู้สึกผ่านผิวหนังด้วยกระแสไฟฟ้า มีประสิทธิภาพในการควบคุมความปวดในระยะสั้น แต่ยังไม่มีหลักฐานยืนยันถึงผลในระยะยาว ขณะที่การรักษาด้วยความร้อนจากคลื่นเสียงอัลตราซาวด์^35^ เป็นวิธีที่มีการใช้กันอย่างแพร่หลาย อย่างไรก็ตาม หลักฐานทางวิชาการเกี่ยวกับประโยชน์ของวิธีนี้ยังไม่ชัดเจนการรักษาด้วยวิธีอื่น ๆ เช่น แสงเลเซอร์และด้วยคลื่นกระแทก อาจให้ผลบรรเทาอาการในระยะสั้นดีกว่ากลุ่มยาหลอก^34^

**2.4 การรักษาอื่น ๆ** เช่น การฝังเข็มแบบดั้งเดิม (Traditional acupuncture) พบว่า มีประโยชน์ โดยเฉพาะเมื่อนำมาใช้ร่วมกับแนวทางการรักษาแบบผสมผสาน (Multimodal approach) แม้ว่าจะมีเพียงไม่กี่การศึกษาเท่านั้นที่แสดงให้เห็นว่า การฝังเข็มมีประสิทธิภาพเหนือกว่าการใช้ยาทางเภสัชกรรม^36^ เทคนิคบำบัดด้านจิตใจและพฤติกรรม (Psychological & Behavioral Approaches)^37^ เทคนิคที่ลดความเครียด เช่น การฝึกสมาธิ โยคะ การบำบัดพฤติกรรมทางปัญญา (Cognitive Behavioral Therapy: CBT) ช่วยลดโทนของกล้ามเนื้อ เพิ่มระดับความทนต่อความเจ็บปวด และส่งเสริมการฟื้นตัว รวมถึงการให้ความรู้ผู้ป่วย ควรส่งเสริมความเข้าใจในโรค MPS นำไปสู่การปรับพฤติกรรม และการหลีกเลี่ยงสิ่งกระตุ้นที่ก่อให้เกิด TrP

แนวทางการักษา MPS ที่มีประสิทธิภาพควรมีลักษณะเป็นการบูรณาการหลากหลายวิธีที่และเหมาะสมกับผู้ป่วยแต่ละราย โดยยังไม่มีวิธีการรักษาใดที่แสดงความชัดเจนและเหนือกว่ากันอย่างมีนัยสำคัญทางสถิติในระยะยาว การวิจัยเพิ่มเติมเพื่อเปรียบเทียบประสิทธิผลของแนวทางต่าง ๆ รวมถึงวิธีใหม่ ยังคงเป็นความท้าทายและน่าสนใจ ปัจจุบันมีการศึกษามากขึ้นเกี่ยวกับการใช้เทคโนโลยีใหม่ในการรักษาภาวะ MPS โดยเฉพาะการใช้เทคนิค rPMS เป็นอุปกรณ์ทางการแพทย์ซึ่งเกิดจากการกระตุ้นด้วยคลื่นแม่เหล็กความแรงสูงโดยการรักษาด้วยแม่เหล็กไฟฟ้านั้นมีมานาน แต่สมัยก่อนคลื่นแม่เหล็กต่ำจึงส่งผลให้การรักษาต้องใช้เวลานานและเห็นผลลัพธ์ไม่ชัดเจน แต่ปัจจุบันมีการปรับให้คลื่นแม่เหล็กนั้นมีคลื่นความแรงที่สูงมากขึ้นจึงทำให้มีประสิทธิภาพในการรักษามากขึ้น^38^ เป็นการักษาที่ไม่รุกล้ำร่างกายผ่านการปรับหรือควบคุมการทำงานของระบบประสาท (a non-invasive neuromodulation) ด้วยวิธีการกระตุ้นทางแม่เหล็กไฟฟ้า หนึ่งในกลไกการออกฤทธิ์ของ rPMS ที่เชื่อกันว่าให้ผล คือ การกระตุ้นกล้ามเนื้อบริเวณที่ปวดในพื้นที่กว้าง ทำให้เสริมการรับรู้ตำแหน่งของร่างกายผ่านทางเส้นประสาทรับความรู้สึก (proprioceptive afferents) และสามารถกระตุ้นการหดตัวของกล้ามเนื้อโดยตรง และกระตุ้นตัวรับความรู้สึกเชิงกล (mechanoreceptors) ภายในใยกล้ามเนื้อโดยทางอ้อม^6^ นอกจากนี้ ยังเชื่อว่า คลื่นแม่เหล็กที่กระตุ้นเส้นประสาทรอบนอกสามารถกระตุ้นเส้นใยประสาทขนาดใหญ่ชนิด A-beta และยับยั้งการนำสัญญาณของเส้นใยประสาทขนาดเล็กชนิด A-delta และ C ซึ่งทำหน้าที่ส่งสัญญาณความเจ็บปวดไปยังสมอง ส่งผลให้ความรู้สึกเจ็บปวดลดลง และกระตุ้นกลไกการยับยั้งความเจ็บปวดที่ลงมาจากระบบประสาทส่วนกลาง (descending inhibitory pathway)^39^ โดยไม่กระตุ้นเส้นประสาทรับความรู้สึกทางผิวหนัง จึงไม่ทำให้เกิดความเจ็บปวดเพิ่มเติมระหว่างการรักษา^8^ จากการทบทวนวรรณกรรม พบว่า มีการศึกษาเกี่ยวกับ rPMS เพื่อบำบัดอาการปวด และอาการทางระบบประสาทต่าง ๆ^10-13^

**งานวิจัยที่ผ่านมาของการรักษาด้วยเครื่องกระตุ้นแม่เหล็กไฟฟ้าส่วนปลายแบบซ้ำ ๆ ที่ช่วยลดอาการปวด**

- การศึกษาของ Kim และคณะในปี ค.ศ. 2021^40^ ที่ศึกษาผลของผลของการกระตุ้นด้วย rPMS ต่อความปวด คะแนน Korean Oswestry Disability Index (KODI) และคุณภาพชีวิตในผู้ป่วยปวดหลังส่วนล่างเรื้อรัง โดยในกลุ่มทดลอง ได้รับการรักษาด้วย rPMS สัปดาห์ละ 1 ครั้ง ครั้งละ 10 นาที ต่อเนื่องเป็นระยะเวลา 4 สัปดาห์ โดยใช้หัวขดลวดทรงกลม (round coil) ในการกระตุ้น ตำแหน่งที่กระตุ้นจะพิจารณาจากจุดกดเจ็บ (trigger point) ที่ไวต่อความรู้สึกมากที่สุดของผู้ป่วย และวางขดลวดในแนวขนานกับพื้นผิวร่างกายเพื่อเพิ่มประสิทธิภาพสูงสุดของการกระตุ้นด้วยสนามแม่เหล็กในบริเวณเป้าหมาย การกระตุ้นแต่ละครั้งใช้เวลา 10 นาที โดยใช้โปรโตคอลการกระตุ้นแบบไม่ต่อเนื่อง คือ กระตุ้นเป็นเวลา 5 วินาที ที่ความถี่ 20 Hz สลับกับช่วงพัก 2 วินาที รวมจำนวนครั้งที่กระตุ้นตลอด 10 นาที เท่ากับ 1,400 ครั้ง เทียบกับกลุ่มควบคุมในกลุ่มควบคุมได้รับการทำกายภาพบำบัดทั่วไปสัปดาห์ละ 1 ครั้ง ครั้งละ 10 นาที เป็นระยะเวลา 4 สัปดาห์ โดยการทำกายภาพบำบัดทั่วไปใช้การรักษาด้วยกระแสไฟฟ้าแบบสัญญาณแทรกสลับ (Interferential Current Therapy: ICT) พบว่า เมื่อเทียบการเปลี่ยนแปลงของคะแนน VAS ระหว่างกลุ่มทดลอง และกลุ่มควบคุม ที่ 4 สัปดาห์ หลังการรักษามีค่าเท่ากับ 2 ± 0.08 และ 4.2 ± 1.14 มีความแตกต่างกันอย่างมีนียสำคัญทางสถิติ (p < 0.001)

- การศึกษาของ Nathinee และคณะ ปี พ.ศ. 2566^41^ เปรียบเทียบประสิทธิผลของการรักษาระหว่างเครื่องกระตุ้นประสาทส่วนปลายด้วยแม่เหล็กไฟฟ้า (rPMS) กับการลงเข็ม (DN) ในผู้ป่วยกลุ่มอาการปวดกล้ามเนื้อและพังผืดคอ พบว่า ศึกษาในอาสาสมัคร จำนวน 44 คน โดยสุ่มเป็น 2 กลุ่ม คือ กลุ่ม rPMS และกลุ่ม DN วัดค่ามัธยฐานระดับคะแนนความเจ็บปวด ในช่วงก่อนการรักษา หลังการรักษาที่ 2 และ 4 สัปดาห์ ผลการศึกษาพบว่า ทั้ง 2 กลุ่ม คือ กลุ่ม rPMS และกลุ่ม DN มีค่ามัธยฐานระดับคะแนน VAS ลดลง อย่างมีนัยสำคัญทางสถิติ หลังการรักษาที่ 2 และ 4 สัปดาห์ แต่เมื่อเทียบการเปลี่ยนแปลงของคะแนน VAS ระหว่างกลุ่ม PMS และกลุ่ม DN พบว่า ค่ามัธยฐานคะแนน VAS ไม่แตกต่างกันอย่างมีนัยสำคัญทางสถิติ

- การศึกษาของ Vearasilp และคณะ ปี พ.ศ.2568^42^ ศึกษาประสิทธิผลของ rPMS ในการบรรเทาอาการปวดกล้ามเนื้อหลังการรักษาด้วยเข็มแห้งในผู้ป่วยกลุ่มอาการปวดกล้ามเนื้อและพังผืดบริเวณกล้ามเนื้อทราพีเซียสส่วนบน: การทดลองทางคลินิกแบบสุ่มและปกปิดสองทาง พบว่า กลุ่มที่ได้รับ rPMS มีค่าความทนทานต่อความเจ็บปวดจากแรงกด (Pressure Pain Threshold: PPT) เพิ่มขึ้นอย่างมีนัยสำคัญเมื่อเปรียบเทียบกับกลุ่มหลอก (P = 0.002) นอกจากนี้ คะแนนระดับความปวดจากแบบประเมิน Numeric Rating Scale (NRS) ยังดีขึ้นอย่างมีนัยสำคัญในกลุ่ม rPMS เมื่อเทียบกับกลุ่มหลอก (P < 0.05) โดยไม่มีรายงานเหตุการณ์ไม่พึงประสงค์รุนแรงใด ๆ ตลอดระยะเวลาการศึกษา

- การศึกษาของ Jiravichitchai และคณะ^43^ ที่กำหนดพารามิเตอร์ของการรักษา โดยการตั้งความถี่ระหว่าง 3 ถึง 30 เฮิรตซ์ โดยเริ่มต้นความเข้มของการกระตุ้นที่ร้อยละ 20 ของกำลังสูงสุดของเครื่อง และเพิ่มขึ้นทีละร้อยละ 5 จนผู้เข้าร่วมรู้สึกถึงการกระตุ้นโดยไม่รู้สึกเจ็บปวด ซึ่งถือว่าอยู่ในระดับต่ำกว่าขีดกระตุ้น (subthreshold stimulation) เป็นระยะเวลา 10 นาที จากนั้นจึงทำการกระตุ้นที่จุดกดเจ็บ (trigger point) โดยใช้ความถี่ 30 เฮิรตซ์ เริ่มต้นที่ความเข้มร้อยละ 2 และจนผู้เข้าร่วมรู้สึกถึงการกระตุ้นโดยไม่รู้สึกเจ็บปวด โดยให้พัลส์ (delivering pulses) เป็นระยะเวลา 1 วินาที สลับกับพัก 3 วินาที ต่อเนื่องเป็นเวลา 5 นาที รวมระยะเวลาการรักษา 15 นาที และมีข้อเด่น ที่กระตุ้น PMS ที่จุดกดเจ็บ ถึง 5 นาที พบว่า คะแนนความปวดจากแบบประเมิน Visual Analog Scale (VAS) ดีขึ้นอย่างมีนัยสำคัญในทั้งสองกลุ่มภายหลังการรักษา 1 สัปดาห์ โดยกลุ่มที่ได้รับการกระตุ้นด้วย rPMS แสดงผลลัพธ์ที่ดีกว่า โดยคะแนนเฉลี่ยของ VAS ในกลุ่ม rPMS หลังการรักษาครั้งที่ 2 และ 3 เท่ากับ 2.5 (SD = 1.6) และ 2.5 (SD = 1.4) ตามลำดับ

ทั้งนี้การรักษาด้วย rPMS ยังไม่มีแนวทางมาตรฐานในการกำหนดค่าพารามิเตอร์ที่เหมาะสม ทั้งการตั้งค่าโหมดการรักษา (Mode of treatment) ความแรงของสนามแม่เหล็ก (Intensity) ระยะเวลาของการรักษา (Treatment duration) และจำนวนการรักษา (Number of Sessions) ด้วยเหตุนี้ทางผู้วิจัยจึงสนใจว่าการศึกษาดังกล่าว หากกำหนดค่าพารามิเตอร์ rPMS 2 รูปแบบ (protocol) ที่ต่างกันในช่วงเวลาของการรักษาทั้งหมด 10 นาที และ 15 นาที ที่เห็นได้ว่ามีประสิทธิภาพในการลดอาการปวด^40,43^ จึงเป็นที่มาของการเพื่อหาค่าระยะเวลาที่ใช้ในการรักษาที่เหมาะสมที่สุดโดยมีเป้าหมายการศึกษาประสิทธิผลของการรักษาด้วยเครื่องกระตุ้นแม่เหล็กไฟฟ้าส่วนปลายแบบซ้ำระหว่าง 2 รูปแบบการรักษาต่อระดับความปวด ความทนทานต่อความเจ็บปวดจากแรงกด และดัชนีวัดความบกพร่องความสามารถของคอ: การทดลองแบบสุ่มมีกลุ่มควบคุมและปกปิดฝ่ายเดียวในผู้ป่วยที่มีภาวะกลุ่มอาการปวดกล้ามเนื้อและพังผืดบริเวณกล้ามเนื้อทราพีเซียสส่วนบน ซึ่งจะเป็นประโยชน์ต่อการประยุกต์ใช้ในทางคลินิกอย่างมีประสิทธิภาพ โดยมีเป้าหมายเพื่อหาแนวทางการรักษาที่มีประสิทธิภาพ ชัดเจน และสอดคล้องกับการปฏิบัติในคลินิกยุคใหม่

**8. วิธีดำเนินการวิจัยและแนวทางการดำเนินงาน**

**8.1 ระเบียบวิธีวิจัย (Material and methods)**

**Study Design**

งานวิจัยนี้เป็นการวิจัยเชิงทดลอง (Experimental Study) ประเภทการทดลองแบบสุ่มควบคุม (Randomized Controlled Trial: RCT) ซึ่งเปรียบเทียบประสิทธิผลของการรักษาในสองกลุ่ม โดยใช้การสุ่มผู้เข้าร่วม (randomized) เป็นกลุ่มทดลอง (Experimental group) และกลุ่มควบคุม (Control group) และการปิดบังผู้ประเมินผล (assessor-blinded) ผู้เข้าร่วมการวิจัยทุกคนได้ลงนามในแบบฟอร์มแสดงความยินยอมก่อนเข้าร่วมการศึกษา และการศึกษานี้ได้รับการอนุมัติจากคณะกรรมการจริยธรรมการวิจัยโรงพยาบาลหาดใหญ่

การศึกษานี้ดำเนินการตามแนวทางของ แนวปฏิบัติมาตรฐานสำหรับการรายงานการทดลองแบบสุ่ม (CONSORT) ปี ค.ศ. 2025^44^ และมีการแนบรายการตรวจสอบ (CONSORT checklist) ครบถ้วน และเมื่อได้รับการอนุมัติจากคณะกรรมการจริยธรรม การศึกษานี้จะลงทะเบียนที่ ClinicalTrials.gov พร้อมระบุวันที่เริ่มต้นและสิ้นสุดอย่างชัดเจน ปรับแก้ตามคำแนะนำคณะกรรมการ Full Board Review มี Register Protocol จะลงทะเบียนหลังได้ ได้รับการอนุมัติจากคณะกรรมการจริยธรรม อยู่ด้านหลังเอกสารอ้างอิง ตรงภาคผนวก

คำแนะนำจากคณะกรรมการ กรณีที่ผู้วิจัยต้องการทำ RCT ให้ เพิ่ม การควบคุมภัยคุกคามต่อความถูกต้องภายใน (internal threats) และ ควบคุมภัยคุกคามต่อความถูกต้องภายนอก (external threats)

**การควบคุมภัยคุกคามต่อความถูกต้องภายใน (Internal Validity)** ดำเนินการ Randomization เพื่อกระจายความแตกต่างของกลุ่มตัวอย่างให้เท่าเทียม มีกลุ่มควบคุมเพื่อเปรียบเทียบผลลัพธ์กับกลุ่มที่ไม่ได้รับการแทรกแซง และการปิดบังผู้ประเมินผล วัดผลลัพธ์ด้วยวิธีที่มีความน่าเชื่อถือ และใช้เครื่องมือวัดที่ได้มาตรฐาน

**การควบคุมภัยคุกคามต่อความถูกต้องภายนอก (External Validity)** ดำเนินการสุ่มตัวอย่างจากประชากรเป้าหมายที่มีลักษณะเฉพาะเจาะจง ใกล้เคียงกับกลุ่มที่จะนำผลการศึกษาไปใช้ ควบคุม เวลาและสถานที่ในการเก็บข้อมูล โดยจำกัดเฉพาะผู้ป่วยในพื้นที่อำเภอหาดใหญ่ภายในช่วงเวลาที่กำหนด เพื่อลดอิทธิพลจากบริบทเฉพาะด้านเวลาและสถานที่ที่อาจส่งผลกระทบต่อผลลัพธ์ให้ รายละเอียดของลักษณะกลุ่มตัวอย่างอย่างชัดเจน เพื่อให้ผู้อ่านสามารถพิจารณาได้ว่า ผลการศึกษาสามารถนำไปประยุกต์ใช้กับประชากรกลุ่มอื่นได้หรือไม่

ผู้วิจัยจึงขอสรุปเป็นตารางแนวทาง การควบคุมภัยคุกคามต่อความเที่ยงตรงภายใน (Internal Validity) และความเที่ยงตรงภายนอก (External Validity) เพิ่มเติมดังตารางต่อไปนี้

**ตารางที่ 1** แนวทางการควบคุม **Internal Threats** (ความเที่ยงตรงภายใน) เพื่อให้แน่ใจว่า “ผลการรักษา” เกิดจาก rPMS จริง ไม่ใช่จากปัจจัยแทรกซ้อน:

| **ประเภท** | **วิธีการควบคุมในงานวิจัยนี้** |
| --- | --- |
| **1. Selection bias** | ใช้ Block Randomization แบ่งกลุ่มแบบสุ่มให้มีจำนวนกลุ่มเท่ากัน |
| **2. History effect** | กำหนดระยะเวลาการทดลองสั้น (4 สัปดาห์) และใช้ตารางเวลารักษาเหมือนกันทุกกลุ่ม |
| **3. Maturation** | ระยะเวลาสั้นเพื่อลดผลจากการเปลี่ยนแปลงตามธรรมชาติ เช่น การหายเอง |
| **4. Testing effect** | ใช้มาตรวัดที่ผ่านการทดสอบความเชื่อถือได้ (reliable tools) และมีช่วงห่างระหว่างการวัดที่เหมาะสม |
| **5. Instrumentation** | ใช้อุปกรณ์และเครื่องมือวัดเดียวกัน เช่น VAS, PPT, NDI โดยมีผู้ประเมินคนเดียว (blinded assessor) |
| **6. Experimental mortality** | กำหนดเกณฑ์ชัดเจนเกี่ยวกับ dropout และมีการวิเคราะห์ตามหลัก intention-to-treat หากจำเป็น |
| **7. Placebo effect / Hawthorne effect** | ใช้ “กลุ่มควบคุม” ที่ได้รับ rPMS เหมือนกัน แต่ต่างกันที่ระยะเวลาเท่านั้น (เทียบเคียงกับ sham ได้ระดับหนึ่ง) |

**ตารางที่ 2** แนวทางการควบคุม **External Threats** (ความเที่ยงตรงภายนอก) เพื่อให้ผลลัพธ์สามารถนำไปใช้กับกลุ่มอื่นได้จริง:

| **ประเภท** | **วิธีการควบคุมในงานวิจัยนี้** |
| --- | --- |
| **1. Interaction of selection and treatment** | กำหนดกลุ่มตัวอย่างอย่างหลากหลาย (ทั้งเพศชาย-หญิง ช่วงอายุทำงาน) |
| **2. Interaction of setting and treatment** | ทำการศึกษาในบริบทของโรงพยาบาลรัฐซึ่งมีความใกล้เคียงกับคลินิกทั่วไป |
| **3. Interaction of history and treatment** | ให้คำแนะนำการดูแลตนเองเหมือนกันทุกกลุ่ม เพื่อสะท้อนแนวทางที่คลินิกจริงทำ |
| **4. Multiple treatment interference** | ควบคุมการใช้ยาอื่นหรือการรักษาอื่น โดยระบุให้หยุดหรือหลีกเลี่ยงในช่วงที่เข้าร่วมการศึกษา และบันทึก co-intervention ที่อาจเกิดขึ้น |

**Assessed for eligibility (n=66)**

**Excluded (n=...)**

- ถ้ามีให้ระบุเหตุผลและจำนวน

**Randomized (n=33)**

**Enrolment**

- ได้ rPMS สัปดาห์ละ 1 ครั้ง ตาม protocol A รวมทั้งหมด 4 สัปดาห์

- ได้รับการวัด VAS PPT และ NDI 3 ครั้ง ครั้งที่ 1 ก่อนได้รับการรักษา ครั้งที่ 2 คือ สัปดาห์ที่ 2 และครั้งที่ 3 คือ สัปดาห์ที่ 4

- การหยุดการให้การรักษา (ระบุเหตุผล) (n=…)

- นำมาวิเคราะห์เพื่อผลลัพธ์หลัก VAS (n=33)

- นำมาวิเคราะห์เพื่อผลลัพธ์รอง PPT and NDI-TH (n=33)

- ไม่ได้นำมาวิเคราะห์ (ระบุเหตุผล) (n=…)

- ได้ rPMS สัปดาห์ละ 1 ครั้ง ตาม protocol B รวมทั้งหมด 4 สัปดาห์

- ได้รับการวัด VAS PPT และ NDI 3 ครั้ง ครั้งที่ 1 ก่อนได้รับการรักษา ครั้งที่ 2 คือ สัปดาห์ที่ 2 และครั้งที่ 3 คือ สัปดาห์ที่ 4

- การหยุดการให้การรักษา (ระบุเหตุผล) (n=…)

- นำมาวิเคราะห์เพื่อผลลัพธ์หลัก VAS (n=33)

- นำมาวิเคราะห์เพื่อผลลัพธ์รอง PPT and NDI-TH (n=33)

- ไม่ได้นำมาวิเคราะห์ (ระบุเหตุผล) (n=…)

**Analysis**

**Follow-Up**

**กลุ่มทดลอง (n=33) : Protocol A**

**- ได้ rPMS ช่วงที่ 1: กระตุ้นบริเวณรอบกล้ามเนื้อทราพีเซียสส่วนบน** ความถี่: 3–30 Hz เริ่มความเข้มที่ 20% ของกำลังสูงสุดของเครื่อง และเพิ่มขึ้นทีละ 5% เพิ่มจนถึงระดับที่ผู้เข้าร่วม รู้สึกถึงการกระตุ้นแต่ไม่เจ็บปวด ระยะเวลา: 10 นาที

**- ช่วงที่ 2: กระตุ้นที่ Trigger Point โดยตรง** ความถี่: 30 Hz เริ่มต้นที่ 2% เพิ่มจนถึงระดับที่ผู้เข้าร่วม รู้สึกถึงการกระตุ้นแต่ไม่เจ็บปวด รูปแบบพัลส์: พัลส์ 1 วินาที สลับพัก 3 วินาที ระยะเวลา: 5 นาที รวมระยะเวลาการรักษา 15 นาที

- **การยืดเหยียดกล้ามเนื้อ** บริเวณกล้ามพีเซียสส่วนบน 10 ครั้งต่อเซต ค้างไว้ครั้งละ 10 วินาทีทั้งสองข้างทุกวัน วันละ 2 เซต

**กลุ่มควบคุม (n = 33) Protocol B**

**- ได้ rPMS ช่วงที่ 1: กระตุ้นบริเวณรอบกล้ามเนื้อทราพีเซียสส่วนบน** ความถี่: 3–30 Hz เริ่มความเข้มที่ 20% ของกำลังสูงสุดของเครื่อง และเพิ่มขึ้นทีละ 5% เพิ่มจนถึงระดับที่ผู้เข้าร่วม รู้สึกถึงการกระตุ้นแต่ไม่เจ็บปวด ระยะเวลา: 5 นาที

**- ช่วงที่ 2: กระตุ้นที่ Trigger Point โดยตรง** ความถี่: 30 Hz เริ่มต้นที่ 2% เพิ่มจนถึงระดับที่ผู้เข้าร่วม รู้สึกถึงการกระตุ้นแต่ไม่เจ็บปวด รูปแบบพัลส์: พัลส์ 1 วินาที สลับพัก 3 วินาที ระยะเวลา: 5 นาที รวมระยะเวลาการรักษา 10 นาที

- **การยืดเหยียดกล้ามเนื้อ** บริเวณกล้ามพีเซียสส่วนบน 10 ครั้งต่อเซต ค้างไว้ครั้งละ 10 วินาทีทั้งสองข้างทุกวัน วันละ 2 เซต

**Allocation**

รูปที่ 1: **แสดง** CONSORT **2025** Flow Diagram

**8.2 ประชากรที่ศึกษา กลุ่มตัวอย่าง**

**ประชากร (Participants)**

ประชากรในการวิจัยนี้ คือ ประชากรที่มีกลุ่มอาการปวดกล้ามเนื้อทราพีเซียสส่วนบน (Upper trapezius myofascial pain syndrome) ซึ่งมีอาการปวดและพังผืดในกล้ามเนื้อนี้เป็นอาการหลัก	ตามเกณฑ์การวินิจฉัยของ Simon’s criteria^1,24,45^ ที่มาเข้ารับการรักษาที่แผนกเวชกรรมฟื้นฟู โรงพยาบาลหาดใหญ่ จังหวัดสงขลา และประชากรกลุ่มตัวอย่างได้รับการวินิจฉัยว่าเป็นกลุ่มอาการปวดกล้ามเนื้อทราพีเซียสส่วนบน (Upper trapezius myofascial pain syndrome) จากแพทย์เวชศาสตร์ฟื้นฟู

**การคำนวณขนาดกลุ่มตัวอย่าง (Sample size calculation)**

การศึกษานี้มีการประมาณขนาดตัวอย่างสำหรับการทดลองแบบสุ่มที่มีการควบคุม (RCT) ซึ่งมีการประเมินผลลัพธ์ที่รายงานโดยผู้ป่วยซ้ำหลายครั้งแบบ Analysis of covariance (ANCOVA)

กรณีวางแผนวิจัยในผู้ป่วย 2 กลุ่ม กลุ่มทดลอง และกลุ่มควบคุม ติดตามระดับคะแนนความปวด ที่เริ่มต้น (สัปดาห์ที่ 0) สัปดาห์ที่ 2 และสัปดาห์ที่ 4 (3 ครั้ง) เปรียบเทียบระดับคะแนนความปวด หลังโปรแกรมการรักษา โดยปรับระดับคะแนนความปวด ก่อนโปรแกรมให้เท่ากันทางสถิติ และได้ผลของคะแนนความปวดที่ลดลงของการศึกษาของ Kim และคณะ ในปี 2021^40^ โดยผู้ป่วยกลุ่มที่ได้การรักษาด้วย PMS 10 นาทีมีค่า mean VAS = 2 standard deviation (SD) = 0.8 ได้มาจาก ผลของคะแนนความปวด คือ outcome เรื่อง Visual Analogue Scale Visual Analogue Scale (VAS) table 1 และ table 2^40^ จะเห็นว่า SD หลัง EG (experimental group) = 0.08

| **Variable** | **Group** | **n** | **Mean** | **SD** |
| --- | --- | --- | --- | --- |
| VAS | EG | 13 | 2.0 | 0.08 |
| VAS | CG | 12 | 4.2 | 1.14 |

จึงต้องใช้ค่า Pooled SD ซึ่งมีความหมายดังนี้ ส่วนเบี่ยงเบนมาตรฐานรวม (Pooled Standard Deviation) คือ ค่าเฉลี่ยถ่วงน้ำหนักของส่วนเบี่ยงเบนมาตรฐานจากหลายกลุ่ม โดยมีวัตถุประสงค์เพื่อนำไปเปรียบเทียบหรือคำนวณค่าทางสถิติให้มีความแม่นยำมากขึ้น โดย Pooled Standard Deviation ใช้เพื่ออะไร

1. คำนวณขนาดผล (Effect Size) เช่น Cohen’s d เพื่อวัดความแตกต่างระหว่างกลุ่มตัวอย่าง 2 กลุ่ม โดยใช้ส่วนเบี่ยงเบนมาตรฐานรวมในตัวหาร

2. เปรียบเทียบความแตกต่างของค่าเฉลี่ยระหว่างกลุ่ม เช่น ในการทำ t-test ที่กลุ่มมีความแปรปรวนใกล้เคียงกัน

3. เพิ่มความแม่นยำของการวิเคราะห์ เพราะช่วยลดผลกระทบจากขนาดกลุ่มไม่เท่ากัน

สูตรการคำนวณ

แทนค่า

ผลลัพธ์ Pooled Standard Deviation ≈ 0.79 คิดเป็น 0.8 และคำนวณ Cohen’s d เพื่อดูขนาดผล (effect size) ระหว่างกลุ่ม โดยจากข้อมูลด้านบน สามารถนำ มาใช้คำนวณ ดังนี้ Pooled Standard Deviation (SD รวม) ที่เราคำนวณได้ก่อนหน้านี้ เพื่อคำนวณ Cohen’s d ซึ่งใช้วัด ขนาดผล (Effect Size) ระหว่างกลุ่มทดลอง (EG) และกลุ่มควบคุม (CG)

แทนค่า โดย: M 1 = ค่าเฉลี่ยของกลุ่มทดลอง (EG) = 2.0 M 2 = ค่าเฉลี่ยของกลุ่มควบคุม (CG) = 4.2

Pooled Standard Deviation =0.8 ดังนั้น d = 2.0-4.2/0.8 = -2.75

Cohen’s d = 2.75 (ค่าลบแสดงว่า กลุ่มทดลองมีค่าเฉลี่ยต่ำกว่ากลุ่มควบคุม ซึ่งสอดคล้องกับการลดลงของค่า VAS) การการแปลผลขนาดผล (Effect Size Interpretation):

| **ค่า d** | **ขนาดผล (Effect Size)** |
| --- | --- |
| 0.2 | เล็ก (small) |
| 0.5 | กลาง (medium) |
| 0.8 | ใหญ่ (large) |
| ≥ 2.0 | ใหญ่มาก (very large) |

สรุป ค่าขนาดผล Cohen’s d = 2.75 แสดงว่า การรักษาด้วย rPMS มี ผลในระดับสูงมาก ต่อการลดอาการปวดตามค่า VAS เป็นผลลัพธ์ที่มีนัยสำคัญทางคลินิก

และคาดการณ์ว่า กลุ่มที่ได้ rPMS 15 นาที โดยอ้างอิงการศึกษาของ Jiravichitchai และคณะ^43^ จากการออกแบบงานวิจัยนี้ จะมีคะแนนความปวดลดลงมากกว่าอีกกลุ่มร้อยละ 25 ทำให้มี mean VAS = 1.5 แทนค่าในสูตรชอง Borm และคณะ ในปี ค.ศ. 2007^46^

alpha	=	0.0500 (two-sided)

power	=	0.8000

MCID (difference between means)	=	(2-1.5) = 0.5

SD	=	0.8

correlation between follow-up measurements	=	0.6 (จากการศึกษาของWalters และคณะ 2019^47^)

**Estimated required sample sizes:**	N = 26 ต่อกลุ่ม

และเพิ่มจำนวนเผื่อผู้ที่ทำการศึกษาอาจถอนตัวจากการศึกษา คิดเป็นร้อยละ 20 เท่ากับ 6.5 จะได้จำนวนประชากรในกลุ่มทดลองและกลุ่มควบคุมกลุ่มละ 33 คน รวมทั้งสิ้น 66 คน

**8.3 การคัดเลือกผู้รับการวิจัย (อาสาสมัคร) ที่เข้าร่วมในโครงการวิจัย**

**เกณฑ์คัดเข้าผู้เข้าร่วมการศึกษา (Inclusion criteria)**

- มีอายุระหว่าง 18 - 55 ปี
- ผู้ป่วยที่ได้รับการวินิจฉัยโดยแพทย์ว่าเป็นกลุ่มอาการ MPS ที่บริเวณคอบ่า ที่มี active myofascial pain trigger point ตามเกณฑ์การวินิจฉัยของ Simon’s criteria
- ผู้ที่มีอาการปวดกล้ามเนื้อทราพีเซียสส่วนบนในระยะมากกว่าหรือเท่ากับ 3 เดือน
- ผู้ป่วยต้องมีคะแนนความปวดใน Visual Analog Scale (VAS) มากกว่าหรือเท่ากับ 4
- ผู้ป่วยเข้าใจภาษาไทยและสามารถอ่านเขียนตอบแบบสอบถาม และติดตามอาการต่อเนื่องได้
- ยินยอมเข้าร่วมงานวิจัย

**8.4 เกณฑ์การถอนอาสาสมัครออกจากโครงการวิจัย**

**เกณฑ์คัดออกผู้เข้าร่วมการศึกษา (Exclusion criteria)**

- ผู้ป่วยโรคกระดูกต้นคอเสื่อมเรื้อรัง (cervical spondylosis) มีการเลื่อนของกระดูกต้นคอ (cervical spondylolisthesis) หรือ มีกระดูกต้นคอผิดรูป (cervical deformities หรือ scoliosis) และมีความไม่มั่นคงของกระดูกต้นคอ (C1-C2 dislocation) ผู้ป่วยมีอาการแสดงที่มีความผิดปกติทางระบบประสาท (neurological deficits) เช่น radiculopathy, myelopathy เป็นต้น หรือ ตรวจร่างกายพบความผิดปกติของสัญญาณของการทำงานผิดปกติของเซลล์ประสาทสั่งการส่วนบน (upper motor neuron signs)
- มีโรคประจำตัวที่เกี่ยวข้องกับระบบประสาทและกล้ามเนื้อ เช่น โรคระบบประสาทส่วนกลาง
- ผู้ที่มีความผิดปกติที่กระดูกคอ มีโรคข้อรูมาตอยด์ (rheumatoid arthritis) หรือ ข้ออักเสบอื่น ๆบริเวณกระดูกต้นคอ เช่น ankylosing spondylitis และ systemic lupus erythematosus
- มีการบาดเจ็บของกระดูกคอ หรือเคยผ่าตัดต้นคอ
- ผู้ป่วยมีโรคประจำตัว ที่ควบคุมอาการไม่ได้ ได้แก่ อาการไม่คงที่ของระบบหัวใจและหลอดเลือด (instability of cardiovascular disease) ความดันหิตสูงที่ควบคุมระดับความดันไม่ได้ มากกว่าหรือเท่ากับ 160/110 mmHg ผู้ป่วยมีโรคประจำตัวเบาหวานที่ควบคุมระดับน้ำตาลไม่ได้ น้อยกว่า 60 mg% หรือ มากกว่าหรือเท่ากับ 250 mg%
- ผู้ป่วยหญิงตั้งครรภ์หรือให้นมบุตร
- ผู้ป่วยที่มีอาการและอาการแสดงของ โรคปวดกล้ามเนื้อและเนื้อเยื่อพังผืดเรื้อรังทั่วร่างกาย (Fibromyalgia)
- มีประวัติการผ่าตัดบริเวณคอหรือบ่า ที่อาจส่งผลกระทบต่อผลการรักษา
- ผู้ที่มีอุปกรณ์อิเล็กทรอนิกส์ หรือโลหะในตัว เช่น Cardiac pacemaker ผู้ป่วยที่มีคลิปหนีบเส้นเลือดโปงพอง

-	ผู้ป่วยโรคมะเร็งหรือมีก้อนเนื้องอกที่บริเวณคอ

-	ผู้ที่เคยได้รับการรักษาด้วย PMS มาก่อนในช่วง น้อยกว่า 3 เดือน

-	ผู้ป่วยที่มีอาการไม่พึงประสงค์จากการใช้กลุ่มยาต้านการอักเสบที่ไม่ใช่สเตียรอยด์ (NSAIDs)

**8.5 เกณฑ์การยุติโครงการวิจัย**

- ผู้เข้าร่วมวิจัยมีภาวะแทรกซ้อนหรือผลข้างเคียงที่รุนแรง ที่อาจเป็นอันตรายต่อสุขภาพของผู้เข้าร่วม เช่น มีอาการปวดจนทนไม่ไหว ทนความร้อนที่อาจจะเกิดขึ้นหัวกระตุ้นไม่ได้ หรือมีอาการเมื่อยล้า อ่อนแรง หลังได้รับ rPMS หรือ ภาวะแทรกซ้อนจากการรักษาอื่น ๆ ที่ส่งผลต่อสุขภาพ
- ผู้เข้าร่วมวิจัยถอนความยินยอม ในการเข้าร่วมวิจัยโดยไม่จำเป็นต้องให้เหตุผล และหากมีการถอนตัวจากการวิจัย ผู้เข้าร่วมจะได้รับการหยุดรักษาหรือได้รับการรักษาอื่น ๆ ตามความเหมาะสม

**8.6 การเก็บรวบรวมข้อมูล**

คัดเลือกอาสาสมัครจากผู้ป่วยรายใหม่ของแผนกเวชกรรมฟื้นฟูที่มีคุณสมบัติตามเกณฑ์การคัดเข้า ผู้วิจัยจึงจะขอความร่วมมือจากแพทย์หรือพยาบาลเป็นผู้คัดเลือกอาสาสมัครตามเกณฑ์คัดเข้าของโครงการ หากอาสาสมัครสนใจเข้าร่วมโครงการจะมีผู้ช่วยวิจัยไปอธิบายรายละเอียดเกี่ยวกับการวิจัย เมื่ออาสาสมัครตัดสินใจเข้าร่วมในการวิจัยและลงลายมือชื่อในแบบยินยอมอาสาสมัครแล้ว ผู้ช่วยวิจัยที่ผ่านการซักซ้อมทำความเข้าใจร่วมกันแล้วในการแจ้งรายละเอียด แนะนำโครงการวิจัย จะเป็นผู้ขอความยินยอมในการวิจัยและเป็นผู้นัดหมายผู้ป่วยเข้าสู่กระบวนการวิจัย

**8.7 ขั้นตอนการดำเนินงานวิจัย**

**1. วิธีการแบ่งกลุ่มตัวอย่าง**

ผู้เข้าร่วมวิจัยจะถูกสุ่มเข้าสู่กลุ่มการศึกษาโดยผู้ช่วยนักวิจัย ในการวิจัยครั้งนี้ได้ใช้วิธี Block Randomization เพื่อจัดสรร (Allocation) จำนวนทั้งหมด 66 คนเข้าสู่ 2 กลุ่ม กลุ่มทดลอง 33 คน และ กลุ่มควบคุม 33 คน อย่างสมดุล โดยกำหนด ขนาดบล็อก (block size) เท่ากับ 4 คนต่อบล็อก ซึ่งในแต่ละบล็อกจะมีการสุ่มให้ผู้เข้าร่วม 2 คนอยู่ในกลุ่มทดลอง และ 2 คนอยู่ในกลุ่มควบคุม โดยแบ่งให้มีจำนวนผู้เข้าร่วมเท่า ๆ กันในแต่ละกลุ่ม ลำดับของการจัดสรรภายในแต่ละบล็อกถูก สุ่มแบบสุ่มสมบูรณ์ (randomized within each block) เพื่อป้องกันความเอนเอียงที่อาจเกิดขึ้นจากการจัดกลุ่มล่วงหน้า และเพื่อให้แน่ใจว่าผู้เข้าร่วมถูกแจกจ่ายอย่างสมดุลระหว่างสองกลุ่มตลอดระยะเวลาของการเก็บข้อมูล การสุ่มจัดกลุ่มทำโดยใช้โปรแกรมคอมพิวเตอร์ (https://www.random.org/integer-sets) เพื่อสร้างลำดับการจัดกลุ่มแบบ block randomization ผลลัพธ์ของการสุ่มถูกปกปิดจากผู้เก็บข้อมูลและผู้ประเมินผล (single-blinded) เพื่อป้องกันอคติจากการทราบกลุ่มของผู้เข้าร่วม ซึ่งลำดับเหล่านี้จะถูกสุ่มเลือกโดยระบบและไม่สามารถคาดเดาได้ เช่น แบบที่ 1 ทดลอง – ควบคุม – ทดลอง – ควบคุม และแบบที่ 2 ควบคุม – ควบคุม – ทดลอง – ทดลอง เป็นต้น การทำ Block Randomization แบบนี้ช่วยให้จำนวนผู้เข้าร่วมในแต่ละกลุ่มมีความสมดุลตลอดช่วงการรับสมัคร ลดความเอนเอียง (allocation bias) ที่อาจเกิดขึ้นจากการจัดกลุ่มแบบสุ่มทั่วไป ผลลัพธ์การจัดกลุ่มถูก ปกปิดจากผู้ประเมินผล (single-blinded) เพื่อรักษาความน่าเชื่อถือของผลลัพธ์

**2. การรวบรวมข้อมูลพื้นฐาน**

2.1 ผู้วิจัยคัดเลือกผู้เข้าร่วมวิจัยที่มีคุณสมบัติตามเกณฑ์กำหนด แจ้งวัตถุประสงค์ ขั้นตอนการวิจัย และให้ผู้เข้าร่วมวิจัยเซ็นชื่อหลังจากยินยอมเข้าร่วมวิจัยโดยความเต็มใจ แล้วนัดหมายผู้เข้าร่วมวิจัยเพื่อดำเนินการรักษา

2.2 บันทึกข้อมูลพื้นฐานผู้ป่วยแบบบันทึกข้อมูลผู้เข้าร่วมการวิจัย (Case Record Form - CRF) โดยผู้ช่วยวิจัย

2.3 อาสาสมัครได้รับการตรวจร่างกายโดยแพทย์เวชศาสตร์ฟื้นฟูเพื่อวินิจฉัย ระบุตำแหน่งกดเจ็บโดยผู้วิจัย อาสาสมัครให้คะแนนความปวดโดยใช้ ระดับความปวดโดยใช้มาตรวัดความปวดด้วยสายตา (Visual Analog Scale :VAS) การวัดค่า**ความทนทานต่อความเจ็บปวดจากแรงกด** (Pressure Pain Threshold: PPT) และทำแบบประเมินดัชนีวัดความบกพร่องความสามารถของคอฉบับภาษาไทย (Neck Disability Index-Thai : NDI-TH) ก่อนได้รับการรักษา

2.4 พยาบาลวิชาชีพทำการวัดความดันและชีพจรก่อนทำการรักษา และหลังการรักษา

คัดเลือกอาสาสมัครจากผู้ป่วยรายใหม่ของแผนกเวชกรรมฟื้นฟูที่มีคุณสมบัติตามเกณฑ์การคัดเข้า ผู้วิจัยจึงจะขอความร่วมมือจากแพทย์หรือพยาบาลเป็นผู้คัดเลือกอาสาสมัครตามเกณฑ์คัดเข้าของโครงการ หากอาสาสมัครสนใจเข้าร่วมโครงการจะมีผู้ช่วยวิจัยไปอธิบายรายละเอียดเกี่ยวกับการวิจัย เมื่ออาสาสมัครตัดสินใจเข้าร่วมในการวิจัยและลงลายมือชื่อในแบบยินยอม

**3. การรักษา**

3.1 อาสาสมัครทั้งสองกลุ่มจะได้รับโปรแกรมการรักษาแบบอนุรักษ์ที่เป็นมาตรฐาน (Standard treatment) ร่วมด้วย โดยประกอบด้วยการให้ความรู้เกี่ยวกับการปรับพฤติกรรมในกิจวัตรประจำวัน และการแนะนำการออกกำลังกายเพื่อยืดกล้ามเนื้อ (stretching exercise) ของบริเวณกล้ามเนื้อทราพีเซียสส่วนบนด้วยตนเอง ซึ่งเป็นการรักษาตามมาตรฐาน โดยให้ทำ วันละ 10 ครั้ง ค้างไว้ครั้งละ 10 วินาทีทั้งสองข้างทุกวันตลอดระยะการศึกษา โดยให้ผู้เข้าร่วมทำการออกกำลังกายท่าดังกล่าวจำนวน 10 ครั้งต่อเซต วันละ 2 เซต^48^

3.2 อาสาสมัครได้รับการรักษาด้วย rPMS 2 รูปแบบการรักษา เครื่องมือที่ใช้ คือ เครื่องกระตุ้นประสาทส่วนปลายด้วยแม่เหล็กไฟฟ้า เครื่องหมายการค้า REMED rehabilitation medical company รุ่น Salus Talent Pro สัปดาห์ละ 1 ครั้ง รวมทั้งหมด 4 สัปดาห์ ตามระเบียบการทดลองกลุ่มทดลองและกลุ่มควบคุม

ผู้วิจัยสนใจและต้องการทราบเรื่อง parameters ของการกำหนดระยะเวลาของการรักษา จากการศึกษาของการศึกษาของ Kim และคณะในปี ค.ศ. 2021^40^ ที่ใช้ช่วงเวลา 10 นาที ให้ผลลัพธ์ในการรักษาเรื่องการลดปวดอย่างมีนัยสำคัญทางสถิติ ที่ระยะการรักษา 4 สัปดาห์ ถือเป็นกลุ่มควบคุม ส่วนกลุ่มทดลอง เพื่อเปรียบเทียบกับการศึกษาของ Jiravichitchai และคณะ^43^ ที่พบว่าใช้ช่วงเวลา 15 นาที ให้ผลลัพธ์ในการรักษาเรื่องการลดปวดอย่างมีนัยสำคัญทางสถิติ ที่ระยะการรักษาสั้น 1 สัปดาห์ ดังนั้น ทั้ง 2 ระยะเวลาการรักษายังไม่มีการศึกษาเปรียบเทียบกัน ผู้วิจัยจึงต้องการสร้าง parameters เรื่องระยะเวลาที่เหมาะสม และดูแลผู้ป่วยให้ยาวนานขึ้นจาก ตามการศึกษาของ Jiravichitchai และคณะที่ 1 สัปดาห์ และมีมาตราฐานการรักษาของ Kim และคณะในปี ค.ศ. 2021^40^ระยะการรักษา 4 สัปดาห์ จึงเป็นที่มาของการดำเนินกำหนด Protocol A และ B

แสดงรายละเอียดกลุ่มทดลองและกลุ่มควบคุม ได้ดังนี้

**กลุ่มทดลอง (Protocol A) :** Progressive-intensity and trigger point

**ช่วงที่ 1: กระตุ้นบริเวณรอบกล้ามเนื้อ**

ความถี่: 3–30 Hz เริ่มความเข้มที่ 20% ของกำลังสูงสุดของเครื่อง และเพิ่มขึ้นทีละ 5% เพิ่มจนถึงระดับที่ผู้เข้าร่วม รู้สึกถึงการกระตุ้นแต่ไม่เจ็บปวด กล้ามเนื้อและพังผืดบริเวณทราพีเซียสส่วนบน

ระยะเวลา: 10 นาที

**ช่วงที่ 2: กระตุ้นที่ Trigger Point โดยตรง**

ความถี่: 30 Hz เริ่มต้นที่ 2% เพิ่มจนถึงระดับที่ผู้เข้าร่วม รู้สึกถึงการกระตุ้นแต่ไม่เจ็บปวด

รูปแบบพัลส์: พัลส์ 1 วินาที สลับพัก 3 วินาที

ระยะเวลา: 5 นาที

**รวมระยะเวลาทั้ง 2 ช่วงการรักษา** เท่ากับ 15 นาที

**กลุ่มควบคุม (Protocol B) :** Progressive-intensity and trigger point

**ช่วงที่ 1: กระตุ้นบริเวณรอบกล้ามเนื้อ**

ความถี่: 3–30 Hz เริ่มความเข้มที่ 20% ของกำลังสูงสุดของเครื่อง และเพิ่มขึ้นทีละ 5% เพิ่มจนถึงระดับที่ผู้เข้าร่วม รู้สึกถึงการกระตุ้นแต่ไม่เจ็บปวด กล้ามเนื้อและพังผืดบริเวณทราพีเซียสส่วนบน

ระยะเวลา: 5 นาที

**ช่วงที่ 2: กระตุ้นที่ Trigger Point โดยตรง**

ความถี่: 30 Hz เริ่มต้นที่ 2% เพิ่มจนถึงระดับที่ผู้เข้าร่วม รู้สึกถึงการกระตุ้นแต่ไม่เจ็บปวด

รูปแบบพัลส์: พัลส์ 1 วินาที สลับพัก 3 วินาที

ระยะเวลา: 5 นาที

**รวมระยะเวลาทั้ง 2 ช่วงการรักษา** เท่ากับ 10 นาที

3.3 การรักษาอื่นๆ อาสาสมัครได้รับยา Diclofenac ขนาด 25 มิลลิกรัม เพื่อใช้เป็นยาที่จัดเตรียมไว้ใช้ในกรณีที่ผู้ป่วยมีอาการมากขึ้นหรือไม่ตอบสนองต่อการรักษาหลัก (rescue drug) หากมีอาการปวดมากโดยไม่สามารถลดปวดด้วยการยืดกล้ามเนื้อ หรือ ประคบเย็นได้

**4. การเก็บและบันทึกข้อมูล**

- การเก็บบันทึกข้อมูลจะถูกกระทำโดยผู้ร่วมวิจัย ที่ไม่เกี่ยวข้องกับการให้การรักษา ในทั้ง 2 กลุ่ม

- โดยผู้ร่วมวิจัยจะทำการบันทึกข้อมูล 3 ครั้ง คือ ครั้งแรกก่อนการวิจัย ครั้งที่ 2 คือ สัปดาห์ที่ 2 และครั้งที่ 3 คือ สัปดาห์ที่ 4 อ้างอิงจากการศึกษาของ Nathinee และคณะ ปี พ.ศ. 2566^41^ ที่การวัดผลระหว่างกลางของการทดลอง (เช่น การวัดครั้งที่ 2 ในสัปดาห์ที่ 2) มีความสำคัญในงานวิจัยเพื่อให้เห็นแนวโน้มของผลลัพธ์ และอาจเห็นการเปลี่ยนแปลงที่เป็น non-linear change ได้ รวมถึงอาจทำให้สามารถวิเคราะห์แนวโน้มภายในตัวบุคคลได้ดียิ่งขึ้น

**8.8 เครื่องมือที่ใช้ในการวิจัย**

**8.8.1 เครื่องมือที่ใช้ในการรวบรวมข้อมูล ประกอบด้วย**

1) แบบบันทึกข้อมูลผู้เข้าร่วมการวิจัย **เป็นเครื่องมือที่ใช้ในการรวบรวมข้อมูลพื้นฐานเกี่ยวกับผู้ป่วย ซึ่งรวมถึงข้อมูลส่วนบุคคล เช่น อายุ เพศ ระดับการศึกษา อาชีพ ลักษณะงาน จำนวนชั่วโมงที่ทำงาน ยาที่ใช้ หรือประวัติการป่วย หรือโรคประจำตัวที่อาจมีผลต่อการรักษา**

**8.8.2 เครื่องมือทีใช้วัดผลของการรักษา (Outcome measurement)**

1) การประเมินระดับความปวดโดยใช้มาตรวัดความปวดด้วยสายตา **(Visual Analog Scale :VAS)** เป็นเครื่องมือที่ใช้ในการประเมินระดับความปวดของผู้ป่วยโดยใช้แถบเส้นตรงที่มีค่าจาก 0 (ไม่ปวดเลย) ถึง 10 (ปวดรุนแรงมากที่สุด) โดยผู้ป่วยจะทำเครื่องหมายบนแถบเส้นตรงที่ตรงกับระดับความปวดที่รู้สึกในขณะนั้น วัตถุประสงค์หลักของ VAS คือการวัดระดับความปวดในช่วงเวลาต่าง ๆ ของการรักษา เช่น ก่อนและหลังการรักษา เพื่อประเมินการเปลี่ยนแปลงของอาการปวด ข้อดีของ VAS คือความง่ายในการใช้งานและความแม่นยำในการประเมินระดับความปวด แต่มีข้อจำกัดในการตีความที่ขึ้นอยู่กับบุคคลและความเข้าใจของผู้ป่วย โดยเฉพาะในผู้ที่มีอาการปวดเรื้อรังหรือระดับความปวดต่ำ การประเมินระดับความปวดVAS มีความน่าเชื่อถือในระดับปานกลางถึงดี (ค่าสหสัมพันธ์ภายในกลุ่มตั้งแต่ 0.60 ถึง 0.77) สำหรับการประเมินระดับความปวดในผู้ป่วยที่มีอาการปวดกล้ามเนื้อและกระดูกเรื้อรัง^49,50^

2) การวัดค่าความรู้สึกเจ็บปวดต่อแรงกด **(Pressure Pain Threshold: PPT)**^51^ **หรือ เกณฑ์****ความทนทานต่อความเจ็บปวดจากแรงกด เป็นการวัดระดับความเจ็บปวดที่ผู้ป่วยสามารถทนได้เมื่อถูกกดที่จุดเฉพาะในร่างกาย โดยการวัดนี้ใช้เพื่อประเมินความไวต่อการเจ็บปวดที่เกิดจากแรงกด ซึ่งมักจะใช้ในการศึกษาเกี่ยวกับอาการปวดที่เกี่ยวข้องกับกล้ามเนื้อและพังผืด เพื่อดูว่าผู้ป่วยสามารถทนต่อความเจ็บปวดได้มากน้อยเพียงใดในระหว่างการรักษา การวัด** PPT **ใช้ เครื่องมือที่ เรียกว่า** algometer **เครื่องหมายการค้า** PainTest™ FPN 100 Algometer (Wagner Instruments, Greenwich, USA) **ซึ่งเป็นเครื่องมือที่มีหัวกดเพื่อใช้ในการกดที่จุดที่มีอาการปวดหรือจุดที่ต้องการทดสอบ โดยอาจมีลักษณะเป็นด้ามที่ปลายมีแรงกดที่สามารถตั้งค่าได้** algometer **จะใช้ในการกดที่บริเวณที่ต้องการทดสอบ โดยจะเพิ่มแรงกดทีละน้อยจนกว่าผู้ป่วยจะรู้สึกเจ็บหรือถึงจุดที่ไม่สามารถทนได้ ผู้วิจัยจะเลือกจุดที่มีอาการปวดที่สุดในกล้ามเนื้อทราพีเซียสส่วนบน สำหรับการทดสอบ เครื่อง** algometer **จะถูกวางที่จุดนั้นและจะเพิ่มแรงกดทีละน้อย ผู้ป่วยจะรายงานความรู้สึกเจ็บปวดเมื่อแรงกดถึงจุดที่ไม่สามารถทนได้หรือรู้สึกเจ็บที่จุดนั้น การวัด** PPT **จะถูกบันทึกเป็นแรงกดที่ผู้ป่วยทนได้ ซึ่งมักจะใช้หน่วยเป็น กิโลกรัม** (kg) **หรือ นิวตัน (**N) **ค่าที่บันทึกได้จากการทดสอบ** PPT **คือ แรงที่ใช้ในการกระตุ้นจนทำให้ผู้ป่วยรู้สึกเจ็บ ซึ่งจะเป็นตัวบ่งชี้ถึงระดับความไวต่อการเจ็บปวด** PPT **ที่สูงขึ้นหมายความว่าผู้ป่วยทนต่อความเจ็บปวดได้มากขึ้น ขณะที่** PPT **ที่ต่ำลงหมายถึงผู้ป่วยมีความไวต่อการเจ็บปวดมากขึ้น การ** Thongbuang S **และคณะ^52^ มีการศึกษาหา**ความตรงและความเที่ยงของการวัดระดับความรู้สึกกดเจ็บด้วยเครื่องวัดชนิดออกแรงกดด้วยทั้ง manual และ digital algometer ในอาสาสมัครจํานวนทั้งหมด 30 ราย ที่มีจุดกดเจ็บบนกล้ามเนื้อทราพีเซียสส่วนบน อาสาสมัครแต่ละรายได้รับการสุ่มแบบง่าย ให้ได้รับการทดสอบด้วยเครื่องมือทั้ง 2 ชนิด มีระยะพักห่างกัน 15 นาที ทําการวัด 3 ครั้งในแต่ละเครื่องมือ แต่ละครั้งระยะพักห่างกัน 2 นาที พบว่า ทั้ง manual และ digital algometer มีค่าความเที่ยงในเกณฑ์ในระดับสูงมาก โดยมีค่า ICC เท่ากับ 0.97 (95%CI อยู่ระหว่าง 0.95 ถึง 0.99, P < 0.001) และ 0.96 (95%CI อยู่ระหว่าง 0.93 ถึง 0.98, P < 0.001) ตามลําดับ และจากผลการทดสอบความตรงพบว่ามีค่าสัมประสิทธิ์สหสัมพันธ์ของเพียร์สัน (Pearson’s correlation coefficient) เท่ากับ 0.9 (95%CI อยู่ระหว่าง 0.8 ถึง 1.0 และ P < 0.001)

3) แบบประเมินดัชนีวัดความบกพร่องความสามารถของคอฉบับภาษาไทย **(Neck Disability Index-Thai : NDI-TH)** **เป็นแบบประเมินผลกระทบของอาการปวดคอที่มีผลกระทบต่อความสามารถในการทํากิจกรรมต่าง ๆ ในชีวิตประจําวัน** (NDI-TH) **งานวิจัยของ** Uthaikhup **และคณะ ในปีค.ศ. 2011^53^ ถูกแปลมาจากต้นฉบับที่เป็นภาษาอังกฤษของ** Vernon **และคณะ ในปีค.ศ.** **1991^54^ พบว่า มีความน่าเชื่อถือและเที่ยงตรง อยู่ในการเกณฑ์ดีถึงดีมาก ในการนำมาใช้กับประชากรไทยที่มีอาการปวดคอ ประกอบด้วยข้อคําถามทั้งหมด 10 ข้อ ได้แก่ ความรุนแรงของอาการปวดคอ การดูแลตนเอง การยกของ การอ่าน อาการปวดศีรษะ การตั้งสมาธิการทำงาน การขับขี่รถ การนอนหลับและกิจกรรมนันทนาการ/การพักผ่อนหย่อนใจ คําถามแต่ละข้อมีตัวเลือก 6 ตัวเลือกและมีช่วงคะแนนตั้งแต่ 0 ถึง 5 โดย 0 หมายถึง ไม่มีอาการปวด/ไม่รบกวนการทํากิจกรรมและ 5 หมายถึง มีอาการปวดมากที่สุด/รบกวนมากจนไม่สามารถทํากิจกรรมนั้น ๆ**

**คะแนนเต็มของแบบประเมิน** NDI-TH **อยู่ระหว่าง 0-50 คะแนน โดยมีรายละเอียดดังนี้**

**- คะแนน 0-4	หมายถึงไม่มีความบกพร่อง/ไม่จํากัดการทํากิจกรรม**

**- คะแนน 5-14	หมายถึงมีความบกพร่อง/จํากัดการทํากิจกรรมในระดับน้อย**

**- คะแนน 15-24	หมายถึงมีความบกพร่อง/จํากัดการทํากิจกรรมในระดับปานกลาง**

**- คะแนน 25-34 หมายถึงมีความบกพร่อง/จํากัดการทํากิจกรรมในระดับมาก**

**- คะแนน ≥ 35	หมายถึงมีความบกพร่อง/จํากัดการทํากิจกรรมที่ทำอยู่อย่างสมบูรณ์**

**การนำ** NDI-TH **มาใช้สามารถช่วยให้การติดตามผลการรักษาและประเมินการฟื้นฟูของผู้ป่วยได้อย่างมีประสิทธิภาพ โดยแบบสอบถามนี้มีการรายงานมีความน่าเชื่อถือ จากการศึกษาของ** Uthaikhup **และคณะ^53^ ที่ศึกษาในกลุ่มผู้ป่วยคนไทยจากจังหวัดเชียงใหม่และลำพูน ที่มีอาการปวดต้นคอทั้งระยะเฉียบพลันและเรื้อรัง พบว่า มีค่าความเชื่อมั่นจากค่าสัมประสิทธิ์แอลฟาของครอนบาค (**Cronbrach’s Alpha) **เท่ากับ 0.85 เมื่อทดสอบซ้ำสำหรับการวัดทั้งหมด (**test-retest reliability) **แสดงช่วงสัมประสิทธิ์สหสัมพันธ์ภายในชั้น (**Intraclass correlation coefficient, ICC) **ระหว่าง 0.74-0.91** **และเมื่อเปรียบเทียบกับการศึกษาของ** Luksanapruksa **และคณะ^55^ ที่ทำการศึกษาที่โรงพยาบาลศิริราชกลุ่มผู้ป่วยคนไทย อายุ 20-80 ปีเพื่อทดสอบความเที่ยงตรงและความน่าเชื่อถือของแบบประเมิน** NDI-TH **พบว่า มีความน่าเชื่อถือเมื่อทำการทดสอบซ้ำเท่ากับ 0.986 ค่าความเชื่อมั่นจากค่าสัมประสิทธิ์แอลฟาของครอนบาค (**Cronbach’s alpha) **เท่ากับ 0.925 การทดสอบความเที่ยงตรงเทียบกับค่าความเจ็บปวดเท่ากับ 0.886**

**8.9 การวิเคราะห์ข้อมูล สถิติที่ใช้**

การวิเคราะห์ข้อมูลเบื้องต้น (Descriptive Statistics) จะใช้เพื่อสรุปและนำเสนอข้อมูลพื้นฐานของการศึกษาครั้งนี้ โดยจะคำนวณค่าเฉลี่ย (Mean) และส่วนเบี่ยงเบนมาตรฐาน (Standard Deviation, SD) เพื่อวิเคราะห์ค่ากลางและความแปรปรวนของระดับความปวด VAS ค่าความทนทานต่อความเจ็บปวดจากแรงกด PPT และคะแนนของ NDI-TH ในแต่ละกลุ่มผู้ป่วย ทั้งนี้เพื่อให้เข้าใจถึงการกระจายตัวของข้อมูลและระดับความปวดของผู้ป่วยในแต่ละกลุ่มการรักษา นอกจากนี้ยังใช้การแจกแจงความถี่ (Frequency) และเปอร์เซ็นต์ (Percentage) เพื่อวิเคราะห์ข้อมูลเชิงคุณภาพ เช่น ลักษณะประชากรศาสตร์ของผู้ป่วย ซึ่งจะช่วยให้สามารถนำเสนอข้อมูลประชากรกลุ่มตัวอย่างในแง่ของอายุ เพศ อาชีพ หรือปัจจัยอื่น ๆ ที่อาจมีผลต่อการรักษาและผลลัพธ์ของการวิจัย

ในการทดสอบความแตกต่างระหว่างกลุ่ม ผู้วิจัยจะใช้ linear mixed-model (LMM) สำหรับการเปรียบเทียบค่าเฉลี่ยของระดับความปวด VAS ค่าความทนทานต่อความเจ็บปวดจากแรงกด PPT และคะแนนของ NDI-TH ในแต่ละกลุ่มผู้ป่วยระหว่างสองกลุ่ม โดยกำหนด treatment group และ time รวมถึง treatment*time interaction เป็น fixed effects และ subject เป็น random effect โดยมีการกำหนดระดับนัยสำคัญที่ p < 0.05

**9. ระยะเวลาดำเนินการ**

ตั้งแต่วันที่ 1 มิถุนายน 2568 ถึง 31 พฤษภาคม 2569 รวมระยะเวลา 1 ปี

| **การดำเนินงาน** | **มิ.ย. 68** | **ก.ค. 68** | **ส.ค. 68** | **ก.ย.68** | **ต.ค. 68** | **พ.ย. 68** | **ธ.ค. 68** | **ม.ค. 69** | **ก.พ. 69** | **มี.ค. 69** | **เม.ย. 69** | **พ.ค. 69** |
| --- | --- | --- | --- | --- | --- | --- | --- | --- | --- | --- | --- | --- |
| 1. พัฒนาโครงร่างวิทยานิพนธ์ |  |  |  |  |  |  |  |  |  |  |  |  |
| 2. ส่งคณะกรรมการควบคุมจริยธรรมในการวิจัย |  |  |  |  |  |  |  |  |  |  |  |  |
| 3. พัฒนาและตรวจสอบเครื่องมือวิจัย |  |  |  |  |  |  |  |  |  |  |  |  |
| 4. การเก็บรวบรวมข้อมูล |  |  |  |  |  |  |  |  |  |  |  |  |
| 5. วิเคราะห์และอภิปรายผลการศึกษา |  |  |  |  |  |  |  |  |  |  |  |  |
| 6. การเขียนวิทยานิพนธ์ |  |  |  |  |  |  |  |  |  |  |  |  |
| 7. การส่งผลงานตีพิมพ์ |  |  |  |  |  |  |  |  |  |  |  |  |

**10. ผลที่คาดว่าจะได้รับ**

การศึกษาประสิทธิผลด้วยเครื่องกระตุ้นแม่เหล็กไฟฟ้าส่วนปลายแบบซ้ำในสองรูปแบบการรักษา เพื่อวัดอาการปวด ค่าความทนทานต่อความเจ็บปวดจากต่อแรงกด และ**ดัชนีวัดความบกพร่องความสามารถของคอ** ในผู้ป่วยกลุ่มอาการปวดกล้ามเนื้อและพังผืดบริเวณทราพีเซียสส่วนบน ซึ่งคาดว่าจะช่วยลดระดับความปวด VAS เพิ่มค่าความทนทานต่อความเจ็บปวดจากแรงกด PPT และค่าคะแนนของ NDI-TH **ดีขึ้น** ในผู้ป่วยกลุ่มอาการปวดกล้ามเนื้อและพังผืดบริเวณทราพีเซียสส่วนบน นอกจากนี้ การรักษาด้วยเทคนิคเหล่านี้คาดว่า จะช่วยกระตุ้นการไหลเวียนของเลือดและเร่งกระบวนการฟื้นฟูของกล้ามเนื้อและเนื้อเยื่อที่บาดเจ็บหรือเกร็ง อีกทั้งยังจะช่วยเพิ่มความรู้และสร้างแนวทางการรักษาที่มีประสิทธิภาพและปลอดภัยมากขึ้นในการจัดการกับอาการปวดกล้ามเนื้อและพังผืดในอนาคต

**11. ประโยชน์ที่ได้รับจากการวิจัย (ประโยชน์ ต่ออาสาสมัคร/ชุมชน/สังคม/ด้านวิชาการ)**

1. การวิจัยนี้มีประโยชน์ต่อผู้ป่วย โดยการรักษาด้วยเครื่องกระตุ้นแม่เหล็กไฟฟ้าส่วนปลายแบบซ้ำในสองรูปแบบการรักษา สามารถช่วยลดอาการปวดกล้ามเนื้อทราพีเซียสส่วนบน ซึ่งจะช่วยให้ผู้ป่วยสามารถกลับไปทำกิจกรรมประจำวันได้ดีขึ้น โดยเพิ่มระดับความสามารถของคอจากคะแนนดัชนีวัดความบกพร่องความสามารถของคอ ในการทำกิจกรรมประจำวัน จากแบบประเมิน NDI-TH จะมีค่าลดลงจากอาการปวดได้ ซึ่งสะท้อนถึงการปรับปรุงในด้านความสามารถในการทำกิจกรรมต่าง ๆ เช่น การหมุนศีรษะ การก้มเงย ฯลฯ และการรักษาด้วยทั้งสองวิธียังช่วยกระตุ้นการไหลเวียนของเลือดและการฟื้นฟูกล้ามเนื้อและเนื้อเยื่อที่ได้รับความเสียหายหรือเกร็ง ทำให้กระบวนการฟื้นฟูเร็วขึ้นและลดอาการที่เกี่ยวข้องกับการบาดเจ็บในกล้ามเนื้อหรือพังผืด

2. งานวิจัยนี้มีประโยชน์ต่อวงการแพทย์และการรักษาโดยการเพิ่มความรู้เกี่ยวกับการใช้การรักษาด้วยเครื่องกระตุ้นแม่เหล็กไฟฟ้าส่วนปลายแบบซ้ำในสองรูปแบบการรักษา ในการรักษาผู้ป่วยที่มีอาการปวดจากกล้ามเนื้อและพังผืด ซึ่งจะช่วยให้แพทย์และนักกายภาพบำบัดสามารถเลือกใช้วิธีการรักษาที่เหมาะสมกับผู้ป่วยได้มากขึ้น นอกจากนี้ การเปรียบเทียบประสิทธิผลการรักษาด้วยเครื่องกระตุ้นแม่เหล็กไฟฟ้าส่วนปลายแบบซ้ำในสองรูปแบบการรักษา จะช่วยให้สามารถประเมินและพัฒนาวิธีการรักษาที่มีประสิทธิภาพมากยิ่งขึ้นในอนาคต โดยการศึกษาครั้งนี้จะเป็นข้อมูลสำคัญในการปรับปรุงและเสริมสร้างตัวเลือกในการรักษาผู้ป่วยที่มีอาการปวดกล้ามเนื้อและพังผืดได้อย่างมีประสิทธิภาพมากขึ้น

3. งานวิจัยนี้มีประโยชน์ต่อการพัฒนางานวิจัยในอนาคต โดยข้อมูลที่ได้จากการศึกษาจะเป็นพื้นฐานสำคัญในการวิจัยเกี่ยวกับการรักษาอาการปวดจากกล้ามเนื้อและพังผืดด้วยเทคนิคต่าง ๆ รวมถึงการศึกษาผลกระทบระยะยาวที่อาจเกิดขึ้นจากการรักษาด้วยวิธีเหล่านี้ นอกจากนี้ งานวิจัยยังสามารถช่วยเสริมสร้างแนวทางการรักษาที่มีประสิทธิภาพและปลอดภัยในการจัดการกับอาการปวดจากกล้ามเนื้อทราพีเซียสและอาการพังผืด ซึ่งจะช่วยให้การรักษาผู้ป่วยในอนาคตมีประสิทธิผลมากยิ่งขึ้นและสามารถปรับปรุงวิธีการรักษาที่เหมาะสมตามแต่ละกรณีได้ดียิ่งขึ้น

**12. ด้านจริยธรรมในมนุษย์**

**12.1	ความเสี่ยง/ผลข้างเคียงที่อาจเกิดต่ออาสาสมัครและชุมชน และวิธีการลดความเสี่ยง**

ผู้วิจัยนำเสนอโครงร่างงานวิจัยเข้ารับการพิจารณาด้านจริยธรรมการวิจัยในมนุษย์ ต่อคณะกรรมการวิจัยและจริยธรรมทางการวิจัย โรงพยาบาลหาดใหญ่ จังหวัดสงขลา

เมื่อผ่านการอนุมัติ ผู้วิจัยถึงจะเริ่มทำการเก็บข้อมูลการวิจัย โดยการแนะนำตัวและชี้แจงรายละเอียดในวัตถุประสงค์และขั้นตอนการวิจัย พร้อมทั้งลงนามยินยอมอย่างเป็นลายลักษณ์อักษรสำหรับผู้ที่สมัครใจเข้าร่วมโครงการ ทั้งนี้ สามารถปฏิเสธที่จะไม่เข้าร่วมการวิจัยในครั้งนี้ หรือเมื่อสมัครเข้ามาแล้วสามารถถอนตัวออกจากการวิจัยได้ตลอดเวลา โดยไม่มีผลกระทบใด ๆ สำหรับข้อมูลที่ได้จากการวิจัยครั้งนี้จะไม่มีการเปิดเผยข้อมูลรายบุคคล โดยผู้วิจัยจะนำเสนอข้อมูลการวิจัยในภาพรวมเท่านั้น และจะทำลายข้อมูล เอกสารหลังจากเผยแพร่งานวิจัยแล้ว 5 ปี

ผู้ป่วยอาจมีผลข้างเคียงจากการใช้ PMS เช่น ผื่นแดงบริเวณที่วาง coil ซึ่งสามารถแก้ไขได้โดยใช้ intensity : 80% muscle contraction threshold ซึ่งจะไม่ทำให้เกิดอาการระคายบริเวณผิวหนังที่วาง coil

**12.2	การเคารพความเป็นส่วนตัวและการเก็บรักษาความลับของข้อมูลอาสาสมัคร**

- การบันทึกข้อมูลจะใช้รหัสผู้ป่วย (Subject Number) แทน Hospital number

- ไม่บันทึกชื่อ นามสกุล, สถานที่ทำงาน, ผู้ดูแล รวมไปถึงข้อมูลส่วนตัวที่อ้างถึงตัวตนผู้ป่วย

- ในการวิเคราะห์ข้อมูล ผู้วิเคราะห์ข้อมูลเป็นคนละคนกับผู้เก็บข้อมูลและไม่เคยเจอผู้ป่วยมาก่อน

**12.3	ประโยชน์โดยตรงต่ออาสาสมัคร และประโยชน์ต่อหน่วยงาน ชุมชน สังคม**

ผู้ป่วยจะได้รับการบำบัดรักษา ตามมาตรฐาน ที่มีประสิทธิผลในการลดปวด ใช้ระยะเวลาไม่นาน มีความปลอดภัยสูง และเหมาะสมกับตัวโรค

**12.4	กระบวนการชี้แจงและขอคำยินยอมจากอาสาสมัคร (Informed consent process)**

ผู้วิจัยและผู้ช่วยวิจัย จะอธิบายรายละเอียดเกี่ยวกับโครงการวิจัยด้วยภาษาที่เข้าใจง่าย พร้อมให้เอกสารชี้แจงแก่ผู้ป่วยได้ศึกษา เมื่อผู้ป่วยยินยอมเข้าร่วมงานวิจัย จะมีการลงนามเป็นลายลักษณ์อักษรในใบขอคำยินยอมจากผู้ป่วย สถานที่ดำเนินการที่แผนกผู้ป่วยนอก กลุ่มงานเวชกรรมฟื้นฟู โรงพยาบาลหาดใหญ่ เปิดโอกาสให้ซักถามข้อสงสัย ให้ผู้ป่วยสามารถปรึกษาครอบครัว และให้เอกสารเพื่อประกอบการตัดสินใจ

**12.5	ข้อปฏิบัติเมื่ออาสาสมัครขอถอนตัวออกจากการวิจัย**

- ผู้วิจัยจะบันทึกการขอถอนตัวลงในแบบบันทึกข้อมูลและในเอกสารชี้แจงและขอความยินยอม พร้อมทั้งลงนามของทั้งสองฝ่ายและลงวันที่

- ผู้วิจัยจะสอบถามและลงบันทึกเหตุผลการถอนตัว

- หากผู้ป่วยได้รับภาวะแทรกซ้อนจากเครื่องมือในการวิจัย ผู้วิจัยจะติดตามประสานงานให้ผู้ป่วยได้รับการรักษากับแพทย์เฉพาะทางที่เกี่ยวข้องกับการบาดเจ็บนั้น ๆ เป็นมาตรฐานเดียวกัน ตามสิทธิการรักษาของผู้ป่วยต่อไป

**12.6	การจ่ายค่าตอบแทนหรือค่าเสียเวลาแก่อาสาสมัคร**

- ไม่มี

**13. เอกสารอ้างอิง**

**1.** Simons DG, Travell JG, Simons LS. General overview. In: Johnson E, editor. Travell & Simons’ Myofascial pain and dysfunction: the trigger point manual. Vol. 1, Upper half of body. 2^nd^ ed. Baltimore: Williams & Wilkins; 1998. p 11-93.

2. Fernández-de-Las-Peñas C, Ge HY, Arendt-Nielsen L, Cuadrado ML, Pareja JA. The role of myofascial trigger points in musculoskeletal pain syndromes of the head and neck. Curr Pain Headache Rep. 2007;11(5):365–72.

3. Simons DG, Travell JG, Simons LS. Trapezius muscle. In: Johnson E, editor. Travell & Simons’ Myofascial pain and dysfunction: the trigger point manual. Vol. 1, Upper half of body. 2nd ed. Baltimore: Williams & Wilkins; 1998. p 278-307.

4. Desai, MJ, Saini V, Saini S. Myofascial pain syndrome: a treatment review. Pain Ther. 2013;2(1):21–36.

**5.**Kanjanapanang N, Chang K-V. Peripheral Magnetic Stimulation (Transcutaneous Magnetic Stimulation). In: StatPearls [Internet]. Treasure Island (FL): StatPearls Publishing; **2018 [**cited **2025** May **18].** Available from: https://www.ncbi.nlm.nih.gov/books/NBK**538332/**

**6**.Beaulieu LD, Schneider C. Repetitive peripheral magnetic stimulation to reduce pain or improve sensorimotor impairments: A literature review on parameters of application and afferents recruitment. Clin Neurophysiol **2015; 45:223-37.**

**7.**Heldmann B, Kerkhoff G, Struppler A, Havel P, Jahn T. Repetitive peripheral magnetic stimulation alleviates tactile extinction. Neuroreport. **2000; 11(14):3193–8.**

**8.**Smania N, Corato E, Fiaschi A, Pietropoli P, Aglioti SM, Tinazzi M. Repetitive magnetic stimulation A novel therapeutic approach for myofascial pain syndrome. J Neurol **2005; 252:307-14.**

9. Dragana Zarkovic, Krasimira Kazalakova. Repetitive Peripheral Magnetic Stimulation as Pain Management Solution in Musculoskeletal and Neurological Disorders – A Pilot Study. International Journal of Physiotherapy. 2016; 3(6): 671-675.

10. Lim YH, Song JM, Choi EH, Lee JW. Effects of Repetitive Peripheral Magnetic Stimulation on Patients with Acute Low Back Pain: A Pilot Study. Ann Rehabil Med. 2018;42(2):229-238.

11. Massé H, Beaulieu L, Preuss R, Schneider C. Repetitive peripheral magnetic neurostimulation of multifidus muscles combined with motor training influences spine motor control and chronic low back pain. Clin Neuro-physiol 2017;128(3):442-53.

**12**. Khedr EM, Ahmed MA, Alkady EAM, Mostafa MG, Said HG. Therapeutic effects of peripheral magnetic stimulation on traumatic branchial plexopathy: Clinical and neurophysiological study. Clinical Neurophysiology. **2012; 42:111-8.**

13. Momosaki R, Abo M, Watanabe S, Kakuda W, Yamada N, Kinoshita S. Repetitive peripheral magnetic stimulation with intensive swallowing rehabilitation for poststroke dysphagia. Neuromodulation. 2015;18(7): 630-634.

14. Fejer R, Kyvik KO, Hartvigsen J. The prevalence of neck pain in the world population: a systematic critical review of the literature. European Spine Journal. 2006;15(6), 834–848.

15. Côté P, Cassidy J D, Carroll L. The Saskatchewan health and back pain survey: the prevalence of neck pain and related disability in Saskatchewan adults. Spine. 2004;23(15):1689–1698.

16. Beltran-Alacreu H, López-de-Uralde-Villanueva I, Calvo-Lobo C, Fernández-Carnero J, La Touche R. Clinical features of patients with chronic non-specific neck pain per disability level: A novel observational study. Rev Assoc Med Bras (1992). 2018;64(8):700-709.

17. Dieleman JL, Cao J, Chapin A, Chen C, Li Z, Liu A, et al. US health care spending by payer and health condition, 1996‑2016. JAMA. 2020;323(9):863–84.

18. Nainate A. Factors influencing neck and shoulder pain syndrome in computer users in Charoenkrung Pracharak Hospital. Journal of Charoenkrung Pracharak Hospital. 2020;16(2):61–74.

19. Punwaree J, Klaphajone J, Kovindha A. Neck Pain in Computer Users: a study of Ergonomic Factors. J Thai Rehabil Med. 2009;19(1):30-35.

20. Kazeminasab S, Nejadghaderi SA, Amiri P, Pourfathi H, Araj-Khodaei M, Sullman MJM, Kolahi AA, Safiri S. Neck pain: global epidemiology, trends and risk factors. BMC Musculoskelet Disord. 2022;3;23(1):26.

21. Cummings TM, Baldry PE. Regional myofascial pain: diagnosis and management. Best Pract Res Clin Rheumatol. 2007;21(2):367–87.

22. Téllez EC, Lacomba MT, Mayoral OM, Sánchez BS, Dommerholt J, Ortega CG. Prevalence of Myofascial Pain Syndrome in Chronic Non-Specific Neck Pain: A Population-Based Cross-Sectional Descriptive Study. Pain Med. 2016;17(12):2369-2377.

23. Lam C, Francio VT, Gustafson K, Carroll M, York A, Chadwick AL. Myofascial pain - A major player in musculoskeletal pain. Best Pract Res Clin Rheumatol. 2024;38(1):1-13.

24. Cummings M, Baldry P. Regional myofascial pain: diagnosis and management. Best Pract Res Clin Rheumatol. 2007;21: 367-387.

25. Borg-Stein J, Iaccarino MA. Myofascial pain syndrome treatments. Phys Med Rehabil Clin 2014;25(2):357–74.

26. Affaitati G, Fabrizio A, Savini A, Lerza R, et al. A randomized, controlled study comparing a lidocaine patch, a placebo patch, and anesthetic injection for treatment of trigger points in patients with myofascial pain syndrome: evaluation of pain and somatic pain thresholds. Clin Therapeut 2009;31(4):705–20.

27. Appasamy M, Lam C, Alm J, Chadwick AL. Trigger point injections. Phys Med Rehabil. Clin 2022;33(2):307–33.

28. Rayalam K, Maneval M, Newhouser L, Varela L. Do ultrasound-guided myofascial trigger point injections lead to better outcomes and fewer complications when compared with trigger point injections without ultrasound guidance? Evidence-Based Practice 2022;25(7):18–20.

29. Finnoff JT, Hurdle MF, Smith J. Accuracy of ultrasound-guided versus fluoroscopically guided contrast-controlled piriformis injections: a cadaveric study. J Ultrasound Med 2008;27(8):1157–1163.

30. Nicolakis P, Erdogmus B, Kopf A, Nicolakis M, et al. Effectiveness of exercise therapy in patients with myofascial pain dysfunction syndrome. J Oral Rehabil. 2002;29(4):362–8.

31. Gerber LH, Shah J, Rosenberger W, Armstrong K, Turo D, Otto P, Heimur J, Thaker N, Sikdar S. Dry needling alters trigger points in the upper trapezius muscle and reduces pain in subjects with chronic myofascial pain. PM R. 2015;7(7):711-8.

32. Tough EA, White AR, Cummings TM, Richards SH, Campbell JL. Acupuncture and dry needling in the management of myofascial trigger point pain. Eur J Pain 2009;13(1):3-10.

33. Dommerholt J, Mayoral O, Gröbli C. Trigger point dry needling. Journal of Manual & Manipulative Therapy. 2006;14(4), E70–E87.

34.Liu C, Wang Y, Yu W, Xiang J, et al. Comparative effectiveness of noninvasive therapeutic interventions for myofascial pain syndrome: a network meta-analysis of randomized controlled trials. Int J Surg. 2024;110(2):1099–112.

35. Xia P, Wang X, Lin Q, Cheng K, et al. Effectiveness of ultrasound therapy for myofascial pain syndrome: a systematic review and meta-analysis. J Pain Res. 2017; 10:545–555.

36. Li X, Wang R, Xing X, Shi X, et al. Acupuncture for myofascial pain syndrome: a network meta-analysis of 33 randomized controlled trials. Pain Physician. 2017; 20(6): E883–902.

37. Borg-Stein J, Iaccarino MA. Myofascial pain syndrome treatments. Phys Med Rehabil Clin 2014;25(2):357–74.

38. Rossi S, Hallett M, Rossini PM, Pascual A, Safety of TMS Consensus Group. Safety, ethical considerations, and application guidelines for the use of transcranial magnetic stimulation in clinical practice and research. Clin Neurophysiol. 2009;120(12):2008-39.

39. Lo YL, Fook-Chong S, Huerto AP, George JM. A randomized, placebo-controlled trial of repetitive spinal magnetic stimulation in lumbosacral spondylotic pain. Pain Med. 2011;12(7):1041-10455.

40. Kim SG, Nam SM. Effects of repetitive peripheral magnetic stimulation on pain, disability, and quality of life in chronic low back pain patients. J Magnetics 2021;26(1):111–115.

41. Banyat N. Effectiveness of Peripheral Magnetic Stimulation and Dry Needling in Patients with Myofascial Pain Syndrome of Neck. Journal of Health Science. 2023;32(2): S310-S319.

42. Vearasilp A, Sukareechai C. Effectiveness of Repetitive Peripheral Magnetic Stimulation (rPMS) in Relieving Post-Needling Soreness in Patient with Upper Trapezius Myofascial Pain Syndrome: A Double-Blind, Randomized Clinical Trial. J Pain Res. 2025;20(18):2541-2548.

43.Jiravichitchai T, Khamket C, Ruthiraphong, P. Short-Term Efficacy of Repetitive Peripheral Magnetic Stimulation for Chronic Low Back Pain: A Double-Blinded Randomized Control Trial. Journal of the Medical Association of Thailand. 2024;107(11),894-901.

44. Hopewell S, Chan AW, Collins GS, Hróbjartsson A, Moher D, Schulz KF, et al. CONSORT 2025 statement: updated guideline for reporting randomized trials. BMJ. 2025;388: e081123.

45. Simons DG, Travell JG, Simons LS. Trapezius muscle. In: Johnson E, editor. Travell & Simons’ Myofascial pain and dysfunction: the trigger point manual. Vol. 1, Upper half of body. 2nd ed. Baltimore: Williams & Wilkins; 1998. p 278-307.

46. Borm GF, Fransen J, Lemmens WA. A simple sample size formula for analysis of covariance in randomized clinical trials. J Clin Epidemiol. 2007;60(12):1234-8.

47. Walters SJ, Jacques RM, Dos Anjos Henriques-Cadby IB, Candlish J, Totton N, Xian MTS. Sample size estimation for randomized controlled trials with repeated assessment of patient-reported outcomes: what correlation between baseline and follow-up outcomes should we assume? Trials. 2019;20(566):1-12.

48.Diz JBM, de Souza JRLM, Leopoldino AAO, Oliveira VC. Exercise, especially combined stretching and strengthening exercise, reduces myofascial pain: a systematic review. J Physiother. 2017;63(1):17–22.

49. Ostelo RW, de Vet HC. Clinically important outcomes in low back pain. Best Pract Res Clin Rheumatol 2005; 19(4):593-607.

50.Boonstra A, Schiphorst-Preuper H, Reneman M, Posthumus J, Stewart R. Reliability and validity of the visual analogue scale for disability in patients with chronic musculoskeletal pain. Int. J. Rehabil. Res. 2008;31(2), 165–169.

51. Fisher AA. Pressure algometer over normal muscle standard values validity and reducibility of pressure threshold. Pain. 1997;30(1):116-26.

52. Thongbuang S, Chatchawan U, Eungpinichpong W, Manimmanakorn N. Validity and reliability of measuring pressure pain threshold using manual algometer in subjects with trigger points on upper trapezius muscle. J Med Tech Phy Ther. 2014;26(3):313-320.

53. Uthaikhup S, Paungmali A, Pirunsan U. Validation of Thai versions of the Neck Disability Index and Neck Pain and Disability Scale in patients with neck pain. Spine. 2011;36(21):1415-1421.

54. Vernon H, Mior S. The Neck DisabilityI ndex: a study of reliability and validity. J Manipulative Physiol Ther 1991;14(7):409-15.

55. Luksanapruksa P, Wathana-apisit T,Wanasinthop S, Sanpakit S, Chavasiri C. Reliability and validity study of a Thai version of the Neck Disability Index inpatients with neck pain. J Med Assoc Thai. 2012;95(5):681-8.

**ภาคผนวก ตัวอย่าง Register Protocol** จะลงทะเบียนหลังได้ ได้รับการอนุมัติจากคณะกรรมการจริยธรรม

| **Title** |  |  |  |
| --- | --- | --- | --- |
| Public title: |  |  |  |
| Acronym: |  |  |  |
| Scientific Title: |  |  |  |
| Registration Site: |  |  |  |
| URL: |  |  |  |
| Secondary ID: |  |  |  |
| **Ethic Review** |  |  |  |
| 1. Board Approval: |  |  |  |
| Approval Number: |  |  |  |
| Date of Approval: |  |  |  |
| Board Name: |  |  |  |
| Board Certification: |  |  |  |
| Board Contact: | Business Phone: |  |  |
|  | Business Email: |  |  |
|  | Email: |  |  |
| **Sponsor** |  |  |  |
| Source(s) of Monetary or Material Supports: |  |  |  |
| Study Primary Sponsor: |  |  |  |
| Responsibility Party: | Name/Official Title: |  |  |
|  | Organization: |  |  |
|  | Phone: |  |  |
|  | Email: |  |  |
| Study Secondary Sponsor: |  |  |  |
| **Protocol Synopsis** |  |  |  |
| Protocol Synopsis: | Objectives |  |  |
|  | Primary: |  |  |
|  | Secondary: |  |  |
|  | Methods |  |  |
| **URL not available** |  |  |  |
| **Health Conditions** |  |  |  |
| Health Condition(s) or Problem(s) Studied: |  |  |  |
| Keywords: |  |  |  |
| **Eligibility** |  |  |  |
| Inclusion criteria: |  |  |  |
|  |  |  |  |
|  |  |  |  |
|  |  |  |  |
|  |  |  |  |
|  |  |  |  |
| Gender: | Both |  |  |
| Age Limit: |  |  |  |
| Exclusion Criteria: |  |  |  |
|  |  |  |  |
|  |  |  |  |
|  |  |  |  |
|  |  |  |  |
|  |  |  |  |
|  |  |  |  |
|  |  |  |  |
| Accept Healthy Volunteers: |  |  |  |
| **Status** |  |  |  |
| Overall Recruitment Status: |  |  |  |
| Key Trial Dates: | Study Start Date (First enrollment): | Indicate Type: Actual |  |
|  | Completion Date (Last Subject, Last visit): | Indicate Type: Actual |  |
|  | Study Completion Date: | Indicate Type: Actual |  |
| **Design** |  |  |  |
| Study Type: |  |  |  |
| Primary Purpose: |  |  |  |
| Intervention Model: |  |  |  |
| Number of Arms: |  |  |  |
| Masking: |  |  |  |
| Allocation: |  |  |  |
| Controlled: |  |  |  |
| Study Endpoint Classification: |  |  |  |
| Sample size: |  |  |  |
|  | Actual sample size at study completion: |  |  |
| Intervention Arm 1: |  |  |  |
|  | Intervention name: |  |  |
|  | Intervention Type: |  |  |
|  | Intervention Classification: Device |  |  |
|  | Intervention Description: |  |  |
| Intervention Arm 2: | Intervention name: |  |  |
|  | Intervention Type: |  |  |
|  | Intervention Classification: Device |  |  |
|  | Intervention Description: |  |  |
| **Outcome** |  |  |  |
| Primary outcome |  |  |  |
| 1. Outcome name |  |  |  |
| Metric/Method of measurement: |  |  |  |
| Time point: |  |  |  |
| Secondary outcome |  |  |  |
| 1. Outcome name |  |  |  |
| Metric/Method of measurement: |  |  |  |
| Time point: |  |  |  |
| 2. Outcome name |  |  |  |
| Metric/Method of measurement: |  |  |  |
| Time point: |  |  |  |
| **Location** |  |  |  |
| **Section A: Central Contact** |  |  |  |
| Central Contact | First Name: | Middle Name: | Last Name: |
|  | Degree: | Phones: | Email: |
| Central Contact Backup | First Name: | Middle Name: | Last Name: |
|  | Degree: | Phones: | Email: |
| **Section B Facility Information and Contact** |  |  |  |
| 1. | Site Name: | State/Provinces: | Postal Code: |
|  | City: | Recruitment Status: |  |
|  | Country: |  |  |
| Facility Contact | First Name: | Middle Name: | Last Name: |
|  | Degree: | Phones: | Email: |
| Facility Contact Backup | First Name: | Middle Name: | Last Name: |
|  | Degree: | Phones: | Email: |
| Investigator Name | First Name: Thitirut | Middle Name: | Last Name: |
|  | Degree: | Phones: | Email: |
| **Section C Contact for Public Queries (Responsible Person)** | First Name: | Middle Name: | Last Name: |
|  | Degree: | Phones: | Email: |
|  | Postal Address: |  |  |
|  | State/Province: | Postal code: |  |
|  | Country: Thailand | Official Role: Study Principal Investigator |  |
|  | Organization Affiliation: |  |  |
|  | First Name: | Middle Name: | Last Name: |
|  | Degree: | Phones: | Email: |
|  | Postal Address |  |  |
|  | State/Province: | Postal code: |  |
|  | Country: | Official Role: |  |
|  | Organization Affiliation: |  |  |
| **Summary Results** |  |  |  |
| Date of posting results summaries: |  |  |  |
| Date of first journal publication of results: |  |  |  |
| **Deidentified Individual Participant-Level Data Sharing** |  |  |  |
| Plan to share IPD: |  |  |  |
| Reason: |  |  |  |
| Publication from this study |  |  |  |
| MEDLINE Identifier: |  |  |  |
| URL link to full text publication: |  |  |  |
