# Supplementary material for: Comparison of two repetitive peripheral magnetic stimulation protocols for upper trapezius myofascial pain syndrome: A randomized trial
Source: PLoS One. 2026 Oct 8;21(10):e0360355. doi: 10.1371/journal.pone.0360355 (PMC13648980; doi:10.1371/journal.pone.0360355)
Supplement: Supplementary file 4 [file pone.0360355.s005.docx]

**Dear Editor and Reviewers**

Many thanks for your detailed and constructive comments on our manuscript. We are pleased to provide a point-by-point response below. Changes in the revised manuscript are highlighted.

With best wishes.

Yours faithfully

The authors

**Reviewer #1**

This manuscript presents a randomized controlled trial comparing two repetitive peripheral magnetic stimulation (rPMS) protocols (10-minute versus 15-minute sessions) for upper trapezius myofascial pain syndrome (MPS). The topic is clinically relevant, and the study addresses an important practical question regarding treatment parameter optimization. The manuscript is generally well organized, clearly written, and methodologically stronger than many small rehabilitation trials. The use of longitudinal mixed-effects modeling, assessor blinding, and intention-to-treat analysis are notable strengths.

However, several methodological and statistical concerns require clarification and revision before the manuscript can be considered for publication. In particular, the interpretation of non-significant findings as evidence of comparable efficacy is not fully supported by the study design or statistical framework. In addition, the data availability statement appears inconsistent with the materials currently provided.

**Major Comments**

**1.) Interpretation of Non-Significant Findings and Equivalence Claims**

The principal concern relates to the interpretation of the results. The manuscript repeatedly implies that the 10-minute protocol is “comparable” or “equally effective” relative to the 15-minute protocol. However, the trial appears to have been designed and analyzed as a superiority study rather than an equivalence or noninferiority trial.

A non-significant protocol-by-time interaction does not establish equivalence between interventions. Demonstrating equivalence or noninferiority would require:

a prespecified equivalence/noninferiority margin,

dedicated sample size justification,

and appropriate equivalence/noninferiority statistical methodology.

At present, the findings support only the conclusion that no statistically significant difference was detected between protocols within the study sample and follow-up period.

**RESPONSE:**

We sincerely thank the reviewer for this important methodological observation. We fully agree that the present study was designed and analyzed as a superiority trial and not as an equivalence or noninferiority trial. Therefore, the absence of a statistically significant group-by-time interaction cannot be interpreted as evidence of equivalence between treatment protocols.

In response to this comment, we carefully revised the Abstract, Results, Discussion, and Conclusions sections to remove language implying equivalence, comparable efficacy, or equal effectiveness. The revised manuscript now states more conservatively that no statistically significant between-protocol differences were detected within the present sample and follow-up period.

We additionally expanded the Limitations section to explicitly acknowledge that the study was not designed or powered as an equivalence or noninferiority trial and did not prespecify an equivalence or noninferiority margin. Accordingly, the findings should not be interpreted as formal evidence of equivalence between treatment durations.

**Changes made in the revised manuscript:**

**Abstract**

**Conclusions section**

**Old version:** “...indicating comparable efficacy between protocols.” (Page 2, Lines 57)

**New version** (Page 2, Lines 54-55)**:** “No statistically significant between-protocol differences were detected within the present sample and follow-up period.”

**Methods**

**Study Design**

**Additional text added** (Page 6, Lines 137-142)**:**

“The trial adopted a superiority framework to determine whether extending rPMS session duration from 10 minutes to 15 minutes would result in superior short-term clinical outcomes. Because previous sham-controlled studies have suggested short-term benefits of rPMS for upper trapezius MPS [27],… ”

**Strength and Limitations**

**Additional text added** (Page 19, Lines 523-526)**:**

“Finally, the study was not designed or powered as an equivalence or noninferiority trial and did not prespecify an equivalence or noninferiority margin. Therefore, the absence of statistically significant between-group differences should not be interpreted as formal evidence of equivalence between treatment durations [52].”

**Conclusions**

**Old version:** “...indicating comparable efficacy between the two protocols.” (Page 17, Lines 477-479)

**New version** (Page 19, Lines 530-533)**:** “No statistically significant between-protocol differences were detected within the present sample and follow-up period. These findings suggest that a shorter 10-minute rPMS protocol may represent a practical treatment option in clinical rehabilitation settings.”

**2.) Sample Size Justification Requires Clarification**

The sample size calculation assumes a clinically meaningful between-group VAS difference of 0.5 points with SD = 0.8. Please provide stronger clinical justification for selecting a 0.5-point difference as the target effect size, as this appears smaller than commonly accepted minimal clinically important differences for neck pain outcomes.

Additionally, the manuscript states that sample size estimation was based on an ANCOVA framework, whereas the primary analysis used longitudinal mixed-effects modeling. Please clarify:

whether repeated-measures correlation was incorporated appropriately,

and whether the final analysis framework was considered during power estimation.

RESPONSE:

We sincerely thank the reviewer for this important comment regarding the sample size estimation and analytical framework. In response, we expanded the Sample Size subsection of the Methods section to clarify both the rationale for the selected target effect size and the relationship between the sample size estimation approach and the final longitudinal analytical framework.

First, we clarified that the assumed between-group difference of 0.5 points on the VAS was not intended to represent a minimal clinically important difference (MCID). Rather, it was selected as a conservative design assumption for detecting a small incremental treatment effect between two active rPMS duration protocols. Because both groups received active rPMS combined with standardized stretching exercises, only a modest between-group difference was anticipated. Furthermore, no previous randomized trial had directly compared different rPMS treatment durations in patients with upper trapezius myofascial pain syndrome. Therefore, the selected effect size was considered a pragmatic and conservative estimate for study planning purposes rather than an established treatment effect.

Second, we clarified that repeated-measures correlation was incorporated into the sample size estimation through the assumption of a baseline-to-follow-up correlation coefficient of 0.6, consistent with published recommendations for musculoskeletal patient-reported outcomes. This assumption was included within the ANCOVA-based framework to account for within-subject correlation and to improve the efficiency of the sample size calculation.

Third, we clarified the relationship between the sample size estimation and the final analytical approach. Although the primary analyses were subsequently performed using restricted maximum likelihood (REML)-based linear mixed-effects models (LMMs), the ANCOVA-based sample size approach was considered appropriate because both methods account for baseline measurements and within-subject correlation when analyzing longitudinal continuous outcomes.

**Changes made in the revised manuscript:**

**Method**

**Sample size**

**Old version:** “A clinically meaningful between-group difference of 0.5 points was assumed based on prior rPMS studies.” (Page 7, Lines 175-176)

**New version** (Page 7, Lines 176-183)**:**

“A between-group difference of 0.5 points on the VAS was selected as a conservative design assumption for detecting a small incremental treatment effect between two active rPMS duration protocols rather than as a minimal clinically important difference (MCID). This assumption was informed by previous rPMS studies conducted in musculoskeletal pain populations, including chronic low back pain [29], which suggested that greater stimulation exposure might provide additional clinical benefit. However, no previous randomized trial had directly compared different rPMS treatment durations in patients with upper trapezius myofascial pain syndrome. Therefore, the assumed between-group difference was considered a pragmatic and conservative estimate for study planning purposes rather than an established treatment effect.”

**Additional text added** (Page 7, Lines 183-184)**:**

**“**Because both groups received active rPMS combined with standardized stretching exercises, only a modest between-group difference was expected.”

**Old version** (Page 7, Lines 183-185)**:** “…with a common standard deviation of 0.8. A baseline-to-follow-up correlation coefficient of 0.6 was assumed, consistent with values reported for musculoskeletal patient-reported outcomes by Walters et al.”

**New version** (Page 7, Lines 184-187)**:**

“The assumed standard deviation of 0.8 was based on variability estimates reported in previous rPMS studies [28]. A baseline-to-follow-up correlation coefficient of 0.6 was assumed, consistent with values reported for musculoskeletal patient-reported outcomes by Walters et al. [33].”

**Additional text added** (Page 7, Lines 190-193)**:**

“Although the primary analyses were performed using linear mixed-effects models (LMMs) with restricted maximum likelihood (REML) estimation, the ANCOVA-based sample size approach was considered appropriate because both methods account for baseline measurements and within-subject correlation when analyzing longitudinal continuous outcomes.”

**3.) Lack of Sham or Usual-Care Control Group**

The absence of a sham-control or exercise-only comparator substantially limits interpretation of treatment efficacy. Both groups received active rPMS plus stretching exercises; therefore, it is not possible to determine:

the magnitude of the specific rPMS effect,

the contribution of stretching,

or the role of contextual/placebo effects.

Although this limitation is acknowledged, it should be emphasized more clearly throughout the Discussion and Conclusions. The study should be framed primarily as a comparison of two active protocols rather than evidence confirming efficacy of rPMS itself.

RESPONSE:

We sincerely thank the reviewer for this important comment. We fully agree that the lack of a sham-controlled or usual-care comparison group limits interpretation of the specific therapeutic contribution of rPMS and restricts the ability to distinguish treatment-specific effects from the effects of concomitant stretching exercises and potential contextual or placebo-related influences.

In response, we revised the Discussion, Limitations, and Conclusions sections to more clearly acknowledge these methodological limitations and to ensure that the study is consistently framed as a comparison between two active rPMS treatment-duration protocols rather than as evidence confirming the efficacy of rPMS itself.

Specifically, we clarified that because both groups received active rPMS in combination with standardized stretching exercises, the present study design does not permit determination of: (1) the magnitude of the specific rPMS effect, (2) the independent contribution of stretching exercises, or (3) the potential influence of contextual or placebo-related effects. We further emphasized that the primary objective of the study was to compare short-term clinical outcomes between two active treatment durations under pragmatic rehabilitation conditions.

To improve interpretive precision, we revised statements throughout the manuscript that could potentially be interpreted as implying confirmation of rPMS efficacy. The revised manuscript now consistently states that no statistically significant between-protocol differences were detected within the present sample and follow-up period and emphasizes that the findings should be interpreted within the context of an active-comparator trial design.

**Changes made in the revised manuscript:**

**Discussion – Overall Findings**

**Old version:** “...supporting the safety and tolerability of the intervention under the applied stimulation parameters.” (Page 15, Lines 412)

**New version:**

**Additional text added** (Page 17, Lines 428-430)**:**

“Importantly, because both groups received active rPMS combined with standardized stretching exercises, the present findings should be interpreted as a comparison between two active treatment protocols rather than as evidence confirming the efficacy of rPMS itself.”

**Strength and Limitations**

**Old version:** “Consequently, the absence of a sham arm limits the ability to fully distinguish specific neuromodulatory effects from nonspecific therapeutic or contextual influences.” (Page 18, Lines 496-497)

**New version** (Page 18, Lines 512-515)**:**

“Because both groups received active rPMS in combination with standardized stretching exercises, the independent contribution of rPMS to the observed clinical improvements could not be determined. Consequently, the specific effects of rPMS cannot be distinguished from the effects of stretching exercises or potential contextual and placebo-related influences.”

**Conclusions**

**Old version:** “Both 15-minute and 10-minute rPMS protocols produced…” (Page 17, Lines 475)

**New version** (Page 19, Lines 529-531)**:**

“Both treatment durations were associated with significant short-term improvements in pain intensity, pressure pain threshold, and neck-related disability in individuals with upper trapezius MPS. No statistically significant between-protocol differences were detected within the present sample and follow-up period.”

**4.) Limited Separation Between Intervention Arms**

The biological distinction between the two protocols may be insufficient to meaningfully evaluate dose-response effects. Both groups received the identical 5-minute targeted trigger-point stimulation phase, while only the scanning phase duration differed.

Please discuss more explicitly whether the focused trigger-point stimulation phase may represent the primary active component of the intervention, thereby limiting the ability to detect duration-dependent effects.

RESPONSE:

We thank the reviewer for this insightful observation. We agree that the biological distinction between the two intervention protocols may have been insufficient to fully evaluate duration-dependent dose-response effects. Although the total treatment durations differed (15 versus 10 minutes), both protocols incorporated an identical 5-minute focused trigger-point stimulation phase delivered directly over the active trigger point using the same stimulation parameters.

In response to the reviewer’s suggestion, we expanded the Discussion section to more explicitly acknowledge that the shared trigger-point stimulation phase may have represented the principal therapeutic component of the intervention. Consequently, despite differences in total treatment duration, the biological contrast between the two protocols may have been relatively modest, thereby limiting the ability of the present study to detect duration-dependent effects.

We further discussed the possibility that effective engagement of sensitized trigger-point tissue may be more important than extending the duration of the surrounding scanning phase. Under this interpretation, the focused trigger-point stimulation phase may have provided the majority of the therapeutic benefit in both groups, whereas prolonging the scanning component may have contributed relatively little additional clinical effect.

Because the present study was not specifically designed to evaluate mechanistic pathways or dose-response relationships, these explanations remain speculative. Nevertheless, we agree that this represents a plausible interpretation of the findings and an important consideration for future studies investigating optimal stimulation parameters and dose-response effects of rPMS.

**Changes made in the revised manuscript:**

**Discussion**

**Old version:** “If peripheral desensitization is achieved primarily during this focused phase, extending the surrounding scanning component may provide limited additional suppression of nociceptive signaling at the sensitized locus.” (Page 16, Lines 435-436)

**New version:**

**Additional text added** (Page 18, Lines 456-459)**:**

“Consequently, the biological distinction between the two treatment protocols may have been relatively modest despite the difference in total treatment duration. The shared trigger-point stimulation phase may therefore have represented the principal therapeutic component of the intervention, potentially limiting the ability of the present study to detect duration-dependent dose-response effects.”

**5.) Missing Data Handling Requires Additional Transparency**

The use of REML-based mixed models and MICE sensitivity analyses is appropriate. However, additional information regarding missingness is needed.

Please provide:

the number of missing observations by group and timepoint,

reasons for missingness,

and clarification regarding the plausibility of the MAR assumption.

It would also strengthen the manuscript to provide supplementary information regarding the imputation procedure and variables included in the MICE model.

RESPONSE:

We thank the reviewer for this valuable comment. In response, we revised the manuscript to provide further details regarding the pattern of missingness, the rationale supporting the missing-at-random (MAR) assumption, and the multiple imputation procedure.

**1. Number of missing observations by group and assessment time point**

Additional information regarding missing data has been added to the Results section and Supplementary Table S1. Outcome data were complete for all participants at baseline. Missing assessments occurred in 2 participants (3.0 %) at week 2 and 12 participants (18.2 %) at week 4. Missingness was balanced across treatment groups, with one participant per group missing the week 2 assessment and six participants per group missing the week 4 assessment. (Table S1).

**Table S1**. Missing observations by treatment group and assessment time point

| **Assessment time point** | **Protocol A (15 min)** | **Protocol B (10 min)** |
| --- | --- | --- |
| Baseline | 0 | 0 |
| Week 2 | 1 | 1 |
| Week 4 | 6 | 6 |

**2. Reasons for missingness**

We have also clarified the reasons for missingness in the revised manuscript. Missing assessments were attributable to inability to continue treatment (n = 5), severe flooding (n = 4), influenza A infection (n = 1), transportation difficulties (n = 1), and a motor vehicle accident (n = 1). Importantly, no participant withdrew because of treatment-related adverse events or lack of treatment efficacy.

**3. Plausibility of the missing-at-random (MAR) assumption**

Additional clarification regarding the MAR assumption has been added to the Statistical Analysis section. Based on review of participant records, missingness appeared to be attributable primarily to external and logistical factors rather than treatment response or clinical outcomes. Furthermore, missing assessments were balanced across treatment groups, and no withdrawals were related to treatment efficacy or adverse events. Baseline demographic and clinical variables were available for all participants and were incorporated into both the analytical and imputation models. Taken together, these considerations support the plausibility of the MAR assumption underlying the primary restricted maximum likelihood (REML)-based mixed-effects analyses.

**4. Multiple imputation procedure**

To further improve methodological transparency, we expanded the description of the multiple imputation procedure. Sensitivity analyses were performed using multiple imputation by chained equations (MICE), with 20 imputed datasets generated using linear regression models. The imputation model included treatment group, baseline demographic variables (age, sex, and body mass index), baseline outcome measures, and all available follow-up outcome measurements (VAS, PPT, and NDI). Parameter estimates were pooled according to Rubin’s rules. Results from the MICE sensitivity analyses were consistent with those obtained from the primary REML-based mixed-effects models, with no substantial changes in effect estimates, statistical significance, or study conclusions, supporting the robustness of the findings.

**Changes made in the revised manuscript:**

**Statistical Analysis**

**Old version:** “To evaluate robustness, sensitivity analyses were conducted using multiple imputation by chained equations (MICE). Twenty imputed datasets (m = 20) were generated using linear regression models, and parameter estimates were pooled according to Rubin’s rules. Sensitivity analyses yielded results consistent with the primary REML models; specifically, the protocol-by-time interaction remained non-significant across all outcomes.” (Page 10, Lines 284-288)

**New version** (Page 11, Lines 301-310)**:**

“To evaluate robustness, sensitivity analyses were conducted using multiple imputation by chained equations (MICE). Twenty imputed datasets (m = 20) were generated using linear regression models. The imputation model included treatment group, baseline demographic variables (age, sex, and body mass index), baseline outcome measures, and all available follow-up outcome measurements (VAS, PPT, and NDI). Parameter estimates were pooled according to Rubin’s rules. The missing-at-random (MAR) assumption was considered plausible because missing data were primarily attributable to external and logistical factors rather than treatment response or clinical outcomes. In addition, missing assessments were balanced across treatment groups, and baseline demographic and clinical variables were available for all participants and incorporated into both the analytical and imputation models. Sensitivity analyses yielded results consistent with the primary analyses; specifically, the group-by-time interaction remained non-significant across all outcomes.”

**Results – Participants Flow**

**New version:**

**Additional text added:**

“Missing assessments occurred in 2 participants (3.0 %) at week 2 and 12 participants (18.2 %) at week 4 and were balanced across treatment groups.” (Page 11, Lines 308-309)

**Additional text added** (Page 13, Lines 331-333)**:**

“Missing assessments were primarily attributable to inability to continue treatment, severe flooding, influenza A infection, transportation difficulties, and a motor vehicle accident. No participant withdrew because of treatment-related adverse events.”

**6.) Data Availability Statement Appears Incomplete**

The manuscript states:

“All relevant data are within the manuscript and its Supporting Information files.”

However, no participant-level dataset, statistical code, or supplementary data files appear to be included in the submitted materials currently available for review.

Please ensure compliance with the journal’s data availability requirements by providing:

a de-identified participant-level dataset,

variable definitions/data dictionary,

and preferably the statistical analysis code or syntax.

If these materials are available elsewhere, please provide the repository link or accession information

RESPONSE:

Thank you for this feedback. We have provided a link to the de-identified participant-level dataset. The dataset includes variable definitions to facilitate interpretation and reuse of the data.

The repository link has now been included in the manuscript as follows:

**Data Availability Statement**

All relevant data underlying the results reported in this study are publicly available in the Open Science Framework (OSF) repository (DOI: 10.17605/OSF.IO/87P5W).

**Minor Comments**

**1.) Clarify Blinding Limitations**

The study is described as assessor-blinded; however, participants were likely aware of treatment duration differences. Please discuss the potential for performance and expectation bias more explicitly.

RESPONSE:

We thank the reviewer for this helpful observation. We agree that although outcome assessors remained blinded throughout the study, participants were likely aware of differences in treatment duration between the two intervention protocols. Consequently, the potential for performance bias and expectation-related effects cannot be completely excluded.

In response, we expanded the Limitations section to more explicitly acknowledge this issue and its potential influence on study outcomes. Specifically, we now discuss the possibility that participants may have perceived longer treatment sessions as potentially more effective, which could have influenced subjective outcome reporting. We also clarify that the inclusion of both subjective (VAS and NDI) and objective (PPT) outcome measures may partially mitigate concerns regarding expectation-related bias.

**Changes made in the revised manuscript:**

**Strength and Limitations**

**New version:**

**Additional text added** (Page 18, Lines 516-519)**:**

“Although outcome assessors remained blinded, participant blinding was not feasible because treatment duration differed between protocols, introducing the possibility of expectation-related bias. However, the inclusion of both subjective (VAS and NDI) and objective (PPT) outcomes may partially mitigate this concern.”

**2.) Adverse Event Reporting**

The manuscript reports that no serious adverse events occurred, but it is unclear whether minor or transient adverse effects were monitored systematically.

Please clarify whether participants experienced:

discomfort,

muscle soreness,

headaches,

skin sensations,

or treatment intolerance.

RESPONSE:

We thank the reviewer for this important comment regarding adverse event reporting.

Participants were monitored throughout the intervention period for treatment intolerance and adverse events. No serious adverse events occurred, and no participants discontinued treatment because of adverse effects.

To improve transparency, we have clarified this limitation in the revised manuscript and now explicitly state that no serious adverse events or treatment-related discontinuations were observed during the study period.

**Changes made in the revised manuscript:**

**Methods**

**New version:**

**Additional text added** (Page 9, Lines 235-238)**:**

“**Adverse Event Monitoring**

“Participants were routinely asked about treatment-related symptoms during each treatment session, including discomfort during stimulation, muscle soreness, headache, abnormal skin sensations, dizziness and treatment intolerance.”

**Results – Participants Flow**

**New version:**

**Additional text added** (Page 12, Lines 336-339)**:**

**“**Participants were monitored for adverse events throughout the intervention period. No serious adverse events or treatment-related discontinuations occurred. No clinically significant treatment-related adverse effects requiring medical attention, treatment modification, or study withdrawal were reported.”

**3.) Multiplicity of Statistical Testing**

Multiple outcomes and repeated contrasts were analyzed without mention of multiplicity adjustment. While this may be acceptable for exploratory secondary analyses, the manuscript should clarify:

which endpoint was prespecified as primary,

and whether secondary analyses should be interpreted cautiously.

RESPONSE:

We thank the reviewer for this important comment. In response, we expanded the Statistical Analysis section to more clearly distinguish between the prespecified primary and secondary outcomes and to clarify our approach to multiplicity. Specifically, pain intensity measured using the VAS is prespecified as the primary outcome and served as the basis for sample size estimation. PPT and NDI are prespecified as secondary outcomes.

Because the primary hypothesis concerned the VAS outcome, no formal adjustment for multiplicity was applied. However, to improve transparency and avoid overinterpretation, we explicitly state in the revised manuscript that findings relating to secondary outcomes and exploratory contrasts should be interpreted cautiously.

**Changes made in the revised manuscript:**

**Statistical Analysis**

**New version:**

**Additional text added** (Page 10, Lines 291-294)**:**

“Pain intensity measured by the VAS was prespecified as the primary outcome and served as the basis for sample size estimation. PPT and NDI were prespecified as secondary outcomes. Because the primary hypothesis concerned the VAS outcome, no formal adjustment for multiplicity was applied.”

**4.) Mechanistic Interpretation Should Be Moderated**

Several sections of the Introduction and Discussion contain extensive mechanistic interpretation regarding:

neuroplasticity,

cortical excitability,

central sensitization,

and descending inhibitory modulation.

These concepts are scientifically plausible but were not directly measured in this study. Please ensure that mechanistic statements remain appropriately speculative and hypothesis-generating.

RESPONSE:

We thank the reviewer for this thoughtful comment. We agree that mechanisms such as neuroplasticity, cortical excitability modulation, central sensitization, and descending inhibitory pathways were not directly assessed in the present study and therefore cannot be confirmed by our findings.

Our intention in discussing these mechanisms was to provide biological context for the rationale underlying the intervention design and the hypothesis that extending the scanning-phase duration might influence treatment response. However, we agree that mechanistic interpretations should remain appropriately speculative and should not be presented as explanations directly supported by the study data.

In response to the reviewer’s suggestion, we carefully revised the Introduction and Discussion sections to moderate mechanistic statements and ensure that they are presented as biologically plausible hypotheses rather than established mechanisms. Specifically, definitive language was replaced with more cautious wording. We also clarified that these mechanisms were not directly measured in the present study and that any mechanistic interpretation should therefore be considered hypothesis-generating. Additional clarification was incorporated throughout the Discussion to ensure that references to neuroplasticity, central sensitization, cortical excitability, and descending inhibitory modulation are framed as plausible biological hypotheses rather than mechanisms established by the present study.

**Changes made in the revised manuscript:**

**Introduction**

**Old version:** “Neurophysiological evidence further indicates that peripheral magnetic stimulation can alter spinal excitability and enhance sensorimotor integration, consistent with activity-dependent plasticity mechanisms.” (Page 5, Lines 112-114)

**New version** (Page 5, Lines 103-106)**:**

“Mechanistically, rPMS may modulate pain processing through preferential recruitment of large-diameter afferent fibres, inhibition of nociceptive transmission, facilitation of descending inhibitory pathways, and modulation of cortical and spinal excitability, consistent with activity-dependent neuroplasticity mechanisms [21-23].”

**Old version:** “Neuroplastic modulation is considered dose-dependent, with the magnitude and persistence of activity-dependent changes influenced by cumulative afferent input and total stimulation exposure.” (Page 5, Lines 124-125)

**New version** (Page 5, Lines 115-118)**:**

“It has been hypothesized that neuromodulatory effects may be influenced by cumulative afferent input and total stimulation exposure, although the relationship between stimulation duration and clinical response remains incompletely understood.”

**5.) Writing Style and Conciseness**

The manuscript is generally well written and uses standard academic English appropriately. However, portions of the Introduction and Discussion are overly dense and could be streamlined for clarity and conciseness.

In particular:

mechanistic background sections are somewhat lengthy relative to the study question,

several concepts are repeated, and some sentences could be shortened for readability.

A moderate reduction in length would improve accessibility without compromising scientific content.

RESPONSE:

We thank the reviewer for this constructive comment. In response, we carefully reviewed the manuscript and revised multiple sections to improve conciseness while preserving the scientific rationale and contextual background relevant to the study. Specifically, redundant descriptions of proposed neurophysiological mechanisms were reduced, overlapping concepts were consolidated, and several sentences were shortened or restructured to improve clarity and flow. Particular attention was given to the mechanistic background sections in the Introduction and the explanatory portions of the Discussion, where repetitive concepts relating to neuroplasticity, cortical excitability, and neuromodulatory effects were simplified.

These revisions resulted in a more focused presentation of the study rationale and findings while maintaining the scientific context necessary for interpretation of the results.

**Overall Recommendation**

The manuscript addresses a clinically relevant question and demonstrates several methodological strengths, including randomized allocation, blinded assessment, and appropriate longitudinal modeling. However, revisions are needed to:

avoid equivalence overinterpretation,

improve statistical transparency,

clarify data availability,

and moderate mechanistic claims.

With appropriate revision, the study may provide a useful contribution to the rehabilitation and musculoskeletal pain literature.

RESPONSE:

We sincerely thank the reviewer for the thorough, constructive, and thoughtful evaluation of our manuscript. In response to the reviewer’s comments, we carefully revised the manuscript to address each concern.

We believe that these revisions have substantially improved the methodological transparency, interpretive precision, and overall clarity of the manuscript. We are grateful for the reviewer’s insightful suggestions and believe they have significantly strengthened the quality of the work.

Once again, we thank the reviewer for the time and expertise devoted to evaluating our study.

**Reviewer #2**

**GENERAL COMMENTS**

I appreciate the opportunity to review manuscript #PONE-D-26-17312 titled "Effect of two repetitive peripheral magnetic stimulation protocols on upper trapezius myofascial pain syndrome: a randomized controlled trial". I have several comments and suggestions, detailed in my specific comments that follow, that I believe will strengthen this work.

**SPECIFIC COMMENTS**

***Page 1, lines 2 and 3 (Title)** – Since there was no true control group, please replace “randomized controlled trial” with “randomized trial” here and throughout the rest of the manuscript.

RESPONSE:

We thank the reviewer for this helpful comment. To avoid potential ambiguity regarding the nature of the comparator arm, we have revised the title and corresponding references throughout the manuscript by replacing “randomized controlled trial” with “randomized trial.”

**Changes made in the revised manuscript:**

**Title**

**Old version:** “Effect of two repetitive peripheral magnetic stimulation protocols on upper trapezius myofascial pain syndrome: a randomized controlled trial” (Page 1, Lines 1-3)

**New version** (Page 1, Lines 1-3)**:**

“Comparison of Two Repetitive Peripheral Magnetic Stimulation Protocols for Upper Trapezius Myofascial Pain Syndrome: A Randomized Trial”

***Page 2, line 45 (Abstract, Results)** – Suggest you replace “per protocol” with “per group”.

RESPONSE:

We thank the reviewer for this helpful suggestion. Accordingly, we revised the wording throughout the manuscript by replacing “per protocol” with “per group” or equivalent group-based descriptions where appropriate. This change was made to improve clarity and ensure consistency with standard CONSORT reporting terminology.

**Changes made in the revised manuscript:**

**Throughout the manuscript**

**For example;**

**Abstract – Results section**

**Old version:** “Of 83 individuals screened, 66 were randomized (33 per protocol) and included in the ITT analysis.” (Page 2, Lines 45-46)

**New version** (Page 2, Lines 44)**:**

“Of 83 individuals screened, 66 were randomized (33 per group) and included in the ITT analysis.”

***Page 6, line 1 (Methods, Study Design)** – What was the rationale for not including a true control group that received no intervention or some type of sham intervention?

RESPONSE:

We thank the reviewer for this important question regarding the study design.

The primary objective of the present study was not to establish the efficacy of rPMS compared with no treatment or sham stimulation, but rather to determine whether extending treatment duration from 10 minutes to 15 minutes would provide additional clinical benefit when applied under routine rehabilitation conditions. Accordingly, the study was designed as an active-comparator randomized trial comparing two clinically applicable rPMS protocols rather than an efficacy trial evaluating rPMS against a true control condition.

At the time of study design, previous studies had already suggested that rPMS provides beneficial effects in patients with myofascial pain syndrome and other musculoskeletal pain conditions, including evidence from sham-controlled investigations. Therefore, our research question focused on treatment optimization rather than initial efficacy determination. Specifically, we sought to evaluate whether a longer stimulation duration would enhance clinical outcomes sufficiently to justify the additional treatment time and resource utilization required in routine clinical practice.

We agree, however, that the absence of a sham-control or no-treatment comparator limits the ability to determine the specific therapeutic contribution of rPMS and to distinguish treatment-specific effects from stretching-related, contextual, or placebo-related influences. This limitation has now been emphasized more explicitly in the Discussion and Limitations sections of the revised manuscript.

**Changes made in the revised manuscript:**

**Methods**

**Study design**

**New version:**

**Additional text added** (Page 6, Lines 139-142)**:**

**“**Because previous sham-controlled studies have suggested short-term benefits of rPMS for upper trapezius myofascial pain syndrome [27], the primary objective of the present study was treatment-parameter optimization rather than re-establishing efficacy against placebo. Accordingly, the trial compared two active treatment durations under pragmatic rehabilitation conditions.”

***Page 7, lines 171 through 176 (Methods, Sample size)** – First, please tell the reader if your sample size estimate was based on your primary outcome, pain intensity using the Visual Analog Scale, or something else, for example, some type of standardized effect size. Second, please clarify for the reader whether a clinically meaningful between-group difference of 0.5 points was based on the VAS or something else.

RESPONSE:

We thank the reviewer for this helpful comment. We agree that the rationale underlying the sample size estimation requires further clarification.

First, the sample size calculation was based on the prespecified primary outcome, pain intensity measured using the Visual Analog Scale (VAS), rather than on a standardized effect size. As now clarified in the revised manuscript, the VAS was designated a priori as the primary outcome and served as the basis for sample size estimation.

Second, the assumed between-group difference of 0.5 points referred specifically to the VAS pain score. This value was selected as a conservative design assumption for detecting a small incremental treatment effect between two active rPMS duration protocols. It was not a standardized effect size and was not intended to represent a minimal clinically important difference (MCID). Because both groups received active rPMS combined with standardized stretching exercises, only a modest between-group difference was anticipated.

**Changes made in the revised manuscript:**

**Methods**

**Sample size**

**Old version:** “Sample size was determined for a two-arm randomized trial using an analysis of covariance (ANCOVA) model with baseline adjustment...” (Page 7, Lines 171-172)

**New version:**

**Additional text added** (Page 7, lines 172-174)**:**

“The calculation was based on the prespecified primary outcome, pain intensity measured using the VAS, assessed at baseline and at Weeks 2 and 4.”

**Additional text added** (Page 7, lines 175-178)**:**

“A between-group difference of 0.5 points on the VAS was selected as a conservative design assumption for detecting a small incremental treatment effect between two active rPMS duration protocols rather than as a minimal clinically important difference (MCID).”

***Page 7, lines 183 through 191 (Methods, Randomization and Blinding)** – Somewhere in this section, suggest that you remind the reader that it is impossible to blind participants in this type of intervention study.

RESPONSE:

We thank the reviewer for this helpful suggestion. We agree that participant blinding was not feasible because the two intervention protocols differed in treatment duration. To improve transparency, we have clarified this point in the Randomization and Blinding section of the Methods.

**Changes made in the revised manuscript:**

**Methods**

**Randomization and Blinding**

**New version:**

**Additional text added** (Page 8, Lines 201-204).**:**

“Participant blinding was not feasible because the intervention protocols differed in treatment duration. To minimize expectation-related bias, participants were not informed of the study hypothesis or of any anticipated superiority between treatment protocols.”

**Strength and Limitations**

**New version:**

**Additional text added** (Page 20, Lines 516-519**:**

“Although outcome assessors remained blinded, participant blinding was not feasible because treatment duration differed between protocols, introducing the possibility of expectation-related bias. However, the inclusion of both subjective (VAS and NDI) and objective (PPT) outcomes may partially mitigate this concern.”

***Page 10, line 274 (Methods, Statistical Analysis)** – Since there are multiple methods available for intention-to-treat analyses, please describe the one you used and provide a citation for such. Also, was any adjustment made for the multiple statistical tests conducted? If so, then please describe. If not, then please state such. Finally, there was no interest in conducting per-protocol analysis but rather, limit to intention-to-treat analysis?

RESPONSE:

We thank the reviewer for this important comment. In response, we expanded the Statistical Analysis section to clarify the implementation of the intention-to-treat (ITT) analysis, the handling of multiplicity, and our approach to per-protocol analyses.

Specifically, we now state that all randomized participants were included in the primary analysis regardless of treatment completion status and that missing outcome data were handled using likelihood-based linear mixed-effects models estimated by restricted maximum likelihood (REML), which incorporate all available observations under the missing-at-random assumption without requiring complete-case exclusion [42].

We also clarified that pain intensity measured by the Visual Analog Scale (VAS) was the prespecified primary outcome and served as the basis for sample size estimation, whereas pressure pain threshold (PPT) and Neck Disability Index (NDI) were prespecified secondary outcomes. Because the primary hypothesis concerned the VAS outcome, no formal adjustment for multiplicity was applied, and findings relating to secondary outcomes and exploratory contrasts should therefore be interpreted cautiously.

Finally, we explicitly state that a per-protocol analysis was not prespecified and was not performed.

**Changes made in the revised manuscript:**

**Statistical Analysis**

**New version:**

**Additional text added** (Page 10 Lines 285-289)**:**

“All randomized participants were included in the primary analysis regardless of treatment completion status. Missing outcome data were handled using likelihood-based linear mixed-effects models estimated by restricted maximum likelihood (REML), which incorporate all available observations under the missing-at-random (MAR) assumption without requiring complete-case exclusion [42].”

**Additional reference added:**

42. White IR, Horton NJ, Carpenter J, Pocock SJ. Strategy for intention to treat analysis in randomised trials with missing outcome data. BMJ. 2011;342:d40.

**Additional text added** (Page 10, Lines 289)**:**

“A per-protocol analysis was not prespecified and was not performed.”

**Additional text added** (Page 10, Lines 293-294)**:**

“Because the primary hypothesis concerned the VAS outcome, no formal adjustment for multiplicity was applied..”

***Pages 12 through 14 (Results)** –In my opinion, you do a very nice job in reporting the statistical analysis of results for the VAS, Pressure Pain Threshold, and Neck Disability Index. However, for each of these I would suggest that you also tell the reader whether any changes met the clinically relevant thresholds for improvement and then in the Discussion section, mention such without using numbers.

RESPONSE:

We thank the reviewer for this valuable suggestion. In response, we revised the Discussion section to place greater emphasis on the clinical significance of the observed improvements. Specifically, we contextualized the changes in pain intensity, pressure pain threshold, and neck-related disability with reference to previously established thresholds for clinically meaningful improvement and measurement error reported in the literature [45–50]. We also clarified that improvements across all outcome domains exceeded these established thresholds.

Consistent with the reviewer’s recommendation, the revised Discussion emphasizes the clinical relevance of the findings without repeating detailed numerical results. Instead, it highlights that the observed improvements were clinically meaningful across multiple outcome domains and therefore likely to be relevant from a patient-centred and rehabilitation perspective.

**Changes made in the revised manuscript:**

**Discussion**

**Old version:** The Discussion primarily focused on statistical significance and magnitude of change. (Page 16-17, Lines 445-460)

**New version** (Page 19, Lines 468-477).**:**

“Irrespective of between-protocol differences, both treatment durations were associated with substantial and clinically meaningful improvements across multiple outcome domains, including pain intensity, mechanical sensitivity, and neck-related disability over the four-week treatment period. The magnitude of pain reduction exceeded commonly accepted thresholds for clinically meaningful improvement in chronic neck pain populations [45–47]. Likewise, improvements in PPT exceeded previously reported measurement error thresholds for upper trapezius trigger points [48,49], while reductions in neck-related disability surpassed accepted thresholds for meaningful functional improvement [46,50]. The convergence of subjective pain reduction, objective improvements in mechanical sensitivity, and functional recovery supports the clinical relevance of the observed improvements in individuals with upper trapezius MPS.”

***Pages 15 through 17, lines 406 through 473 (Discussion)** – For ease of reading, suggest that you partition your Discussion into the following subsections and address: (1) Overall findings, (2) Implications for research, (3) Implications for practice, and (4) Strengths and potential limitations.

RESPONSE:

We thank the reviewer for this helpful suggestion. In response, we reorganized the Discussion section into clearly defined subsections to improve readability and facilitate interpretation of the findings.

The revised Discussion is now structured into the following sections (Page 16-19, Lines 423-531):

1. Overall Findings

2. Implications for Research

3. Implications for Clinical Practice

4. Strengths and Limitations

This revised structure separates interpretation of the primary findings from their research and clinical implications while providing a dedicated section addressing study strengths and limitations. We believe that this organization improves clarity and enhances the accessibility of the manuscript.

***Page 17, lines 475 through 481 (Conclusions)** – Please move this section to the end of your manuscript.

RESPONSE:

We thank the reviewer for this helpful suggestion. The Conclusions section has been repositioned to the end of the main manuscript (Page 21, Lines 531-537).

***Page 17, lines 501 through 503 (Limitations)** – Another limitation to add here is that the results may not be generalizable to other patient populations.

RESPONSE:

We thank the reviewer for this helpful suggestion. We agree that the generalizability of the findings should be explicitly addressed as a study limitation.

In response, we expanded the Limitations section to clarify that the findings may not be generalizable beyond the specific study population and treatment setting. Specifically, we note that the study was conducted in adults aged 18 to 55 years with upper trapezius myofascial pain syndrome at a single rehabilitation centre using a predefined stimulation protocol. Therefore, the findings may not be generalizable to other patient populations, age groups, musculoskeletal conditions, healthcare settings, or alternative stimulation strategies.

**Changes made in the revised manuscript:**

**Strengths and Limitations**

**Old version:** “Third, the investigation focused on a single anatomical region and a predefined stimulation configuration, which may limit generalizability to other musculoskeletal conditions or alternative stimulation strategies.” (Page 18, Lines 520-523)

**New version:**

**Additional text added** (Page 19, Lines 521-524)**:**

“Third, the study included a specific patient population and stimulation protocol at a single rehabilitation centre, which may limit the generalizability of the findings to other patient populations, musculoskeletal conditions, healthcare settings or alternative stimulation protocols.”
