# Supplementary material for: Comparison of two repetitive peripheral magnetic stimulation protocols for upper trapezius myofascial pain syndrome: A randomized trial
Source: PLoS One. 2026 Oct 8;21(10):e0360355. doi: 10.1371/journal.pone.0360355 (PMC13648980; doi:10.1371/journal.pone.0360355)
Supplement: Supplementary file 5 [file pone.0360355.s006.docx]

**Dear Editor and Reviewers**

Many thanks for your detailed and constructive comments on our manuscript. We are pleased to provide a point-by-point response below. Changes in the revised manuscript are highlighted.

With best wishes.

Yours faithfully

The authors

**Reviewer #2**

I appreciate the opportunity to review revised manuscript PONE-D-26-17312R1 titled

"Comparison of Two Repetitive Peripheral Magnetic Stimulation Protocols for Upper Trapezius Myofascial Pain Syndrome: A Randomized Trial". Overall, the authors have done a very nice job in responding to me previous comments and suggestions. I only have one remaining comment, listed below.

**RESPONSE**

Thank you very much for your kind comments and suggestions. We sincerely appreciate your valuable feedback and guidance in improving the manuscript.

SPECIFIC COMMENT

*Page 8, lines 201 through 204 (Methods, Randomization and Blinding) – Thank you for responding to my previous suggestion. However, regardless of treatment duration, it is impossible to blind participants to the treatment they received in any type of human movement intervention. Thus, I would suggest that you add such. For example, maybe something like this: “Participant blinding was not feasible given that it is impossible to blind participants to these types of interventions. In addition, the treatment duration between the two groups differed.”

**RESPONSE**

Thank you very much for your helpful comment. We have revised the text accordingly to clarify that participant blinding was not feasible for this type of intervention. The revised sentence appears on page 8, lines 201–204:

“Group assignments were disclosed only after participant enrolment. Participant blinding was not feasible given that it is impossible to blind participants to these types of interventions. In addition, the treatment duration between the two groups differed...”
