# Supplementary material for: Comparison of two repetitive peripheral magnetic stimulation protocols for upper trapezius myofascial pain syndrome: A randomized trial
Source: PLoS One. 2026 Oct 8;21(10):e0360355. doi: 10.1371/journal.pone.0360355 (PMC13648980; doi:10.1371/journal.pone.0360355)
Supplement: Peer review history

# Peer review: Decision Letter 0

**DOI:** 10.1371/journal.pone.0360355.r001

**Authors:** Mohammad Sidiq

15 May 2026

Please submit your revised manuscript by Jun 29 2026 11:59PM. If you need more time than this to complete your revisions, please reply to this message or contact the journal office at plosone@plos.org. When you're ready to submit your revision, log on to https://www.editorialmanager.com/pone/ and select the 'Submissions Needing Revision' folder to locate your manuscript file.

- A letter that responds to each point raised by the academic editor and reviewer(s). You should upload this letter as a separate file labeled 'Response to Reviewers'.
- A marked-up copy of your manuscript that highlights changes made to the original version. You should upload this as a separate file labeled 'Revised Manuscript with Track Changes'.
- An unmarked version of your revised paper without tracked changes. You should upload this as a separate file labeled 'Manuscript'.

If you would like to make changes to your financial disclosure, please include your updated statement in your cover letter. Guidelines for resubmitting your figure files are available below the reviewer comments at the end of this letter.

If applicable, we recommend that you deposit your laboratory protocols in protocols.io to enhance the reproducibility of your results. Protocols.io assigns your protocol its own identifier (DOI) so that it can be cited independently in the future. For instructions see: https://journals.plos.org/plosone/s/submission-guidelines#loc-laboratory-protocols. Additionally, PLOS ONE offers an option for publishing peer-reviewed Lab Protocol articles, which describe protocols hosted on protocols.io. Read more information on sharing protocols at https://plos.org/protocols?utm_medium=editorial-email&utm_source=authorletters&utm_campaign=protocols.

As the corresponding author, your ORCID iD is verified in the submission system and will appear in the published article. PLOS supports the use of ORCID, and we encourage all coauthors to register for an ORCID iD and use it as well. Please encourage your coauthors to verify their ORCID iD within the submission system before final acceptance, as unverified ORCID iDs will not appear in the published article. *Only*  the individual author can complete the verification step; PLOS staff *cannot*  verify ORCID iDs on behalf of authors.

We look forward to receiving your revised manuscript.

Kind regards,

Mohammad Sidiq, PhD Physiotherapy, FAIMER Fellow

Academic Editor

PLOS One

Journal requirements:

When submitting your revision, we need you to address these additional requirements.

1. Please ensure that your manuscript meets PLOS ONE's style requirements, including those for file naming. The PLOS ONE style templates can be found at

https://journals.plos.org/plosone/s/file?id=wjVg/PLOSOne_formatting_sample_main_body.pdf and

https://journals.plos.org/plosone/s/file?id=ba62/PLOSOne_formatting_sample_title_authors_affiliations.pdf

If the reviewer comments include a recommendation to cite specific previously published works, please review and evaluate these publications to determine whether they are relevant and should be cited. There is no requirement to cite these works unless the editor has indicated otherwise.

[Note: HTML markup is below. Please do not edit.]

Reviewers' comments:

Reviewer's Responses to Questions

**Comments to the Author**

1. Is the manuscript technically sound, and do the data support the conclusions?

Reviewer #1: Partly

Reviewer #2: Yes

**********

2. Has the statistical analysis been performed appropriately and rigorously?

Reviewer #1: No

Reviewer #2: Yes

**********

3. Have the authors made all data underlying the findings in their manuscript fully available?

The PLOS Data policy

Reviewer #1: No

Reviewer #2: Yes

**********

4. Is the manuscript presented in an intelligible fashion and written in standard English?

Reviewer #1: Yes

Reviewer #2: Yes

**********

Reviewer #1: This manuscript presents a randomized controlled trial comparing two repetitive peripheral magnetic stimulation (rPMS) protocols (10-minute versus 15-minute sessions) for upper trapezius myofascial pain syndrome (MPS). The topic is clinically relevant, and the study addresses an important practical question regarding treatment parameter optimization. The manuscript is generally well organized, clearly written, and methodologically stronger than many small rehabilitation trials. The use of longitudinal mixed-effects modeling, assessor blinding, and intention-to-treat analysis are notable strengths.

However, several methodological and statistical concerns require clarification and revision before the manuscript can be considered for publication. In particular, the interpretation of non-significant findings as evidence of comparable efficacy is not fully supported by the study design or statistical framework. In addition, the data availability statement appears inconsistent with the materials currently provided.

Major Comments

1. Interpretation of Non-Significant Findings and Equivalence Claims

The principal concern relates to the interpretation of the results. The manuscript repeatedly implies that the 10-minute protocol is “comparable” or “equally effective” relative to the 15-minute protocol. However, the trial appears to have been designed and analyzed as a superiority study rather than an equivalence or noninferiority trial.

A non-significant protocol-by-time interaction does not establish equivalence between interventions. Demonstrating equivalence or noninferiority would require:

a prespecified equivalence/noninferiority margin,

dedicated sample size justification,

and appropriate equivalence/noninferiority statistical methodology.

At present, the findings support only the conclusion that no statistically significant difference was detected between protocols within the study sample and follow-up period.

2.Sample Size Justification Requires Clarification

The sample size calculation assumes a clinically meaningful between-group VAS difference of 0.5 points with SD = 0.8. Please provide stronger clinical justification for selecting a 0.5-point difference as the target effect size, as this appears smaller than commonly accepted minimal clinically important differences for neck pain outcomes.

Additionally, the manuscript states that sample size estimation was based on an ANCOVA framework, whereas the primary analysis used longitudinal mixed-effects modeling. Please clarify:

whether repeated-measures correlation was incorporated appropriately,

and whether the final analysis framework was considered during power estimation.

3.Lack of Sham or Usual-Care Control Group

The absence of a sham-control or exercise-only comparator substantially limits interpretation of treatment efficacy. Both groups received active rPMS plus stretching exercises; therefore, it is not possible to determine:

the magnitude of the specific rPMS effect,

the contribution of stretching,

or the role of contextual/placebo effects.

Although this limitation is acknowledged, it should be emphasized more clearly throughout the Discussion and Conclusions. The study should be framed primarily as a comparison of two active protocols rather than evidence confirming efficacy of rPMS itself.

4.Limited Separation Between Intervention Arms

The biological distinction between the two protocols may be insufficient to meaningfully evaluate dose-response effects. Both groups received the identical 5-minute targeted trigger-point stimulation phase, while only the scanning phase duration differed.

Please discuss more explicitly whether the focused trigger-point stimulation phase may represent the primary active component of the intervention, thereby limiting the ability to detect duration-dependent effects.

5. Missing Data Handling Requires Additional Transparency

The use of REML-based mixed models and MICE sensitivity analyses is appropriate. However, additional information regarding missingness is needed.

Please provide:

the number of missing observations by group and timepoint,

reasons for missingness,

and clarification regarding the plausibility of the MAR assumption.

It would also strengthen the manuscript to provide supplementary information regarding the imputation procedure and variables included in the MICE model.

6.Data Availability Statement Appears Incomplete

The manuscript states:

“All relevant data are within the manuscript and its Supporting Information files.”

However, no participant-level dataset, statistical code, or supplementary data files appear to be included in the submitted materials currently available for review.

Please ensure compliance with the journal’s data availability requirements by providing:

a de-identified participant-level dataset,

variable definitions/data dictionary,

and preferably the statistical analysis code or syntax.

If these materials are available elsewhere, please provide the repository link or accession information

Minor Comments

1.Clarify Blinding Limitations

The study is described as assessor-blinded; however, participants were likely aware of treatment duration differences. Please discuss the potential for performance and expectation bias more explicitly.

2.Adverse Event Reporting

The manuscript reports that no serious adverse events occurred, but it is unclear whether minor or transient adverse effects were monitored systematically.

Please clarify whether participants experienced:

discomfort,

muscle soreness,

headaches,

skin sensations,

or treatment intolerance.

3.Multiplicity of Statistical Testing

Multiple outcomes and repeated contrasts were analyzed without mention of multiplicity adjustment. While this may be acceptable for exploratory secondary analyses, the manuscript should clarify:

which endpoint was prespecified as primary,

and whether secondary analyses should be interpreted cautiously.

4.Mechanistic Interpretation Should Be Moderated

Several sections of the Introduction and Discussion contain extensive mechanistic interpretation regarding:

neuroplasticity,

cortical excitability,

central sensitization,

and descending inhibitory modulation.

These concepts are scientifically plausible but were not directly measured in this study. Please ensure that mechanistic statements remain appropriately speculative and hypothesis-generating.

5.Writing Style and Conciseness

The manuscript is generally well written and uses standard academic English appropriately. However, portions of the Introduction and Discussion are overly dense and could be streamlined for clarity and conciseness.

In particular:

mechanistic background sections are somewhat lengthy relative to the study question,

several concepts are repeated,

and some sentences could be shortened for readability.

A moderate reduction in length would improve accessibility without compromising scientific content.

Overall Recommendation

The manuscript addresses a clinically relevant question and demonstrates several methodological strengths, including randomized allocation, blinded assessment, and appropriate longitudinal modeling. However, revisions are needed to:

avoid equivalence overinterpretation,

improve statistical transparency,

clarify data availability,

and moderate mechanistic claims.

With appropriate revision, the study may provide a useful contribution to the rehabilitation and musculoskeletal pain literature.

Reviewer #2: GENERAL COMMENTS

I appreciate the opportunity to review manuscript #PONE-D-26-17312 titled "Effect of two repetitive peripheral magnetic stimulation protocols on upper trapezius myofascial pain syndrome: a randomized controlled trial". I have several comments and suggestions, detailed in my specific comments that follow, that I believe will strengthen this work.

SPECIFIC COMMENTS

*Page 1, lines 2 and 3 (Title) – Since there was no true control group, please replace “randomized controlled trial” with “randomized trial” here and throughout the rest of the manuscript.

*Page 2, line 45 (Abstract, Results) – Suggest you replace “per protocol” with “per group”.

*Page 6, line 1 (Methods, Study Design) – What was the rationale for not including a true control group that received no intervention or some type of sham intervention?

*Page 7, lines 171 through 176 (Methods, Sample size) – First, please tell the reader if your sample size estimate was based on your primary outcome, pain intensity using the Visual Analog Scale, or something else, for example, some type of standardized effect size. Second, please clarify for the reader whether a clinically meaningful between-group difference of 0.5 points was based on the VAS or something else.

*Page 7, lines 183 through 191 (Methods, Randomization and Blinding) – Somewhere in this section, suggest that you remind the reader that it is impossible to blind participants in this type of intervention study.

*Page 10, line 274 (Methods, Statistical Analysis) – Since there are multiple methods available for intention-to-treat analyses, please describe the one you used and provide a citation for such. Also, was any adjustment made for the multiple statistical tests conducted? If so, then please describe. If not, then please state such. Finally, there was no interest in conducting per-protocol analysis but rather, limit to intention-to-treat analysis?

*Pages 12 through 14 (Results) –In my opinion, you do a very nice job in reporting the statistical analysis of results for the VAS, Pressure Pain Threshold, and Neck Disability Index. However, for each of these I would suggest that you also tell the reader whether any changes met the clinically relevant thresholds for improvement and then in the Discussion section, mention such without using numbers.

*Pages 15 through 17, lines 406 through 473 (Discussion) – For ease of reading, suggest that you partition your Discussion into the following subsections and address: (1) Overall findings, (2) Implications for research, (3) Implications for practice, and (4) Strengths and potential limitations.

*Page 17, lines 475 through 481 (Conclusions) – Please move this section to the end of your manuscript.

*Page 17, lines 501 through 503 (Limitations) – Another limitation to add here is that the results may not be generalizable to other patient populations.

**********

what does this mean?). If published, this will include your full peer review and any attached files.

If you choose “no”, your identity will remain anonymous but your review may still be made public.

**Do you want your identity to be public for this peer review?**  For information about this choice, including consent withdrawal, please see our Privacy Policy

Reviewer #1: No

Reviewer #2: No

**********

[NOTE: If reviewer comments were submitted as an attachment file, they will be attached to this email and accessible via the submission site. Please log into your account, locate the manuscript record, and check for the action link "View Attachments". If this link does not appear, there are no attachment files.]

To ensure your figures meet our technical requirements, please review our figure guidelines: https://journals.plos.org/plosone/s/figures

You may also use PLOS’s free figure tool, NAAS, to help you prepare publication quality figures: https://journals.plos.org/plosone/s/figures#loc-tools-for-figure-preparation.

NAAS will assess whether your figures meet our technical requirements by comparing each figure against our figure specifications.

# Author comment: Author response to Decision Letter 1

**DOI:** 10.1371/journal.pone.0360355.r002

21 Jun 2026

Please see the attached file.

Attachment Submitted filename: ResponsetorefereeV1_2006.docx

# Peer review: Decision Letter 1

**DOI:** 10.1371/journal.pone.0360355.r003

**Authors:** Mohammad Sidiq

5 Aug 2026

Dear Dr. Saengsuwan,

Thank you for submitting your manuscript to PLOS ONE. After careful consideration, we feel that it has merit but does not fully meet PLOS ONE’s publication criteria as it currently stands. Therefore, we invite you to submit a revised version of the manuscript that addresses the points raised during the review process.

Please submit your revised manuscript by Sep 19 2026 11:59PM. If you will need more time than this to complete your revisions, please reply to this message or contact the journal office at plosone@plos.org. When you're ready to submit your revision, log on to https://www.editorialmanager.com/pone/ and select the 'Submissions Needing Revision' folder to locate your manuscript file.

- A letter that responds to each point raised by the academic editor and reviewer(s). You should upload this letter as a separate file labeled 'Response to Reviewers'.
- A marked-up copy of your manuscript that highlights changes made to the original version. You should upload this as a separate file labeled 'Revised Manuscript with Track Changes'.
- An unmarked version of your revised paper without tracked changes. You should upload this as a separate file labeled 'Manuscript'.

If you would like to make changes to your financial disclosure, please include your updated statement in your cover letter. Guidelines for resubmitting your figure files are available below the reviewer comments at the end of this letter.

If applicable, we recommend that you deposit your laboratory protocols in protocols.io to enhance the reproducibility of your results. Protocols.io assigns your protocol its own identifier (DOI) so that it can be cited independently in the future. For instructions see: https://journals.plos.org/plosone/s/submission-guidelines#loc-laboratory-protocols. Additionally, PLOS ONE offers an option for publishing peer-reviewed Lab Protocol articles, which describe protocols hosted on protocols.io. Read more information on sharing protocols at https://plos.org/protocols?utm_medium=editorial-email&utm_source=authorletters&utm_campaign=protocols.

As the corresponding author, your ORCID iD is verified in the submission system and will appear in the published article. PLOS supports the use of ORCID, and we encourage all coauthors to register for an ORCID iD and use it as well. Please encourage your coauthors to verify their ORCID iD within the submission system before final acceptance, as unverified ORCID iDs will not appear in the published article. *Only*  the individual author can complete the verification step; PLOS staff *cannot*  verify ORCID iDs on behalf of authors.

We look forward to receiving your revised manuscript.

Kind regards,

Mohammad Sidiq, PhD Physiotherapy, FAIMER Fellow

Academic Editor

PLOS One

Journal Requirements:

If the reviewer comments include a recommendation to cite specific previously published works, please review and evaluate these publications to determine whether they are relevant and should be cited. There is no requirement to cite these works unless the editor has indicated otherwise.

Please review your reference list to ensure that it is complete and correct. If you have cited papers that have been retracted, please include the rationale for doing so in the manuscript text, or remove these references and replace them with relevant current references. Any changes to the reference list should be mentioned in the rebuttal letter that accompanies your revised manuscript. If you need to cite a retracted article, indicate the article’s retracted status in the References list and also include a citation and full reference for the retraction notice.

[Note: HTML markup is below. Please do not edit.]

Reviewers' comments:

Reviewer's Responses to Questions

**Comments to the Author**

Reviewer #1: All comments have been addressed

Reviewer #2: (No Response)

**********

2. Is the manuscript technically sound, and do the data support the conclusions?

Reviewer #1: Yes

Reviewer #2: Yes

**********

3. Has the statistical analysis been performed appropriately and rigorously?

Reviewer #1: Yes

Reviewer #2: Yes

**********

4. Have the authors made all data underlying the findings in their manuscript fully available?

The PLOS Data policy

Reviewer #1: Yes

Reviewer #2: Yes

**********

5. Is the manuscript presented in an intelligible fashion and written in standard English?

Reviewer #1: Yes

Reviewer #2: Yes

**********

Reviewer #1: I appreciate the authors' careful revisions and detailed responses to the review comments. All of my major and minor concerns have been adequately addressed, and the manuscript has improved accordingly. I have no additional comments and recommend acceptance.

Reviewer #2: GENERAL COMMENTS

I appreciate the opportunity to review revised manuscript PONE-D-26-17312R1 titled

"Comparison of Two Repetitive Peripheral Magnetic Stimulation Protocols for Upper Trapezius Myofascial Pain Syndrome: A Randomized Trial". Overall, the authors have done a very nice job in responding to me previous comments and suggestions. I only have one remaining comment, listed below.

SPECIFIC COMMENT

*Page 8, lines 201 through 204 (Methods, Randomization and Blinding) – Thank you for responding to my previous suggestion. However, regardless of treatment duration, it is impossible to blind participants to the treatment they received in any type of human movement intervention. Thus, I would suggest that you add such. For example, maybe something like this: “Participant blinding was not feasible given that it is impossible to blind participants to these types of interventions. In addition, the treatment duration between the two groups differed.”

END OF REVIEW

**********

what does this mean?). If published, this will include your full peer review and any attached files.

If you choose “no”, your identity will remain anonymous but your review may still be made public.

**Do you want your identity to be public for this peer review?**  For information about this choice, including consent withdrawal, please see our Privacy Policy

Reviewer #1: No

Reviewer #2: No

**********

[NOTE: If reviewer comments were submitted as an attachment file, they will be attached to this email and accessible via the submission site. Please log into your account, locate the manuscript record, and check for the action link "View Attachments". If this link does not appear, there are no attachment files.]

To ensure your figures meet our technical requirements, please review our figure guidelines: https://journals.plos.org/plosone/s/figures

You may also use PLOS’s free figure tool, NAAS, to help you prepare publication quality figures: https://journals.plos.org/plosone/s/figures#loc-tools-for-figure-preparation.

NAAS will assess whether your figures meet our technical requirements by comparing each figure against our figure specifications.

# Author comment: Author response to Decision Letter 2

**DOI:** 10.1371/journal.pone.0360355.r004

10 Aug 2026

Reviewer #2

I appreciate the opportunity to review revised manuscript PONE-D-26-17312R1 titled

"Comparison of Two Repetitive Peripheral Magnetic Stimulation Protocols for Upper Trapezius Myofascial Pain Syndrome: A Randomized Trial". Overall, the authors have done a very nice job in responding to me previous comments and suggestions. I only have one remaining comment, listed below.

RESPONSE

Thank you very much for your kind comments and suggestions. We sincerely appreciate your valuable feedback and guidance in improving the manuscript.

SPECIFIC COMMENT

*Page 8, lines 201 through 204 (Methods, Randomization and Blinding) – Thank you for responding to my previous suggestion. However, regardless of treatment duration, it is impossible to blind participants to the treatment they received in any type of human movement intervention. Thus, I would suggest that you add such. For example, maybe something like this: “Participant blinding was not feasible given that it is impossible to blind participants to these types of interventions. In addition, the treatment duration between the two groups differed.”

RESPONSE

Thank you very much for your helpful comment. We have revised the text accordingly to clarify that participant blinding was not feasible for this type of intervention. The revised sentence appears on page 8, lines 201–204:

“Group assignments were disclosed only after participant enrolment. Participant blinding was not feasible given that it is impossible to blind participants to these types of interventions. In addition, the treatment duration between the two groups differed...”

Attachment Submitted filename: ResponsetorefereeV2_0708.docx

# Peer review: Decision Letter 2

**DOI:** 10.1371/journal.pone.0360355.r005

**Authors:** Mohammad Sidiq

27 Aug 2026

Comparison of Two Repetitive Peripheral Magnetic Stimulation Protocols for Upper Trapezius Myofascial Pain Syndrome: A Randomized Trial

PONE-D-26-17312R2

Dear Saengsuwan,

We’re pleased to inform you that your manuscript has been judged scientifically suitable for publication and will be formally accepted for publication once it meets all outstanding technical requirements.

Within one week, you’ll receive an e-mail detailing the required amendments. When these have been addressed, you’ll receive a formal acceptance letter, and your manuscript will be scheduled for publication.

An invoice will be generated when your article is formally accepted. Please note, if your institution has a publishing partnership with PLOS and your article meets the relevant criteria, all or part of your publication costs will be covered. Please make sure your user information is up-to-date by logging into Editorial Manager at Editorial Manager® and clicking the ‘Update My Information' link at the top of the page. For questions related to billing, please contact billing support.

Mohammad Sidiq, PhD Physiotherapy, FAIMER Fellow

Academic Editor

PLOS One

Additional Editor Comments (optional):

Dear authors, I and the reviewers have given recommendations for the acceptance; we do not have any further comments. Hence, I recommend it to be accepted in its current form. Further procedure will follow from the editorial board.

Reviewers' comments:

Reviewer's Responses to Questions

**Comments to the Author**

Reviewer #2: All comments have been addressed

**********

2. Is the manuscript technically sound, and do the data support the conclusions?

Reviewer #2: Yes

**********

3. Has the statistical analysis been performed appropriately and rigorously?

Reviewer #2: Yes

**********

4. Have the authors made all data underlying the findings in their manuscript fully available?

The PLOS Data policy

Reviewer #2: Yes

**********

5. Is the manuscript presented in an intelligible fashion and written in standard English?

Reviewer #2: Yes

**********

Reviewer #2: GENERAL COMMENTS

I appreciate the opportunity to review revised manuscript PONE-D-26-17312R2 titled

"Comparison of Two Repetitive Peripheral Magnetic Stimulation Protocols for Upper Trapezius Myofascial Pain Syndrome: A Randomized Trial". The authors have responded adequately to my one previous suggestion. I have no further comments or suggestions for improvement.

END OF REVIEW

**********

what does this mean?). If published, this will include your full peer review and any attached files.

If you choose “no”, your identity will remain anonymous but your review may still be made public.

**Do you want your identity to be public for this peer review?**  For information about this choice, including consent withdrawal, please see our Privacy Policy

Reviewer #2: No

**********

# Editor report: Acceptance letter

**DOI:** 10.1371/journal.pone.0360355.r006

**Authors:** Mohammad Sidiq

PONE-D-26-17312R2

PLOS One

Dear Dr. Saengsuwan,

I'm pleased to inform you that your manuscript has been deemed suitable for publication in PLOS One. Congratulations! Your manuscript is now being handed over to our production team.

At this stage, our production department will prepare your paper for publication. This includes ensuring the following:

* All references, tables, and figures are properly cited

* All relevant supporting information is included in the manuscript submission,

* There are no issues that prevent the paper from being properly typeset

You will receive further instructions from the production team, including instructions on how to review your proof when it is ready. Please keep in mind that we are working through a large volume of accepted articles, so please give us a few days to review your paper and let you know the next and final steps.

Lastly, if your institution or institutions have a press office, please let them know about your upcoming paper now to help maximize its impact. If they'll be preparing press materials, please inform our press team within the next 48 hours. Your manuscript will remain under strict press embargo until 2 pm Eastern Time on the date of publication. For more information, please contact onepress@plos.org.

You will receive an invoice from PLOS for your publication fee after your manuscript has reached the completed accept phase. If you receive an email requesting payment before acceptance or for any other service, this may be a phishing scheme. Learn how to identify phishing emails and protect your accounts at https://explore.plos.org/phishing.

If we can help with anything else, please email us at customercare@plos.org.

Thank you for submitting your work to PLOS One and supporting open access.

Kind regards,

PLOS One Editorial Office Staff

on behalf of

Dr. Mohammad Sidiq

Academic Editor

PLOS One
